# The effectiveness of telepsychiatry: thematic review

**DOI:** 10.1192/bjb.2021.115

**Published:** 2023-04

**Authors:** Gunjan Sharma, Karrish Devan

**Affiliations:** South London and Maudsley NHS Foundation Trust, UK

**Keywords:** Community mental health teams, information technologies, out-patient treatment, in-patient treatment, education and training

## Abstract

**Aims and method:**

This review aims to clarify the evidence on the effectiveness of telepsychiatry following the COVID-19 pandemic. We conducted a literature review of three databases (Cochrane Library, PubMed and PsycINFO), using the terms virtual consultation/telepsychiatry/video consultation AND psychiatry/mental illness.

**Results:**

We identified 325 eligible papers and conducted a thematic analysis resulting in five themes: patient and clinical satisfaction, diagnostic reliability, outcomes, technology and professional guidance. The most significant factors linked to effectiveness of telepsychiatry were patient and clinician satisfaction and adequate technology to facilitate examination of the patient.

**Clinical implications:**

The consistent diagnostic reliability, satisfactory clinical outcomes and patient satisfaction linked to telepsychiatry favour its continued use once the pandemic ends. The main barrier is reluctance among clinicians and lack of professional guidance. We recommend education on the uses of telepsychiatry among clinicians, and the provision of professional guidance for its use from medical bodies and organisations.

Telepsychiatry has its roots from as far back as 1959, when live video-conferencing experiments were conducted in the USA for both patients and medical students.^1^ The term telepsychiatry itself was coined in 1973,^2^ but it was not until the 1990s when there was a noted increase in published research in the field.^3^ Perhaps reflecting this long history, there is no set definition for the term telepsychiatry; existing research using the term can encapsulate consultations in either real time or with a delay (synchronous versus asynchronous), and via a variety of media (virtual platforms, telephone, emails, messaging apps or texting).^2–4^

The most recent systematic review obtainable was published in 2015 and found insufficient evidence in regards to the effectiveness of telepsychiatry, based on ten randomised controlled trials (RCTs).^5^ More recent literature suggests that psychiatry may be uniquely suited to technological assessments because of a number of factors, including a global rise in mental health issues, shortage of trained professionals and communication being at the heart of the speciality.^1–6^

An unexpected change from the COVID-19 pandemic has been the boom in the use of technology to enable clinicians and patients to communicate safely and effectively.^7^ It appears unlikely that use of telepsychiatry will reduce post-COVID-19, as it becomes increasingly embedded into everyday practice.^8^

In light of these considerations, this thematic review looks at the effectiveness of telepsychiatry, with effectiveness defined in terms of patient and clinician perspectives, accessibility and clinical outcomes.^9,10^

## Method

A pilot review was conducted by both researchers, using six databases and nine terms to gain an understanding of the literature and feasibility of the study. Following this pilot, the authors agreed to focus on three databases that were deemed to be most suitable for the subject area: Cochrane Library, PsycINFO and PubMed. The authors agreed on the following search terms based on the pilot review: virtual consultation/telepsychiatry/video consultation AND psychiatry/mental illness. This allowed a balance between an adequate overview of the topic (following the pilot review) and practical aspects of having two researchers. The full details of the thematic review can be found in Supplementary Appendix 3 available at https://doi.org/10.1192/bjb.2021.115.

The inclusion criteria for this paper were articles that were published in English and focused on both the psychiatric consultation and clinical effectiveness of telepsychiatry. Exclusion criteria were: papers not in English, papers that did not focus on telepsychiatry defined as a video consultation (i.e. not telephone, text or email) and papers that focused on the non-psychiatric consultation such as psychological therapies. The authors agreed to include all types of papers to cover the breadth of the literature and in keeping with the thematic style of the review. All three databases were searched separately by both authors, and abstracts were reviewed based on the inclusion and exclusion criteria. Each author reviewed all abstracts and discussed any disagreements, to ensure quality control.

The authors undertook thematic analysis to analyse the data; full description of this analysis can be found in Supplementary Appendix 3. The authors chose thematic analysis as the most appropriate methodology for a number of reasons, including the breadth and heterogeneity of the data, for which thematic analysis is known to be more suitable.^11^ The authors also noted that the majority of previous reviews in this area focused entirely on RCTs; the authors wished to gain a wider perspective on the literature, particularly given the dearth of RCTs around the topic and other, non-RCT sources of information and research. This heterogeneity made thematic analysis the most appropriate method of analysing the data.^11^

In this study, the term themes refers to ‘actively constructed patterns (or meanings) derived from a data set that answer a research question, as opposed to mere summaries or categorizations of codes’.^12^

## Results

**Fig. 1:**
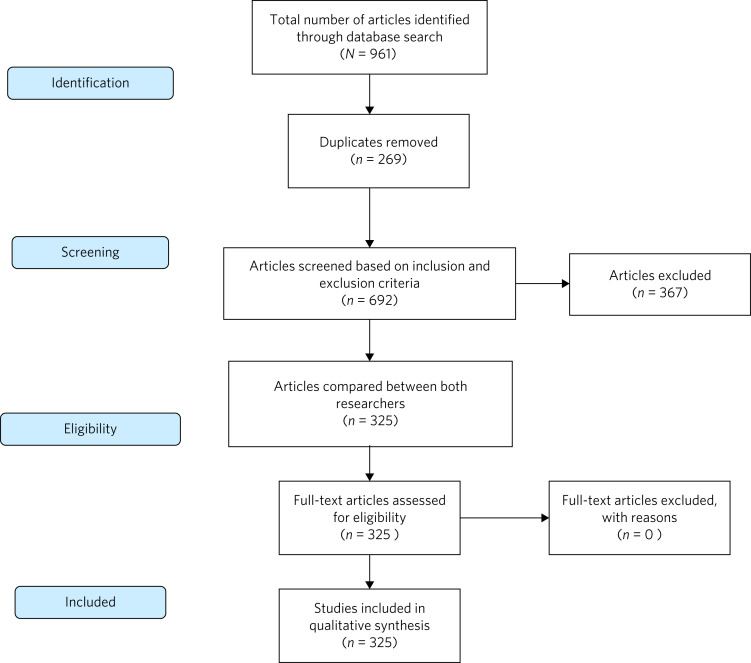
Preferred Reporting Items for Systematic reviews and Meta-Analyses flow chart of study methodology.

A total of 961 records were identified with the database searches (32 from the Cochrane Library, 494 from PubMed and 435 from PsycINFO). Of these, 269 duplicates were removed (Fig. 1).

The remaining 692 papers were divided randomly in half and each author reviewed 346 papers. After full review of the paper, 367 were excluded for not matching the inclusion criteria, resulting in a total of 325 papers.

The papers in this review spanned 24 years of research from 1996–2020, and a wide range of methodologies were included, as illustrated in Table 1.

**Table 1:** Types of studies in review

Type of study	Number of papers in review
Systematic review	17
Literature review	32
Narrative review	19
Scoping review	2
Randomised controlled trial	14
Prospective study	17
Retrospective study	14
Case–control study	1
Cross-sectional study	3
Longitudinal study	1
Epidemiological study	1
Observational study	1
Qualitative study	29
Feasibility study	1
Ethnographic study	1
Economic study	4
Case report	7
Service evaluation	49
Commentary	63
Correspondence	5
Letter to the Editor	17
Editorial	9
‘Lessons Learnt’ from randomised controlled trials	1
Book chapter	9
Pilot study	1
Guidelines	4
‘Overview’	1
Description of creating a study	1
Proposal	1

**Fig. 2:**
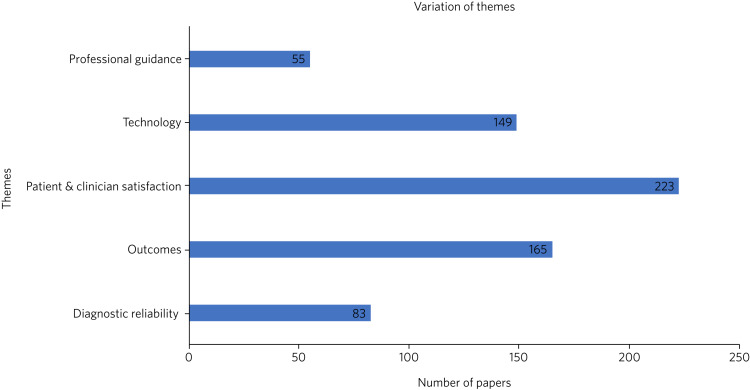
Thematic findings of review.

These papers were reviewed by both authors individually, for identification of themes related to the question of the effectiveness of telepsychiatry. No further papers were removed. The authors found five consistent themes throughout the papers that were linked to effectiveness: patient and clinician satisfaction, technology, diagnostic reliability, outcomes and professional guidance (Fig. 2). The full list of papers can be found in Supplementary Appendix 2, Table 3.

### Patient and clinician satisfaction

#### Patient satisfaction

The review found consistently high levels of patient satisfaction with telepsychiatry, with little difference between virtual and face-to-face consultations. Two papers reported that even during periods of relapse, patients are willing to use telepsychiatry.

Patient satisfaction was found to be particularly high among children and adolescents, possibly because of the familiarity with technology among this age group. Some studies found that children found the experience of the virtual consultation less threatening than a face-to-face consultation. Indeed, patients have reported preferring virtual consultations because of the ease of discussing sensitive topics, and certain patient groups have reported finding telepsychiatry more comfortable to engage with, such as individuals with autism spectrum disorder, Tourette's and those who generally find it to more difficult engage with face-to-face consultations. Other vulnerable groups were reported to benefit from telepsychiatry, including those with limited mobility/ability to travel, those living in rural areas and those within prison and forensic settings, with a number of studies specifically commenting on the increased access to mental health services via telepsychiatry within prison and forensic settings.

From a patient perspective, there were few criticisms of telepsychiatry. Studies found that some patients may perceive virtual consultations as ‘second rate’, feeling the clinician does not care enough about the patient to offer a face-to-face consultation or that they are missing out by not seeing a psychiatrist face to face. Only one study explored patient concerns about confidentiality and privacy on the virtual platforms.

#### Professional satisfaction

Professional satisfaction is not as consistent as patient satisfaction, with studies reporting both low and high satisfaction rates**.**

A number of studies suggested that professional perception was likely the biggest barrier toward implementation of telepsychiatry, with clinicians reporting concerns around the therapeutic alliance, data security and confidentiality, a lack of familiarity with technology and habit/historical practice. One of the specific concerns from clinicians was the belief that Telepsychiatry may negatively affect the ability to form rapport with patients.

However, once clinicians trialled telepsychiatry they became more positive about its uses, with reports of increased ability to care for patients through easier access, reduction in waiting times and improved service quality.

### Technology

Studies have consistently shown that technology has a crucial impact on the effectiveness of telepsychiatry, with limitations owing to a lack of audio quality and internet connection difficulties. Concerns have also been raised around visual quality, which can affect the clinicians ability to conduct an adequate mental state examination.

Only a few studies reported found that participants did not experience substantial difficulties with technology when using telepsychiatry, and some papers commented on the potential reduction in technological issues as costs reduce and advancements continue in this field.

### Diagnostic reliability

Studies have found high rates of diagnostic reliability between virtual and face-to-face consultations in a variety of psychiatric disorders, including post-traumatic stress disorder, depression, substance misuse disorders, intellectual disabilities, schizophrenia, attention-deficit hyperactivity disorder and dementia, assessing these though across multiple different standardised and unstandardised measures. A small number of papers looked at risk assessments conducted virtually and found that such assessments were equivalent in reliability to those conducted face to face. Reliability has also been shown to be high across age groups.

One study raised concerns about the diagnostic reliability of telepsychiatry when used to assess certain signs and symptoms such as the negative symptoms of schizophrenia, which may be harder to elicit virtually because they often require a good relationship and understanding of the patient over a period of time.

### Professional guidance

Many studies raised concerns about the lack of professional guidance on the use of telepsychiatry. The most prominent concern was the need for guidelines around appropriate prescriptions for patients who may be in a different geographical location with treatment guidelines different from the clinician's own. Other concerns included lack of protocols for emergencies, data security breaches and issues with consent. This lack of guidance was found to negatively affect professional perceptions of telepsychiatry and was a potential barrier to implementation of telepsychiatry. In recent years, some guidance has been made available, such as the guidance from the American Psychiatric Association and the General Medical Council in the UK.

### Clinical outcomes

A wide range of papers found that telepsychiatry can reduce symptoms of mental illness and improve functioning equivalent to face-to-face consultations. These positive results have been shown for a range of mental health problems, including post-traumatic stress disorder, anxiety, depression, substance misuse disorders, schizophrenia, attention-deficit hyperactivity disorder, autism spectrum disorders, panic disorders and agoraphobia. Specifically, in depression, telepsychiatry was reported to improve medication adherence and quality-of-life scoring, preventing relapses and recurrences.

Telepsychiatry has been shown to improve outcomes in other settings, with a reduction in in-patient admissions and reduced waiting times for patients awaiting psychiatric input in emergency departments. Two studies found positive outcomes when telepsychiatry was implemented within forensic services, such as a reduction in violent incidents.

Telepsychiatry has been found to increase access to specialised services, resulting in greater access to treatment and less distress for patients and their families. Many studies suggested that telepsychiatry could avoid psychiatric admissions by providing earlier access to care in areas where access to psychiatric services would otherwise be scarce. Other outcomes have also been noted to improve with the use of telepsychiatry, such as a reduction in non-attendance in virtual consultations compared with face-to-face consultations.

## Discussion

### Patient and clinician satisfaction

Perhaps unsurprisingly, satisfaction with telepsychiatry was found to be one of the most prominent themes in relation to effectiveness. Our results showed a discrepancy between patients’ and clinicians’ perspectives on telepsychiatry: patients consistently reported high levels of satisfaction, whereas clinicians were more varied. Patients appear to be satisfied as they place more emphasis on the reduced waiting times, increased flexibility and reduced need to travel,^3,13–17^ whereas clinicians are more concerned with the translation of the doctor–patient relationship to the screen and a lack of familiarity with technology.^18–30^

It is important to note that once clinicians integrate telepsychiatry into their practice, many change their minds and perceive telepsychiatry to be effective for psychiatric consultations.^1,10,13,18,19,31–44^ The results of the review suggest that these concerns are not a justifiable barrier to telepsychiatry, and increased awareness and education of the uses of telepsychiatry could break this patient–clinician barrier.

### Technology

The results of this review show that this debate around the use of telepsychiatry predates the COVID-19 pandemic; the earliest papers found in this study were from 1996.^22,45^ These early studies highlight the cost and physical size of historical technology as a significant limiting factor in their use. Our findings suggest that telepsychiatry is still limited in its effectiveness, both by the quality of hardware and strength of internet connection.

Modern hardware for virtual interactions may be cheaper and easier to get hold of, but real and perceived disturbances of audio information is a prominent feature of concern in our findings. Difficulties in hearing and understanding the other person's speech may affect the therapeutic alliance and the empathy one is able to display.^10,19,46^ Broken audio may be as limiting virtually; for example, when attempting a sensitive conversation, broken audio may be as disruptive as loud building work outside a clinic room would be in real life.

Internet connectivity remains another key limiting factor for the effectiveness of telepsychiatry. Without a stable internet connection, the quality of the hardware is almost irrelevant. With high demand on hospital bandwidth and those of clinicians working from home, internet connectivity must be considered for future planning of telepsychiatry. It is rare for clinicians not to experience or report some form of difficulty associated with technology when using telepsychiatry.^3,47–54^ The aim for virtual consultations to be an invisible interface between client and clinician is still very far away.^30^

Although not directly discussed in the findings, it is also important to take into consideration the utilisation of telepsychiatry for those who may not have access to good-quality hardware, software or who are limited in their physical access to the internet.

### Diagnostic reliability

Our findings found that telepsychiatry was reliable in diagnosing common psychiatric disorders equivalent to face-to-face consultations, albeit with a wide variety of diagnostic measures being used across different studies. This reliability did, however, remain consistent for a wide variety of mental illnesses, including psychosis, mood disorders, intellectual disabilities, substance misuse disorders and cognitive impairments, as well as assessments of risk. Thus, it could be used in a variety of mental health services and for a wide range of patients who may not be able to access face-to-face consultations, and not just for out-patient clinics, as is often assumed. Other examples noted in this study include prisons^55,56^ and emergency departments.^9,31,32,57–60^

### Outcomes

The ability to treat symptoms of mental illness and improve social functioning is a vital part of psychiatric practice, and studies have found that telepsychiatry has a largely positive effect on clinical outcomes across a wide range of conditions. It appears that by offering patients the choice of telepsychiatry versus traditional face-to-face appointments, end outcome measures are unlikely to change.

Telepsychiatry also offers an ability to change outcomes in different clinical settings, especially areas considered previously hard to reach, such as prisons and rural areas. There is the potential for telepsychiatry to offer a more equal sharing of resources. Within acute in-patient settings, studies found that telepsychiatry reduced outcomes associated with patient flow.^9,18,31,61–69^ Not only did clinicians and patients using telepsychiatry benefit, but widespread effects were also observed, such as a reduction in restrictive practice and bed occupancy.^31,62^

It is interesting to note that these outcomes went against the professional expectation.^19,22,24–28^ It is worth reflecting on why exactly there was so much professional resistance to telepsychiatry. Research suggests that clinicians are often hesitant to use telepsychiatry because they feel it would negatively affect outcomes.^33–35,45,62,70–74^ This remains a barrier to any new implementation in healthcare. However, as our healthcare systems have shown during the COVID-19 pandemic, many bureaucratic restrictions have been lifted and there is perhaps great scope for clinicians to use telepsychiatry in new and novel ways, with the knowledge that previous research has shown its efficacy with clinical outcomes.

### Guidance and ethical issues

Across the world, telepsychiatry offers opportunities for psychiatry to reach areas with poor provision in more direct and sustainable ways. However, clinicians are unwilling to engage with assessments if they occur outside of their local area, where they may be unfamiliar with policy, safeguarding and prescription guidelines.^10,19,24,27,45,63,64,75^ Studies from this review routinely raised concerns of the limits of telepsychiatry where there is a lack of professional guidance. Of the few current guidelines that do exist, it is worth noting that these were largely generated in the wake of the COVID-19 pandemic and are often non-specifically worded.^76–80^ Unless further detailed guidelines are created, clinicians run the risk of exposure to legal issues as they practice in areas with vastly different laws, customs and structures. Valid concerns remain that telepsychiatry could be used to fill gaps in rotas, stretch staff further and do little to address systemic staffing issues across the mental health profession.

It is not only clinical issues that arise from the lack of guidance, but ethical ones too. Patients may be exposed to the possibility of data leaks, being overhead within their own homes/environments or through compromised security. This represents a complex barrier to implementation, although COVID-19 has accelerated attempts to generate guidance. It is extremely unlikely that post-pandemic psychiatry will return in the same manner as before, now that telepsychiatry is becoming well established. To ensure that clinicians and patients are working safely and to appropriate standards, any future guidelines will have to also capture how telepsychiatry will work going forward, as hospitals, healthcare systems and countries gradually return to their usual legal and bureaucratic frameworks.

### Limitations

The main limitations of this review are the quality of the papers that were collected, publication bias and language bias. As highlighted in Table 1, the majority of the papers were service evaluations or commentaries with very few clinical studies or RCTs. The clinical studies included small sample sizes, with a number of case reports, descriptive studies and uncontrolled studies.^18^ Most clinical studies were done in controlled settings, making it difficult to generalise to clinical areas,^1^ and the ones that were conducted in clinical areas often included heterogeneous patient groups, making them difficult to generalise. Given the high number of positive studies in the area of telepsychiatry, some have even suggested the possibility of publication bias,^81^ something that the authors have also noted, with descriptive studies more likely to show positive outcomes than qualitative or experimental studies.^25^

The thematic analysis undertaken by the authors is also acknowledged to have its own limitations, such as its flexibility, which, although appropriate when dealing with heterogenous and large data-sets, can also make it vulnerable to inconsistency.^11^ The authors attempted to minimise this through peer debriefing, reflexivity and researcher triangulation; the full details of this can be found in Supplementary Appendix 3.

Finally, the vast majority of the literature in this area is from English-speaking countries (UK, USA, Australia and Canada), forming a potential skew on the themes in relation to socioeconomic factors, access and perceptions of technologies, and funding for mental health services.^82^

In conclusion, the literature captured in this thematic review suggests that telepsychiatry is effective. This is especially marked by high levels of patient satisfaction, diagnostic reliability and clinical outcomes, with the use of appropriate technology. It is clear from our results that the general public are both comfortable with and willing to use telepsychiatry.

It must be noted that the main barrier toward telepsychiatry is not the lack of evidence, but rather the reluctance among clinicians to facilitate telepsychiatry into their practice. This appears to often be a result of cynicism and a lack of familiarity; most likely this professional fear is further compounded by the lack of consistent professional guidance. Professional bodies should begin to increase guidance in this area, with emphasis on clinician concerns such as confidentiality, consent and emergencies. However, such policies take time to develop, and the psychiatric profession must consider the impact of our professional hesitation on the wishes of patients and their families.

## Data Availability

Data availability is not applicable to this article as no new data were created or analysed in this study.

## Supplementary material

For supplementary material accompanying this paper visit https://doi.org/10.1192/bjb.2021.115. click here to view supplementary material

## About the authors

**Gunjan Sharma** is a CT3 Psychiatry Trainee working at South London and Maudsley NHS Foundation Trust, UK. **Karrish Devan** is a CT3 Psychiatry Trainee at working at South London and Maudsley NHS Foundation Trust, UK.
