# Supplementary material for: The effectiveness of telepsychiatry: thematic review
Source: BJPsych Bull. 2023 Apr;47(2):82–9. doi: 10.1192/bjb.2021.115 (PMC10063994; doi:10.1192/bjb.2021.115)
Supplement: Supplementary file 1 [file S2056469421001157sup001.docx]

# Supplementary Material

*Appendix 1*

*Table 2: List of Themes*

| **Theme** | **Papers** |
| --- | --- |
| Diagnostic Reliability | Adaji A, Fortney J. 2017. Telepsychiatry in Integrated Care Settings. *Focus, The Journal of Lifelong Learning in Psychiatry.* **15:**257-263.  Amirsadri A, Burns J, Pizzuti A, Arfken CL. 2017. Home-Based Telepsychiatry in US Urban Area. *Case Reports in Psychiatry.* **2017:**6296423.  Balon R, Beresin EV, Coverdale JH, Louie AK, Roberts LW. Strengthening  telepsychiatry's role in clinical care and education. *Academic Psychiatry. 2015;*  **39**: 6-9  Bashshur RL, Shannon GW, Bashshur N, Yellowlees PM. 2016. The  Empirical Evidence for Telemedicine Interventions in Mental Disorders. *Telemedicine and e-Health.*  **22**:87-113  Bishop J.E., O’Reilly R.L., Maddox K., Hutchinson L.J. 2002. Client satisfaction in a feasibility study comparing face-to-face interviews with telepsychiatry. *Journal of telemedicine and telecare*. **8**: 217-221  Boydell KM, Hodgins M, Pignatiello A, Teshima J, Edwards H, Willis D. 2014. Using technology to deliver mental health services to children and youth: a scoping review. *Journal of the Canadian Academy of Child and Adolescent Psychiatry*. **23**:87-99.  Campbell R, O'Gorman J, Cernovsky ZZ. 2015. Reactions of Psychiatric Patients to Telepsychiatry. *Mental Illness Journal.* **7**: 6101.  Chakrabarti S. 2015. Usefulness of telepsychiatry: A critical evaluation of videoconferencing-based  approaches. *World Journal of Psychiatry.* **22:** 286-304.  Chipps J, Brysiewicz P, Mars M. 2012. Effectiveness and feasibility of telepsychiatry in resource constrained environments? A systematic review of the evidence. *African Journal of Psychiatry.* **15**: 235-43.  Chipps J, Ramlall S, Madigoe T, King H, Mars M. 2012. Developing telepsychiatry services in KwaZulu-Natal -- an action research study. *African Journal of Psychiatry*. **15:** 255-63.  De Las Cuevas C, A.J, De La Fuente J., Serrano P. 2003. Telepsychiatry in the Canary Islands: user acceptance and satisfaction. *Journal of Telemedicine and Telecare.* **9**: 221-4.  Deslich S, Stec B, Tomblin S, Coustasse A. 2013. Telepsychiatry  in the 21(st) century: transforming healthcare with technology. *Perspectives in Health Information Management.* **10:** 1f.  Diamond, J.M., Bloch, R.M. 2010. Telepsychiatry assessments of child or adolescent behavior disorders: A review of evidence and issues. *Telemedicine and e-Health.* **16:** 712-716  Gammon D, Bergvik S, Bergmo T, Pedersen S.1996. Videoconferencing  in psychiatry: a survey of use in northern Norway. *Journal of Telemedicine and Telecare.* **2:** 192-198.  Elford DR, White H, St John K, Maddigan B, Ghandi M, Bowering R. 2001. A prospective satisfaction study and cost analysis of a pilot child telepsychiatry service in Newfoundland. *Journal of Telemedicine and Telecare.* **7:** 73-81.  Elford R., White H., Bowering R., Ghandi A., Maddiggan B., St John K., House M., Harnett J., West R., Battcock A. 2000. A randomized, controlled trial of child psychiatric assessments conducted using videoconferencing. *Journal of telemedicine and telecare.* **6:** 73-82  Glueck DA. 2011. Telepsychiatry in private practice. *Child and Adolescent Psychiatric Clinics of North America*. **20**: 1-11.  Green AS, Ruchman SG, Katz CL, Singer EK. 2020. Piloting forensic tele-mental health evaluations of asylum seekers. *Psychiatry Research*. **291**: 113256.  Gunter, T.D, Srinivasaraghavan, J. Terry, N.P. 2003. Misinformed Regulation of Electronic Medicine Is Unfair to Responsible Telepsychiatry. *Journal of the American Academy of Psychiatry and the Law.* **31:** 10-14.  Grosch, M.C., Gottlieb, M.C., Cullum, C.M. 2011. Initial practice recommendations for teleneuropsychology. *The Clinical Neuropsychologist*. **25:** 1119-1133.  Hailey D, Jacobs P, Simpson J, Doze S. An  assessment framework for telemedicine applications. *Journal of telemedicine and telecare*. **5:** 162-70.  Hariman K, Ventriglio A, Bhugra D. 2019. The Future of Digital Psychiatry. *Current Psychiatry Reports.* **13:** 88.  Hilt, R.J. 2017. Telemedicine for child collaborative or integrated care. *Child and Adolescent Psychiatric Clinics of North America.* **26:** 637-645.  Hilty DM Johnston, B McCarron, R.M. 2016. How e-Mental health adds to traditional outpatient and newer models of integrated care for patients, providers, and systems. Mucic, Davor [Ed], Hilty, Donald M [Ed]. e-Mental health. Cham, Switzerland: Springer International Publishing, Switzerland; pp. 129-149.  Hilty DM, Bourgeois, J.A., Nesbitt, T.S., Hales, R.E. 2004. Cost issues with telepsychiatry in the United States. *Psychiatric Bulletin.* **28:** 6-8.  Hilty DM, Ferrer DC, Parish MB, Johnston B, Callahan EJ, Yellowlees PM. 2013. The effectiveness of telemental health. *Journal of telemedicine and telecare.* **19:** 444-454.  Hilty DM, Luo, John S, Morache, Chris, Marcelo, Divine A & Nesbitt, Thomas S. 2002. Telepsychiatry: An overview for psychiatrists. *CNS Drugs.* **16:** 527-548.  Hilty DM, Marks, S.L., Urness, D., Yellowlees, P.M., Nesbitt, T.S.2004. Clinical and Educational Telepsychiatry Applications: A Review. *The Canadian Journal of Psychiatry / La Revue canadienne de psychiatrie*. **49:** 12-23.  Hubley S, Lynch SB, Schneck C, Thomas M, Shore J. 2016. Review  of key telepsychiatry outcomes. *World Journal of Psychiatry*. **22:** 269-82  Jacob MK, Larson JC, Craighead WE. 2012. Establishing a telepsychiatry consultation practice in rural Georgia for primary care  physicians: a feasibility report. *Clinical Paediatrics.* **51:** 1041-1047.  Jones, B.N. 2002. Suicide among the elderly: The promise of telecommunications. *The British Journal of Psychiatry*. **181:** 191-192.  Kaftarian E. Lessons Learned in Prison and Jail-Based Telepsychiatry. *Current Psychiatric Reports*. **21:** 15.  Khalifa, N, Saleem, Y., Stankard, P. 2008. The use of telepsychiatry within forensic practice: A literature review on the use of videolink. *Journal of Forensic Psychiatry & Psychology.* **19:** 2-13.  Lau, M.E., Way, B.B., Fremont, W.P. 2011. Assessment of SUNY Upstate Medical University's child telepsychiatry consultation program. I*nternational Journal of Psychiatry in Medicine*. **42:** 93-104  Lee A, S.N., O'Connell F, Dyer A, Boniface K, Betz J. 2015. Telepsychiatric assessment of a mariner expressing suicidal ideation. *International Maritime Health.* **66**: 49-51.  Leonard S. 2004. The successes and challenges of developing a prison telepsychiatry service. *Journal of Telemedicine and Telecare*. **10**: 69-71.  Lexcen, F.J., Hawk, G.L., Herrick, S., Blank, M.B. 2006. Use of Video Conferencing for Psychiatric and Forensic Evaluations. *Psychiatric Services.* **57:** 713-715.  Litwack, S.D., Jackson, C.E., Chen, M., Sloan, D.M., Hatgis, C., Litz, B.T. et al. 2014. Validation of the use of video teleconferencing technology in the assessment of PTSD*. Psychological Services.* **11:** 290-294.  Loh, P. K, Maher, S., Goldswain, P., Flicker, L., Ramesh, P., Saligari, J. 2005. Diagnostic accuracy of telehealth community dementia assessments. *Journal of the American Geriatrics Society.* **53:** 2043-2044  Loh, P.K., Donaldson, M., Flicker, L., Maher, Sean S., Goldswain, P. 2007. Development of a telemedicine protocol for the diagnosis of Alzheimer's disease. *Journal of Telemedicine and Telecare.* **13:** 90-94.  Malhotra S, Chakrabarti S, Shah R. 2013. Telepsychiatry:Promise, potential, and challenges. *Indian Journal of Psychiatry.* **55**: 3-11.  Martin-Khan, M., Wootton, R., Whited, J., Gray, L.C. 2011. A systematic review of studies concerning observer agreement during medical specialist diagnosis using videoconferencing. *Journal of Telemedicine and Telecare*. **17:** 350-357.  Mazhari S, Ghaffari Nejad A, Mofakhami O, Raaii F, Bahaadinbeigy K. Evaluating  the Diagnostic Agreement between Telepsychiatry Assessment and Face-to-Face  Visit: A Preliminary Study. *Iranian Journal of Psychiatry*. 2019; **14:** 236-241.  Menon, A. Srikumar, K., Prasad, K., Popuri, Chrismer, J.B., Raskin, A., Hebel, J.R. et al. 2001. Evaluation of a portable low cost videophone system in the assessment of depressive symptoms and cognitive function in elderly medically ill veterans. *Journal of Nervous and Mental Disease*. **189:** 399-401.  Heravian A, Chang BP. 2018. Mental health and telemedicine in the acute care setting: Applications of telepsychiatry in the ED.*The American Journal of Emergency Medicine.* **36:** 1118-1119  Matsuura S, Hosaka T, Yukiyama T, Ogushi Y, Okada Y, Haruki Y, Nakamura M. 2000. Application of telepsychiatry: a preliminary study. *Psychiatry and Clinical Neurosciences.* **54:** 55-58.  Brodey BB, Claypoole KH, Motto J, Arias RG, Goss R. 2000. Satisfaction of forensic psychiatric patients with remote telepsychiatric evaluation. *Psychiatric Services.* **51:** 1305-1307.  Jones BN 3rd, Johnston D, Reboussin B, McCall WV. 2001. Reliability of telepsychiatry assessments: subjective versus observational ratings. *Journal of Geriatric Neurology and Psychiatry.* **14:** 66-71.  Rohland BM. 2001. Telepsychiatry in the heartland: if we build it, will they come?. *Community Mental Health Journal.* **37:** 449-459.  Shore JH, Savin D, Orton H, Beals J, Manson SM. 2007. Diagnostic reliability of telepsychiatry in American Indian veterans. *American Journal of Psychiatry.* **164:** 115-1118.  Singh SP, Arya D, Peters T. 2007. Accuracy of telepsychiatric assessment of new routine outpatient referrals. *BMC Psychiatry.* **7**: 55.  Myers KM, Palmer NB, Geyer JR. 2011. Research in child and adolescent telemental health. *Child and Adolescent Psychiatric Clinics in North America.* **20:** 155-171.  Savin D, Glueck DA, Chardavoyne J, Yager J, Novins DK. 2011. Bridging cultures: child psychiatry via videoconferencing. *Child and Adolescent Psychiatric Clinics in North America.* **20:** 125-134.  Myers K. Telepsychiatry: time to connect. *Journal of the American Academy of Child and Adolescent Psychiatry. 2013;* **52:** 217-219.  Nassan M, Frye MA, Adi A, Alarcón RD. 2015. Telepsychiatry for post-traumatic stress disorder: a call for action in the Syrian conflict. *Lancet Psychiatry*. **2:** 866.  Das S, Manjunatha N, Kumar CN, Math SB, Thirthalli J. 2020. Tele-psychiatric after care clinic for the continuity of care: A pilot study from an academic hospital. *Asian Journal of Psychiatry.* **48:** 101886.  Sharma A, Sasser T, Schoenfelder Gonzalez E, Vander Stoep A, Myers K.2020. Implementation of Home-Based Telemental Health in a Large Child Psychiatry Department During the COVID-19 Crisis. *Journal of Child and Adolescent Psychopharmacology.*  Frueh BC, Deitsch SE, Santos AB, Gold PB, Johnson MR, Meisler N, Magruder KM, Ballenger JC. 2000. Procedural and methodological issues in telepsychiatry research and program development. *Psychiatric Services.* **51:** 1522-1527.  Jones BN 3rd, Ruskin PE. 2001. Telemedicine and geriatric psychiatry: directions for future research and policy. *Journal of Geriatric Psychiatry and Neurology.* **14:** 59-62.  Monnier J, Knapp RG, Frueh BC. 2003. Recent advances in telepsychiatry: an updated review. *Psychiatric Services.* **54:** 1604-1609.  Modai I, Jabarin M, Kurs R, Barak P, Hanan I, Kitain L. 2007. Cost effectiveness, safety, and satisfaction with video telepsychiatry versus face-to-face care in ambulatory settings. *Telemedicine and e-Health*. **12:** 515-520.  Ramos-Ríos R, Mateos R, Lojo D, Conn DK, Patterson T. 2012. Telepsychogeriatrics: a new horizon in the care of mental health problems in the elderly. *International Psychogeriatrics.* **24:** 1708-1724.  Yellowlees P, Richard Chan S, Burke Parish M. 2015. The hybrid doctor-patient relationship in the age of technology - Telepsychiatry consultations and the use of virtual space. *International Review Psychiatry.* **27:** 476-489.  Fortney JC, Pyne JM, Turner EE, Farris KM, Normoyle TM, Avery MD, Hilty DM, Unützer J. 2015. Telepsychiatry integration of mental health services into rural primary care settings. *International Review Psychiatry.* **27:** 525-539.  Roth DE, Ramtekkar U, Zeković-Roth S. 2019. Telepsychiatry: A New Treatment Venue for Pediatric Depression. *Child and Adolescent Psychiatric Clinics in North America.* **28:** 377-395.  Farabee D., Calhoun S., Veliz R. 2016. An experimental comparison of telepsychiatry and conventional psychiatry for parolees. *Psychiatric services.* **67**: 562-565.  O’Reilly R., Bishop J., Maddox K., Hutchinson L., Fisman M., Takhar J. 2007. Is telepsychiatry equivalent to face-to-face psychiatry? Results from a randomized controlled equivalence trial. *Psychiatric Services*. **58:** 863-843  Fishkind, Avrim B & Cuyler, Robert N. 2013. The role of telepsychiatry. Zun, Leslie S [Ed], Chepenik, Lara G [Ed], Mallory, Mary Nan S [Ed]. Behavioral emergencies for the emergency physician. New York, NY, US: Cambridge University Press, US; pp. 303-307.  Alessi N. 2002. High-bandwidth interactive telepsychiatry. *Psychiatric Services.* **53:** 901-902.  Jones BN 3rd. 2001. Telepsychiatry and geriatric care. *Current Psychiatry Reports.* **3:** 29-36.  Zaylor C. 1999. Clinical outcomes in telepsychiatry. *Journal of Telemedicine and Telecare.* **5:** S59-60.  Zaylor C, Nelson EL, Cook DJ. 2001. Clinical outcomes in a prison telepsychiatry clinic. *Journal of Telemedicine and Telecare.* **1:** 47-49.  Yellowlees, Peter M, Hilty, Donald M, Marks, Shayna L, Neufeld, Jonathan & Bourgeois, James A. 2008. A retrospective analysis of a child and adolescent eMental Health program. *Journal of the American Academy of Child & Adolescent Psychiatry.* **47:** 103-107.  Work Group on Quality Issues (WGQI) & American Academy of Child and Adolescent Psychiatry (AACAP), US. 2008. Practice parameter for telepsychiatry with children and adolescents. *Journal of the American Academy of Child & Adolescent Psychiatry.* **47:** 1468-1483.  Volicer L. 2015. Nursing home telepsychiatry. *Journal of the American Medical Directors Association*. **16**: 7-8.  van Wynsberghe A, Gastmans C. 2009. Telepsychiatry and the meaning of in-person contact: a preliminary ethical appraisal. *Medicine, Health Care and Philosophy.* **12:** 469-476.  Sunjaya AP, Chris A, Novianti D. 2020. Efficacy,  patient-doctor relationship, costs and benefits of utilizing telepsychiatry for  the management of post-traumatic stress disorder (PTSD): a systematic  review. *Trends in Psychiatry and Psychotherapy.* **42:** 102-110.  Starling, J., Dossetor, D. 2005. Child and Adolescent Telepsychiatry. Wootton, Richard [Ed], Batch, Jennifer [Ed]. Telepediatrics: Telemedicine and child health. Lo: Royal Society of Medicine Press; pp. 77-87.  Munro C.C., Hynan L.S, Grosch M, Parikh M, Weiner MF. 2014. Teleneuropsychology:  evidence for video teleconference-based neuropsychological assessment. *Journal of the International Neuropsychological Society.* **20:** 1028-33.  Nelson EL, Zaylor C, Cook D. 2004. A comparison of psychiatrist evaluation and patient symptom report in a jail telepsychiatry  clinic. *Telemedicine and e-Health.* **2:**S54-59  Saeed SA, Anand V. 2015. Use of Telepsychiatry in Psychodynamic Psychiatry. *Psychodynamic Psychiatry*. **43**: 569-83.  Setterberg, S.R., Busseri, M.A., Fleissner, R.M., Kenney, E.M., Flom, J.A., Fischer, K.J. Remote assessment of the use of seclusion and restraint with paediatric psychiatric patients. *Journal of Telemedicine and Telecare. 2003;*  **9:** 176-179  Shores, M.M., Ryan-Dykes, P., Williams, R.M., Mamerto, B., Sadak, T., Pascualy, M. et al. 2004. Identifying undiagnosed dementia in residential care veterans: comparing telemedicine to in-person clinical examination. *International Journal of Geriatric Psychiatry.* **19:** 101-108 |
| Outcomes | Aboujaoude, E., Gega. 2020. From digital mental health interventions to digital "addiction": Where the two fields converge. *Frontiers in Psychiatry.* 10.  Abrams J, Sossong S, Schwamm LH, Barsanti L, Carter M, Kling N, Kotarski M, Leddy J, Meller B, Simoni M, Sullivan M, Wozniak J. 2017. Practical Issues in Delivery of Clinician-to-Patient Telemental Health in an Academic Medical Center. *Harvard Review of Psychiatry.* **25**:135-145.  Adaji A, Fortney J. 2017. Telepsychiatry in Integrated Care Settings. *Focus, The Journal of Lifelong Learning in Psychiatry.* **15:**257-263.  American Academy of Child and Adolescent Psychiatry (AACAP) Committee on Telepsychiatry and AACAP Committee on Quality Issues. 2017. Clinical update: Telepsychiatry with children and adolescents. *Journal of the American Academy of Child & Adolescent Psychiatry.* **56**: 875-893  Barrera-Valencia C, Benito-Devia AV, Vélez-Álvarez C, Figueroa-Barrera M, Franco-Idárraga SM. 2017. Cost-effectiveness  of Synchronous vs. Asynchronous Telepsychiatry in Prison Inmates With  Depression. *Rev Colomb Psiquiatr*. **46**: 65-73.  Bashshur RL, Shannon GW, Bashshur N, Yellowlees PM. 2016. The  Empirical Evidence for Telemedicine Interventions in Mental Disorders. *Telemedicine and e-Health.*  **22**: 87-113  Batastini, Ashley B, McDonald, Brendan R & Morgan, Robert D. 2013. Videoteleconferencing in forensic and correctional practice. Myers, Kathleen [Ed], Turvey, Carolyn L [Ed]. Telemental health: Clinical, technical, and administrative foundations for evidence-based practice. Amsterdam, Netherlands: Elsevier, Netherlands; pp. 251-271.  Behere PB, Mansharamani HD, Kumar K. 2017. Telepsychiatry: Reaching the unreached. *Indian Journal of Medical Research.* **146:** 150-152.  Bishop J.E., O’Reilly R.L., Maddox K., Hutchinson L.J. 2002. Client satisfaction in a feasibility study comparing face-to-face interviews with telepsychiatry. *Journal of telemedicine and telecare*. **8**: 217-221  Bahloul, H.J., Mani, N. 2013. International telepsychiatry: A review of what has been published. *Journal of Telemedicine and Telecare.* **19**: 293-294.  Bolle R.R., Trondsen M.V., Stensland G.Ø., Tjora A. 2018. Usefulness of  videoconferencing in psychiatric emergencies -- a qualitative study. *Health and Technology.*  **8:** 111-117.  Borders C.B. 2017. Realizing the Promises of Telepsychiatry in Special Populations. *Mental Illness Journal.* **9:** 7135.  Boydell KM, Hodgins M, Pignatiello A, Teshima J, Edwards H, Willis D. 2014. Using technology to deliver mental health services to children and youth: a scoping review. *Journal of the Canadian Academy of Child and Adolescent Psychiatry*. **23**:87-99.  Buist A, Coman G, Silvas A, Burrows G. 2000. An evaluation of the telepsychiatry programme in Victoria, Australia. *Journal of Telemedicine and Telecare.* **6**: 216-21.  Butterfield, A. 2018. Telepsychiatric evaluation and consultation in emergency care settings. *Child and Adolescent Psychiatric Clinics of North America.* **27:** 467-478.  Chakrabarti S. 2015. Usefulness of telepsychiatry: A critical evaluation of videoconferencing-based  approaches. *World Journal of Psychiatry.* **22:** 286-304.  Chan S, Parish M, Yellowlees P. 2015. Telepsychiatry Today.*Current Psychiatry Reports.* **17:** :89  Chan SR, Torous J, Hinton L, Yellowlees P. 2014. Mobile Tele-Mental Health: Increasing Applications and a Move to Hybrid Models of Care. *Healthcare (Basel)*. **6**: 220-33.  Cheng KM, Siu BW, Au Yeung CC, Chiang TP, So MH, Yeung MC. 2018. Telepsychiatry for stable Chinese psychiatric out-patients in custody in Hong Kong: a case-control pilot study. *Hong Kong Medical Journal.* **24:** 378-383.  Chipps J, Brysiewicz P, Mars M. 2012. Effectiveness and feasibility of telepsychiatry in resource constrained environments? A systematic review of the evidence. *African Journal of Psychiatry.* **15**: 235-43.  Caudill, R.L., Sager, Z. 2015. Institutionally based videoconferencing. *International Review of Psychiatry*. **27:** 496-503.  Chipps J, Ramlall S, Madigoe T, King H, Mars M. 2012. Developing telepsychiatry services in KwaZulu-Natal -- an action research study. *African Journal of Psychiatry*. **15:** 255-63.  Chong, J., Moreno, F. 2012. Feasibility and acceptability of clinic-based telepsychiatry for low-income Hispanic primary care patients. *Telemedicine and e-Health.* **18**: 297-304  American Academy of Child and Adolescent Psychiatry (AACAP) Committee on Telepsychiatry and AACAP Committee on Quality Issues. 2017. Clinical Update: Telepsychiatry With Children and Adolescents. *Journal of the American Academy of Child & Adolescent Psychiatry.* **56:** 875-893  Cowan KE, McKean AJ, Gentry MT, Hilty DM. 2019. Barriers to Use of Telepsychiatry: Clinicians as Gatekeepers. *Mayo Clinic Proceedings.* **94:** 2510-2523.  Crowe T, Jani S, Jani S, Jani N, Jani R. 2016. A pilot program in rural telepsychiatry for deaf and hard of hearing  populations. *Heliyon*. **2**: e00077  Deslich S, Stec B, Tomblin S, Coustasse A. Telepsychiatry in the 21(st) century: transforming healthcare with technology. *Perspectives in Health Information Management. 2013;* **10:** 1f.  Deslich SA, Thistlethwaite T, Coustasse A. 2013. Telepsychiatry  in correctional facilities: using technology to improve access and decrease  costs of mental health care in underserved populations. *The Permanente Journal* **17:** 80-86.  Detweiler MB, Arif S, Candelario J, Altman J, Murphy PF, Halling MH, Detweiler JG, Vasudeva S. 2011. A telepsychiatry transition clinic: the first 12 months  experience. *Journal of Telemedicine and Telecare.* **17:** 293-297.  Diamond, J.M., Bloch, R.M. 2010. Telepsychiatry assessments of child or adolescent behavior disorders: A review of evidence and issues. *Telemedicine and e-Health.* **16:** 712-716  Donley, E., McClaren, A., Jones, R., Katz, P., Goh, J. 2017. Evaluation and implementation of a telepsychiatry trial in the emergency department of a metropolitan public hospital. *Journal of Technology in Human Services*. **35:** 292-313.  Dossetor, D. R, Nunn, K. P, Fairley, M., Eggleton, D. 1999. A child and adolescent psychiatric outreach service for rural New South Wales: A telemedicine pilot study. *Journal of Paediatrics and Child Health*. **35:** 525-529.  Egede LE, Frueh CB, Richardson LK, Acierno R, Mauldin PD, Knapp RG, Lejuez C. 2009. Rationale and design: telepsychology service delivery for depressed elderly veterans. *Trials.* **10:** 22  Ellington, E., Repique, R., John R. 2013. Telemental health adoption can change psychiatric-mental health nursing practice. *Journal of the American Psychiatric Nurses Association.* **19:** 222-224  Flaum MA. 2017. When Will Telepsychiatry Reach Its "Tipping Point"?. *Psychiatric Services*. **68**:1205.  Freudenberg N, Yellowlees PM. 2014. Telepsychiatry as Part of a Comprehensive Care Plan. *Virtual Mentor*. **16**: 964-8.  Gardner JS, Plaven BE, Yellowlees P, Shore JH. 2020. Remote Telepsychiatry Workforce: a Solution to Psychiatry's Workforce Issues. *Current Psychiatric Reports.* **22:** 8.  Doze S, Simpson J, Hailey D, Jacobs P. 1999. Evaluation of a telepsychiatry pilot project. *Journal of Telemedicine and Telecare.* **5:** 38-46  Elford DR, White H, St John K, Maddigan B, Ghandi M, Bowering R. 2001. A prospective satisfaction study and cost analysis of a pilot child telepsychiatry service in Newfoundland. *Journal of Telemedicine and Telecare.* **7:** 73-81.  Elford R., White H., Bowering R., Ghandi A., Maddiggan B., St John K., House M., Harnett J., West R., Battcock A. 2000. A randomized, controlled trial of child psychiatric assessments conducted using videoconferencing. *Journal of telemedicine and telecare.* **6:** 73-82  Gentile, J.P, Cowan, A.E., Harper, B., Mast, R., Merrill, B. 2018. Reaching rural Ohio with intellectual disability psychiatry. *Journal of Telemedicine and Telecare.* **24**: 434-439  Gloff, N.E, LeNoue, S.R, Novins, D.K., Myers, K. 2015. Telemental health for children and adolescents. *International Review of Psychiatry*. **27:** 513-524.  Glueck DA. 2011. Telepsychiatry in private practice. *Child and Adolescent Psychiatric Clinics of North America*. **20**: 1-11.  Gopalan P, Shenai N, Dunn S, Bilderback A. 2020. Healthcare  utilization in patients pre-and post-telepsychiatry consultation compared to  in-person consultation-liaison sites. *General Hospital Psychiatry.* **67:** 154-155.  Gowda, Guru S, Kulkarni, Karishma, Bagewadi, Virupaksha, R. P. S., Shyam, Manjunatha, B. R, Shashidhara, Harihara N, et al. 2018. A study on collaborative telepsychiatric consultations to outpatients of district hospitals of Karnataka, India. *Asian Journal of Psychiatry.* **37:** 161-166  Grady B. 2012. Promises and limitations of telepsychiatry in rural adult mental health care. *World Psychiatry.* **11**: 199-201.  Haghnia Y., Samad-Soltani T., Yousefi M., Sadr H., Rezaei-Hachesu P. 2010. Telepsychiatry-based care for the treatment follow-up of Iranian war veterans with post-traumatic stress disorder: a randomized controlled trial. *Iranian journal of medical sciences*. **44**: 291-298.  Hariman K, Ventriglio A, Bhugra D. 2019. The Future of Digital Psychiatry. *Current Psychiatry Reports.* **13:** 88.  Hensel J, Graham R, Isaak C, Ahmed N, Sareen J, Bolton J. A Novel Emergency Telepsychiatry Program in a Canadian Urban Setting: Identifying and Addressing Perceived Barriers for Successful Implementation: Un nouveau programme de télépsychiatrie d'urgence en milieu urbain canadien: Identifier et aborder les obstacles perçus d'une mise en œuvre réussie. *Canadian Journal of Psychiatry; 2020* **65:** 559-567.  Hilt, R.J. 2017. Telemedicine for child collaborative or integrated care. *Child and Adolescent Psychiatric Clinics of North America.* **26:** 637-645.  Hilty DM Johnston, B McCarron, R.M. 2016. How e-Mental health adds to traditional outpatient and newer models of integrated care for patients, providers, and systems. Mucic, Davor [Ed], Hilty, Donald M [Ed]. e-Mental health. Cham, Switzerland: Springer International Publishing, Switzerland; pp. 129-149.  Hilty DM, Mucic, D. 2016. Technology, health, and contemporary practice: How does e-mental health fit it and what does it offer? Mucic, Davor [Ed], Hilty, Donald M [Ed]. e-Mental health. Cham, Switzerland: Springer International Publishing, Switzerland; pp. 3-27  Hilty DM, Bourgeois, J.A., Nesbitt, T.S., Hales, R.E. 2004. Cost issues with telepsychiatry in the United States. *Psychiatric Bulletin.* **28:** 6-8.  Hilty DM, Cobb, H.C., Neufeld, J.D., Bourgeois, J.A., Yellowlees, P.M. 2008. Telepsychiatry reduces geographic physician disparity in rural settings, but is it financially feasible because of reimbursement? *Psychiatric Clinics of North America*. **31:** 85-94  Hilty DM, Crawford A, Teshima J, Chan S, Sunderji N, Yellowlees PM, Kramer G, O'neill P, Fore C, Luo J, Li ST. 2015. A framework  for telepsychiatric training and e-health: Competency-based education, evaluation and implications. *International Review of Psychiatry.* **27:** 569-592.  Hilty DM, Ferrer DC, Parish MB, Johnston B, Callahan EJ, Yellowlees PM. 2013. The effectiveness of telemental health. *Journal of telemedicine and telecare.* **19:** 444-454.  Hilty DM, Luo, John S, Morache, Chris, Marcelo, Divine A & Nesbitt, Thomas S. 2002. Telepsychiatry: An overview for psychiatrists. *CNS Drugs.* **16:** 527-548.  Hilty DM, Marks, S.L., Urness, D., Yellowlees, P.M., Nesbitt, T.S.2004. Clinical and Educational Telepsychiatry Applications: A Review. *The Canadian Journal of Psychiatry / La Revue canadienne de psychiatrie*. **49:** 12-23.  Hilty DM, Sunderji, N., Suo, S., Chan, S., McCarron, R.M. 2018. Telepsychiatry and other technologies for integrated care: Evidence base, best practice models and competencies. *International Review of Psychiatry.* **30**: 292-309.  Hilty DM, Yellowlees PM, Parrish MB, Chan S. 2015. Telepsychiatry: Effective, Evidence-Based, and at a Tipping Point in Health Care Delivery?. *Psychiatric Clinics of North America.* **38**: 559-92.  Hubley S, Lynch SB, Schneck C, Thomas M, Shore J. 2016. Review  of key telepsychiatry outcomes. *World Journal of Psychiatry*. **22:** 269-82  Hungerbuehler I, Valiengo L, Loch AA, Rössler W, Gattaz WF. 2016. Home-Based  Psychiatric Outpatient Care Through Videoconferencing for Depression: A  Randomized Controlled Follow-Up Trial. *Journal of Medical Internet Research.* **3:** e36.  Jacob MK, Larson JC, Craighead WE. 2012. Establishing a telepsychiatry consultation practice in rural Georgia for primary care  physicians: a feasibility report. *Clinical Paediatrics.* **51:** 1041-1047.  Jefee B.H., Mani N. International telepsychiatry: a review of what has been  published. *Journal of Telemedicine and Telecare*. **19:** 293-4.  Jefee-Bahloul H. Telemental health in the middle East: overcoming the barriers. *Frontiers in Public Health*. 2014; **2:** 86  Kaftarian E. Lessons Learned in Prison and Jail-Based Telepsychiatry. *Current Psychiatric Reports*. **21:** 15.  Keilman, P. 2005. Telepsychiatry with Child Welfare Families Referred to a Family Service Agency. *Telemedicine and e-Health.* **11:** 98-101.  Kennedy C, Yellowlees P. 2000. A community-based approach to evaluation of health outcomes and costs for telepsychiatry in a rural population: preliminary results. *Journal of Telemedicine and Telecare*. **6:** S155-7.  Khalifa, N, Saleem, Y., Stankard, P. 2008. The use of telepsychiatry within forensic practice: A literature review on the use of videolink. *Journal of Forensic Psychiatry & Psychology.* **19:** 2-13.  Koblauch, H., Reinhardt, S.M., Lissau, W.J. & Jensen, P. 2018. The effect of telepsychiatric modalities on reduction of readmissions in psychiatric settings: A systematic review. *Journal of Telemedicine and Telecare.* **24:**, 31-36.  Kornbluh RA. Staying true to the mission: adapting telepsychiatry to a new environment. *CNS Spectrums*. 2014; **19**: 482-3.  Krzystanek M, Krysta K, Skałacka K. 2017. Treatment Compliance in the Long-Term Paranoid Schizophrenia Telemedicine Study. *Journal of Technology in Behavioural Science*. **2**: 84-87.  Krzystanek, M., Krzeszowski, D., Jagoda, K., Krysta, K. 2015. Long term telemedicine study of compliance in paranoid schizophrenia. *Psychiatria Danubina.* **27:** S266-S268.  LaBelle B, Franklyn AM, Pkh Nguyen V, Anderson KE, Eibl JK, Marsh DC. 2018. Characterizing the Use of Telepsychiatry for Patients with Opioid Use Disorder and Cooccurring Mental Health Disorders in Ontario, Canada. *International Journal of Telemedicine and Applications.* **2018:** 7937610.  Lal, S., Abdel-Baki, A., Sujanani, S., Bourbeau, F., Sahed, I., Whitehead, J. 2020. Perspectives of young adults on receiving telepsychiatry services in an urban early intervention program for first-episode psychosis: A cross-sectional, descriptive survey study. *Frontiers in Psychiatry*. 11  Lau, M.E., Way, B.B., Fremont, W.P. 2011. Assessment of SUNY Upstate Medical University's child telepsychiatry consultation program. I*nternational Journal of Psychiatry in Medicine*. **42:** 93-104  Madhavan G. 2019. Telepsychiatry in intellectual disability psychiatry: literature  review. *BJPsych Bulletin.* **43**: 167-173  Mahmoud H, Vogt EL, Dahdouh R, Raymond ML. 2020. Using Continuous Quality Improvement to Design and Implement a Telepsychiatry Program in Rural Illinois. *Psychiatric Services.* **15**: appips201900231.  Malhotra S, Chakrabarti S, Shah R. 2013. Telepsychiatry:Promise, potential, and challenges. *Indian Journal of Psychiatry.* **55**: 3-11.  Malhotra, S., Shah, R. 2018. Telepsychiatry and digital mental health care in child and adolescent psychiatry: Implications for service delivery in low- and middle-income countries. Hodes, Matthew [Ed], Shur-Fen Gau, Susan [Ed], De Vries, Petrus J [Ed]. Understanding uniqueness and diversity in child and adolescent mental health. San Diego, CA, US: Elsevier Academic Press, US; pp. 263-287  Math SB, Moirangthem S, Kumar NC. 2015. Tele-Psychiatry: After Mars, Can we Reach the Unreached?. *Indian Journal of Psychological Medicine.* **37:** 120-1.  Mazhari S, Ghaffari Nejad A, Mofakhami O, Raaii F, Bahaadinbeigy K. 2019. Evaluating  the Diagnostic Agreement between Telepsychiatry Assessment and Face-to-Face  Visit: A Preliminary Study. *Iranian Journal of Psychiatry*. **14:** 236-241.  McGrath J. ADHD and  Covid-19: Current roadblocks and future opportunities. *The Irish Journal of Psychological Medicine. 2020.* **21:** 1-22.  McLaren P. 2004. Telepsychiatry in Europe. *International Psychiatry.* **1**: 8-10.  Mettner J. The doctor is in another town: telepsychiatry brings care to people in rural Minnesota. *Minnesota Medicine*. 2013; **96**: 22-5.  Mielonen M, Ohinmaa A., Moring J & Isohanni M. Videoconferencing in telepsychiatry. *Journal of Technology in Human Services.* 2002; **20:** 183-199.  Clarke CS. 2018. Telepsychiatry in Asperger's syndrome. *Irish Journal of Psychological Medicine.* **35:** 325-328.  Ikelheimer, D.M. 2008. Treatment of opioid dependence via home- based telepsychiatry. *Psychiatric Services.* **59:** 1218-1219.  Benyakorn S. 2016. Implementing Telepsychiatry in Thailand Benefits and Challenges. *Journal of the Medical Association of Thailand*. **99**: S260-S266.  Hilty DM., Yellowlees, P.M. 2015. Collaborative mental health services using multiple technologies: The new way to practice and a new standard of practice? *Journal of the American Academy of Child & Adolescent Psychiatry.* **54:** 245-246.  Gabel S. 2009. Telepsychiatry, public mental health, and the workforce shortage in child and adolescent psychiatry. *Journal of the American Academy of Child & Adolescent Psychiatry.* **48:** 1127-1128.  Whitten P, Kuwahara E. 2004. A multi-phase telepsychiatry programme in Michigan: organizational factors affecting utilization and user perceptions. *Journal of Telemedicine and Telecare.* **10:** 254-261.  Narasimhan M, Druss BG, Hockenberry JM, Royer J, Weiss P, Glick G, Marcus SC, Magill J. 2015. Impact of a Telepsychiatry Program at Emergency Departments Statewide on the Quality, Utilization, and Costs of Mental Health Services. *Psychiatric Services.* **66:** 1167-72  Brodey BB, Claypoole KH, Motto J, Arias RG, Goss R. 2000. Satisfaction of forensic psychiatric patients with remote telepsychiatric evaluation. *Psychiatric Services.* **51:** 1305-1307.  Rohland BM. 2001. Telepsychiatry in the heartland: if we build it, will they come?. *Community Mental Health Journal.* **37:** 449-459.  Singh SP, Arya D, Peters T. 2007. Accuracy of telepsychiatric assessment of new routine outpatient referrals. *BMC Psychiatry.* **7**: 55.  Fox KC, Connor P, McCullers E, Waters T. 2008. Effect of a behavioural health and specialty care telemedicine programme on goal attainment for youths in juvenile detention. *Journal of Telemedicine and Telecare.* **14:** 227-230.  Myers KM, Palmer NB, Geyer JR. 2011. Research in child and adolescent telemental health. *Child and Adolescent Psychiatric Clinics in North America.* **20:** 155-171.  Szeftel R, Federico C, Hakak R, Szeftel Z, Jacobson M. 2012. Improved access to mental health evaluation for patients with developmental disabilities using telepsychiatry. *Journal of Telemedicine and Telecare.* **18:** 317-321.  Ulzen T, Williamson L, Foster PP, Parris-Barnes K. 2013. The evolution of a community-based telepsychiatry program in rural Alabama: lessons learned-a brief report. *Community Mental Health Journal.* **49:** 101-5.  Myers K. 2013. Telepsychiatry: time to connect. *Journal of the American Academy of Child and Adolescent Psychiatry.* **52:** 217-219.  Sharma A, Sasser T, Schoenfelder Gonzalez E, Vander Stoep A, Myers K.2020. Implementation of Home-Based Telemental Health in a Large Child Psychiatry Department During the COVID-19 Crisis. *Journal of Child and Adolescent Psychopharmacology.*  Frueh BC, Deitsch SE, Santos AB, Gold PB, Johnson MR, Meisler N, Magruder KM, Ballenger JC. 2000. Procedural and methodological issues in telepsychiatry research and program development. *Psychiatric Services.* **51:** 1522-1527.  Monnier J, Knapp RG, Frueh BC. 2003. Recent advances in telepsychiatry: an updated review. *Psychiatric Services.* **54:** 1604-1609.  McGinty KL, Saeed SA, Simmons SC, Yildirim Y. 2006. Telepsychiatry and e-mental health services: potential for improving access to mental health care. *Psychiatric Quarterly.* **77:** 335-342.  Urness D, Wass M, Gordon A, Tian E, Bulger T. 2006. Client acceptability and quality of life--telepsychiatry compared to in-person consultation. *Journal of Telemedicine and Telecare.* **12:** 251-254.  Modai I, Jabarin M, Kurs R, Barak P, Hanan I, Kitain L. 2007. Cost effectiveness, safety, and satisfaction with video telepsychiatry versus face-to-face care in ambulatory settings. *Telemedicine and e-Health*. **12:** 515-520.  Yellowlees P, Burke MM, Marks SL, Hilty DM, Shore JH. 2008. Emergency telepsychiatry. *Journal of Telemedicine and Telecare.* **14:** 277-281.  García-Lizana F, Muñoz-Mayorga I. 2010. What about telepsychiatry? A systematic review.*The Primary Care Companion to the Journal of Clinical Psychiatry.* **12.**  Myers KM, Vander Stoep A, McCarty CA, Klein JB, Palmer NB, Geyer JR, Melzer SM. 2010. Child and adolescent telepsychiatry: variations in utilization, referral patterns and practice trends. *Journal of Telemedicine and Telecare.* **16:** 128-133.  Szeftel R, Mandelbaum S, Sulman-Smith H, Naqvi S, Lawrence L, Szeftel Z, Coleman S, Gross L. 2011. Telepsychiatry for children with developmental disabilities: applications for patient care and medical education. *Child and Adolescent Psychiatric Clinics in North America.* **20:** 95-111.  Grady BJ, Lever N, Cunningham D, Stephan S. 2011. Telepsychiatry and school mental health. *Child and Adolescent Psychiatric Clinics in North America.* **20:** 81-94.  Shim R, Ye J, Yun K. 2012. Treating culturally and linguistically isolated Koreans via telepsychiatry. *Psychiatric Services.* **63:** 946.  Ramos-Ríos R, Mateos R, Lojo D, Conn DK, Patterson T. 2012. Telepsychogeriatrics: a new horizon in the care of mental health problems in the elderly. *International Psychogeriatrics.* **24:** 1708-1724.  Shore JH. 2013. Telepsychiatry: videoconferencing in the delivery of psychiatric care. *American Journal of Psychiatry.* **170:** 256-262.  Salmoiraghi A, Hussain S. 2015. A Systematic Review of the Use of Telepsychiatry in Acute Settings. *Journal of Psychiatric Practice*. **21:** 389-393.  Shore J. 2015. The evolution and history of telepsychiatry and its impact on psychiatric care: Current implications for psychiatrists and psychiatric organizations. *International Review of Psychiatry.* **27:** 469-475.  Yellowlees P, Richard Chan S, Burke Parish M. 2015. The hybrid doctor-patient relationship in the age of technology - Telepsychiatry consultations and the use of virtual space. *International Review Psychiatry.* **27:** 476-489.  Fortney JC, Pyne JM, Turner EE, Farris KM, Normoyle TM, Avery MD, Hilty DM, Unützer J. 2015. Telepsychiatry integration of mental health services into rural primary care settings. *International Review Psychiatry.* **27:** 525-539.  Lauckner C, Whitten P. 2016. The State and Sustainability of Telepsychiatry Programs. *The Journal of Behavioural Health Services and Research.* **43:** 305-318.  Nelson EL, Cain S, Sharp S. 2017. Considerations for Conducting Telemental Health with Children and Adolescents. *Child and Adolescent Psychiatric Clinics in North America.* **26:** 77-91.  Roberts N, Hu T, Axas N, Repetti L. 2017. Child and Adolescent Emergency and Urgent Mental Health Delivery Through Telepsychiatry: 12-Month Prospective Study. *Telemedicine and e-Health.* **23:** 842-846.  Swanson CL, Trestman RL. 2018. Rural Assertive Community Treatment and Telepsychiatry. *Journal of Psychiatric Practice.* **24:** 269-273.  Hassan A, Sharif K. 2019. Efficacy of Telepsychiatry in Refugee Populations: A Systematic Review of the Evidence. *Cureus.* **11**: e3984.  Smith K, Ostinelli E, Macdonald O, Cipriani A. 2020. COVID-19 and telepsychiatry: an evidence-based guidance for clinicians. *Journal of Medical Internet Research - International Scientific Journal for Medical Research.* **10.**  Myers K., Vander Stoep A., McCarty C.A., Katon W. 2015. Effectiveness of a telehealth service model for treating attention-deficit/hyperactivity disorder: a community-based randomized controlled trial. *Journal of the American Academy of Child and Adolescent Psychiatry.* **54:** 263-274.  Shulman M., John M., Kane J.M. 2017. Home-based outpatient telepsychiatry to improve adherence with treatment appointments: a pilot study. *Psychiatric services.* **68:** 743-746.  Farabee D., Calhoun S., Veliz R. 2016. An experimental comparison of telepsychiatry and conventional psychiatry for parolees. *Psychiatric services.* **67**: 562-565.  O’Reilly R., Bishop J., Maddox K., Hutchinson L., Fisman M., Takhar J. 2007. Is telepsychiatry equivalent to face-to-face psychiatry? Results from a randomized controlled equivalence trial. *Psychiatric Services*. **58:** 863-843  Fishkind, Avrim B & Cuyler, Robert N. 2013. The role of telepsychiatry. Zun, Leslie S [Ed], Chepenik, Lara G [Ed], Mallory, Mary Nan S [Ed]. Behavioral emergencies for the emergency physician. New York, NY, US: Cambridge University Press, US; pp. 303-307.  Saurman E, Lyle D, Perkins D, Roberts R. 2014. Successful provision of emergency mental health care to rural and remote New South Wales: an evaluation of the Mental Health Emergency Care-Rural Access Program. *Australian Health Review.* **38:** 58-64.  Mahmoud H, Vogt E. 2019. Telepsychiatry: an Innovative Approach to Addressing the Opioid Crisis. *Journal of Behavioural Health Sciences and Research.* **46:** 680-685.  Jones BN 3rd. 2001. Telepsychiatry and geriatric care. *Current Psychiatry Reports.* **3:** 29-36.  Zaylor C. 1999. Clinical outcomes in telepsychiatry. *Journal of Telemedicine and Telecare.* **5:** S59-60.  Yilmaz SK, Horn BP, Fore C, Bonham CA. 2019. An economic cost analysis of an expanding, multi-state behavioural telehealth  intervention. *Journal of Telemedicine and Telecare.* **25:** 353-364.  Yellowlees, Peter M, Hilty, Donald M, Marks, Shayna L, Neufeld, Jonathan & Bourgeois, James A. 2008. A retrospective analysis of a child and adolescent eMental Health program. *Journal of the American Academy of Child & Adolescent Psychiatry.* **47:** 103-107.  Yellowlees, Peter M, Hilty, Donald M & Mucic, Davor. 2016. Global/worldwide e-mental health: International and futuristic perspectives of telepsychiatry and the future. Mucic, Davor [Ed], Hilty, Donald M [Ed]. e-Mental health. Cham, Switzerland: Springer International Publishing, Switzerland; pp. 233-249  Work Group on Quality Issues (WGQI) & American Academy of Child and Adolescent Psychiatry (AACAP), US. 2008. Practice parameter for telepsychiatry with children and adolescents. *Journal of the American Academy of Child & Adolescent Psychiatry.* **47:** 1468-1483.  Wojtuszek, Magdalena, Kachnic, Justyna, Krysta, Krzysztof & Wutke, Joanna. 2015. Telepsychiatry in Polish patients' and doctors' opinion. *Psychiatria Danubina.* **27:** S379-S382.  Wallace, Duncan & Hodges, Samantha. 2018. Telepsychiatry in the Australian Defence Force: A success story. *Australasian Psychiatry*. **26:** 105-106.  Ventriglio, Antonio, T.J., Castaldelli-Maia, J. 2017. Telepsychiatry and social psychiatry. *International Journal of Social Psychiatry.* **63:** 387-388.  Vanderpool, D. 2015. An overview of practicing high quality telepsychiatry. Dewan, Naakesh A [Ed], Luo, John S [Ed], Lorenzi, Nancy M [Ed]. Mental health practice in a digital world: A clinician's guide. Cham, Switzerland: Springer International Publishing, Switzerland; pp. 159-181  Vander S.A.,Myers K. 2013. Methodology for conducting the children's attention-deficit hyperactivity disorder telemental health treatment study in multiple underserved communities. *Clinical Trials.* **10:** 949-958.  van Wynsberghe A, Gastmans C. 2009. Telepsychiatry and the meaning of in-person contact: a preliminary ethical appraisal. *Medicine, Health Care and Philosophy.* **12:** 469-476.  Valdagno M, Goracci A, di Volo S, Fagiolini A. 2014. Telepsychiatry: new perspectives and open issues. *CNS Spectrums.* **19:** 479-481.  Trondsen MV, Bolle SR, Stensland GØ, Tjora A. 2014. Video-confidence: a qualitative exploration of videoconferencing for psychiatric emergencies. *BC Health Service Research*. **14:** 544.  Thompson D.A., Leimig R., Gower G., Winsett R.P. 2009. Assessment of depressive symptoms during post-transplant follow-up care performed via telehealth. *Telemedicine journal and e-health*. **15:** 700-706.  Thomas RK, Suleman R, Mackay M, Hayer L, Singh M, Correll CU, Dursun S. 2020. Adapting to the impact of COVID-19 on mental health: an international perspective. *Journal of Psychiatry and Neuroscience* **45**: 229-233.  Thomas JF, Novins DK, Hosokawa PW, Olson CA, Hunter D, Brent AS, Frunzi G, Libby AM. 2018. The Use of  Telepsychiatry to Provide Cost-Efficient Care During Pediatric Mental Health Emergencies. *Psychiatric Services.* **1:** 161-168.  Thiele, J.S., Doarn, C.R., Shore, J.H. 2015. Locum tenens and telepsychiatry: Trends in psychiatric care. *Telemedicine and e-Health*. **21:** 510-513.  Sunjaya AP, Chris A, Novianti D. 2020. Efficacy,  patient-doctor relationship, costs and benefits of utilizing telepsychiatry for  the management of post-traumatic stress disorder (PTSD): a systematic  review. *Trends in Psychiatry and Psychotherapy.* **42:** 102-110.  Sulzbacher, S.,, Vallin, T., Waetzig, E.Z. 2006. Telepsychiatry improves paediatric behavioural health care in rural communities. *Journal of Telemedicine and Telecare.* **12:** 285-288  Starling, J., Dossetor, D. 2005. Child and Adolescent Telepsychiatry. Wootton, Richard [Ed], Batch, Jennifer [Ed]. Telepediatrics: Telemedicine and child health. Lo: Royal Society of Medicine Press; pp. 77-87.  Stankard, P., Younus, S. 2007. Forensic telepsychiatry. *Psychiatric Bulletin*. **31:** 155.  Spaulding, R., Cain, S., Sonnenschein, K. 2011. Urban telepsychiatry: Uncommon service for a common need. *Child and Adolescent Psychiatric Clinics of North America.* **20:** 29-39  Moirangthem S, Rao S, Kumar CN, Narayana M, Raviprakash N, Math SB. Telepsychiatry as an Economically Better Model for Reaching the Unreached: A Retrospective Report from South India. *Indian Journal of Psychological Medicine. 2017.*  **39:** 271-275.  Pelton D, Wangelin B, Tuerk P. 2015. Utilizing Telehealth to Support Treatment of Acute Stress Disorder in a Theater of War:  Prolonged Exposure via Clinical Videoconferencing. *Telemedicine and e-Health.* **21:** 382-387.  Reliford A, Adebanjo B. 2019. Use of Telepsychiatry in Pediatric Emergency Room to Decrease Length of Stay for Psychiatric Patients, Improve Resident On-Call Burden, and Reduce Factors Related to Physician Burnout. *Telemedicine and e-Health.* **25**: 828-832.  Rockhill, C.M., Tse, Y.J., Fesinmeyer, M.D., Garcia, J., & Myers, K. 2016. Telepsychiatrists' medication treatment strategies in the children's attention-deficit/hyperactivity disorder telemental health treatment study. *Journal of Child and Adolescent Psychopharmacology.* **26:** 662-671  Ruskin PE, Silver-Aylaian M, Kling MA, Reed SA, Bradham DD, Hebel JR, Barrett D, Knowles F 3rd, Hauser P. 2004. Treatment  outcomes in depression: comparison of remote treatment through telepsychiatry  to in-person treatment. *American Journal of Psychiatry.* **161:** 1471-1476.  Saeed SA, Anand V. 2015. Use of Telepsychiatry in Psychodynamic Psychiatry. *Psychodynamic Psychiatry*. **43**: 569-83.  McLaren P, Ahlbom J, Riley A, Mohammedali A, Denis M. 2002. The North Lewisham telepsychiatry project: beyond the pilot phase. *Journal of Telemedicine and Telecare.* **8:** 90-100.  Sales, C.P., McSweeney, L., Saleem, Y., Khalifa, N. 2018. The use of telepsychiatry within forensic practice: A literature review on the use of videolink-A ten-year follow-up. *Journal of Forensic Psychiatry & Psychology.* **29:** 387-402.  Setterberg, S.R., Busseri, M.A., Fleissner, R.M., Kenney, E.M., Flom, J.A., Fischer, K.J. 2003. Remote assessment of the use of seclusion and restraint with paediatric psychiatric patients. *Journal of Telemedicine and Telecare.* **9:** 176-179 |
| Patient & Clinician Satisfaction | 2011. A telepsychiatry solution for rural eastern Texas. Burke Center Mental Health Emergency Center, Lufkin, Texas. *Psychiatric Services*. **62**:1384-6.  Aadil M, Cosme RM, Forcen FE, Khan AR. 2017. A Call for Emergency Action: Telepsychiatry for Trauma Treatment Among Syrian Refugees. *Cureus*. 2017; **18**:e1578.  Abba-Aji, A. 2006. Telepsychiatry: A solution to clinical efficacy or cost effectiveness. *Irish Journal of Psychological Medicine.* **23:**84.  Abdi YA, Elmi JY. 2011. Internet based telepsychiatry: a pilot case in Somaliland. *Medicine, Conflict and Survival.* **27**:145-50.  Aboujaoude, E., Gega. 2020. From digital mental health interventions to digital "addiction": Where the two fields converge. *Frontiers in Psychiatry.* 10.  Agarwal PP, Manjunatha N, Gowda GS, Kumar MNG, Shanthaveeranna N, Kumar CN, Math SB. 2019. Collaborative Tele-Neuropsychiatry Consultation Services for Patients in Central Prisons. *Journal of Neurosciences in Rural Practice.* **10**:101-105.  Alexander J, Lattanzio A. Utility of telepsychiatry for Aboriginal Australians. *Australian & New Zealand Journal of Psychiatry. 2009;* **43:** 1185.  Amirsadri A, Burns J, Pizzuti A, Arfken CL. 2017. Home-Based Telepsychiatry in US Urban Area. *Case Reports in Psychiatry.* **2017:**6296423.  Bashshur RL, Shannon GW, Bashshur N, Yellowlees PM. 2016. The  Empirical Evidence for Telemedicine Interventions in Mental Disorders. *Telemedicine and e-Health.*  **22**:87-113  Batastini, Ashley B, McDonald, Brendan R & Morgan, Robert D. 2013. Videoteleconferencing in forensic and correctional practice. Myers, Kathleen [Ed], Turvey, Carolyn L [Ed]. Telemental health: Clinical, technical, and administrative foundations for evidence-based practice. Amsterdam, Netherlands: Elsevier, Netherlands; pp. 251-271.  Behere PB, Mansharamani HD, Kumar K. 2017. Telepsychiatry: Reaching the unreached. *Indian Journal of Medical Research.* **146:** 150-152.  Ben-Zeev D. 2020. The Digital Mental Health Genie Is Out of the Bottle. *Psychiatric Services*. **24**:appips202000306.  Bishop J.E., O’Reilly R.L., Maddox K., Hutchinson L.J. 2002. Client satisfaction in a feasibility study comparing face-to-face interviews with telepsychiatry. *Journal of telemedicine and telecare*. **8**: 217-221  Bahloul, H.J., Mani, N. 2013. International telepsychiatry: A review of what has been published. *Journal of Telemedicine and Telecare.* **19**: 293-294.  Blackmon, L.A., Kaak, H.O., Ranseen, J. 1997. Consumer satisfaction with telemedicine child psychiatry consultation in rural Kentucky. *Psychiatric Services.* **48**: 1464-1466  Bolle R.R., Trondsen M.V., Stensland G.Ø., Tjora A. 2018. Usefulness of  videoconferencing in psychiatric emergencies -- a qualitative study. *Health and Technology.*  **8:** 111-117.  Borders C.B. 2017. Realizing the Promises of Telepsychiatry in Special Populations. *Mental Illness Journal.* **9:** 7135.  Boydell KM, Hodgins M, Pignatiello A, Teshima J, Edwards H, Willis D. 2014. Using technology to deliver mental health services to children and youth: a scoping review. *Journal of the Canadian Academy of Child and Adolescent Psychiatry*. **23**:87-99.  Boydell KM, Volpe T, Pignatiello A. 2010. A qualitative study of young people's perspectives on receiving psychiatric  services via televideo. *Journal of the Canadian Academy of Child and Adolescent Psychiatry*. **19**:5-11.  Boydell, K.M., Volpe, T., Kertes, A., Greenberg, N. 2007. A review of the outcomes of the recommendations made during paediatric telepsychiatry consultations. *Journal of Telemedicine and Telecare.* **13:** 277-281.  Buist A, Coman G, Silvas A, Burrows G. 2000. An evaluation of the telepsychiatry programme in Victoria, Australia. *Journal of Telemedicine and Telecare.* **6**: 216-21.  Butterfield, A. 2018. Telepsychiatric evaluation and consultation in emergency care settings. *Child and Adolescent Psychiatric Clinics of North America.* **27:** 467-478.  Campbell R, O'Gorman J, Cernovsky ZZ. 2015. Reactions of Psychiatric Patients to Telepsychiatry. *Mental Illness Journal.* **7**: 6101.  Rachal, J., Sparks, W., Zazzaro C., Blackwell T. 2015. Highlights in Telepsychiatry and Behavioural Health Emergencies.. *Psychiatric Clinics of North America.* **40:** 585-596  Cerda, G.M., Hilty, D.M., Hales, R.E., Nesbitt, T.S. 1999. Use of telemedicine with ethnic groups. *Psychiatric Services.* **50:** 1364.  Chakrabarti S. 2015. Usefulness of telepsychiatry: A critical evaluation of videoconferencing-based  approaches. *World Journal of Psychiatry.* **22:** 286-304.  Chan S, Parish M, Yellowlees P. 2015. Telepsychiatry Today.*Current Psychiatry Reports.* **17:** 89  Cheng KM, Siu BW, Au Yeung CC, Chiang TP, So MH, Yeung MC. 2018. Telepsychiatry for stable Chinese psychiatric out-patients in custody in Hong Kong: a case-control pilot study. *Hong Kong Medical Journal.* **24:** 378-383.  Chipps J, Brysiewicz P, Mars M. 2012. Effectiveness and feasibility of telepsychiatry in resource constrained environments? A systematic review of the evidence. *African Journal of Psychiatry.* **15**: 235-43.  Caudill, R.L., Sager, Z. 2015. Institutionally based videoconferencing. *International Review of Psychiatry*. **27:** 496-503.  Chipps J, Ramlall S, Madigoe T, King H, Mars M. Developing telepsychiatry services in KwaZulu-Natal -- an action research study. *African Journal of Psychiatry*. 2012. **15:** 255-63.  Chipps J, Ramlall S, Mars M. 2012. Practice guidelines for videoconference-based telepsychiatry in South Africa. *African Journal of Psychiatry.* **15:** :271-82.  Chong, J., Moreno, F. 2012. Feasibility and acceptability of clinic-based telepsychiatry for low-income Hispanic primary care patients. *Telemedicine and e-Health.* **18**: 297-304  American Academy of Child and Adolescent Psychiatry (AACAP) Committee on Telepsychiatry and AACAP Committee on Quality Issues. 2017. Clinical Update: Telepsychiatry With Children and Adolescents. *Journal of the American Academy of Child & Adolescent Psychiatry.* **56:** 875-893  Corruble E. 2020. A Viewpoint From Paris on the COVID-19 Pandemic: A Necessary Turn to Telepsychiatry. *Journal of Clinical Psychiatry.* **31:** 81  Cowan KE, McKean AJ, Gentry MT, Hilty DM. 2019. Barriers to Use of Telepsychiatry: Clinicians as Gatekeepers. *Mayo Clinic Proceedings.* **94:** 2510-2523.  Crowe T, Jani S, Jani S, Jani N, Jani R. 2016. A pilot program in rural telepsychiatry for deaf and hard of hearing  populations. *Heliyon*. **2**: e00077  Cruz, M.K., Elizabeth A., Lopez, A.M., Weinstein, R.S. 2005. A review of the first five years of the University of Arizona telepsychiatry programme. *Journal of Telemedicine and Telecare.* **11:** 234-239  De Las Cuevas C, A.J, De La Fuente J., Serrano P. 2003. Telepsychiatry in the Canary Islands: user acceptance and satisfaction. *Journal of Telemedicine and Telecare.* **9**: 221-4.  Deslich S, Stec B, Tomblin S, Coustasse A. 2013. Telepsychiatry  in the 21(st) century: transforming healthcare with technology. *Perspectives in Health Information Management.* **10:** 1f.  Deslich SA, Thistlethwaite T, Coustasse A. 2013. Telepsychiatry  in correctional facilities: using technology to improve access and decrease  costs of mental health care in underserved populations. *The Permanente Journal* **17:** 80-86.  Detweiler MB, Arif S, Candelario J, Altman J, Murphy PF, Halling MH, Detweiler JG, Vasudeva S. 2011. A telepsychiatry transition clinic: the first 12 months  experience. *Journal of Telemedicine and Telecare.* **17:** 293-297.  Dham, P., Gupta, N., Alexander, J., Black, W., Rajji, T., Skinner, E. 2018. Community based telepsychiatry service for older adults residing in a rural and remote region- utilization pattern and satisfaction among stakeholders. *BMC Psychiatry.* 18.  Diamond, J.M., Bloch, R.M. 2010. Telepsychiatry assessments of child or adolescent behavior disorders: A review of evidence and issues. *Telemedicine and e-Health.* **16:** 712-716  Donley, E., McClaren, A., Jones, R., Katz, P., Goh, J. 2017. Evaluation and implementation of a telepsychiatry trial in the emergency department of a metropolitan public hospital. *Journal of Technology in Human Services*. **35:** 292-313.  Dossetor, D. R, Nunn, K. P, Fairley, M., Eggleton, D. 1999. A child and adolescent psychiatric outreach service for rural New South Wales: A telemedicine pilot study. *Journal of Paediatrics and Child Health*. **35:** 525-529.  Egede LE, Frueh CB, Richardson LK, Acierno R, Mauldin PD, Knapp RG, Lejuez C. 2009. Rationale and design: telepsychology service delivery for depressed elderly veterans. *Trials.* **10:** 22  Ermer D.J. 1999. Experience with a rural telepsychiatry clinic for children and adolescents. *Psychiatric Services.* **50:** 260-261.  Gammon D, Bergvik S, Bergmo T, Pedersen S.1996. Videoconferencing  in psychiatry: a survey of use in northern Norway. *Journal of Telemedicine and Telecare.* **2:** 192-198.  Gardner JS, Plaven BE, Yellowlees P, Shore JH. 2020. Remote Telepsychiatry Workforce: a Solution to Psychiatry's Workforce Issues. *Current Psychiatric Reports.* **22:** 8.  Gibson, K., O'Donnell, S., Coulson, H., Kakepetum-Schultz, T. 2011. Mental health professionals' perspectives of telemental health with remote and rural First Nations communities. *Journal of Telemedicine and Telecare.* **17:** 263-267.  Doze S, Simpson J, Hailey D, Jacobs P. 1999. Evaluation of a telepsychiatry pilot project. *Journal of Telemedicine and Telecare.* **5:** 38-46  Elford DR, White H, St John K, Maddigan B, Ghandi M, Bowering R. 2001. A prospective satisfaction study and cost analysis of a pilot child telepsychiatry service in Newfoundland. *Journal of Telemedicine and Telecare.* **7:** 73-81.  Gelber H. 2001. The experience in Victoria with telepsychiatry for the child and adolescent mental health service. *Journal of Telemedicine and Telecare.* **7:** 32-4.  Elford R., White H., Bowering R., Ghandi A., Maddiggan B., St John K., House M., Harnett J., West R., Battcock A. 2000. A randomized, controlled trial of child psychiatric assessments conducted using videoconferencing. *Journal of telemedicine and telecare.* **6:** 73-82  Gloff, N.E, LeNoue, S.R, Novins, D.K., Myers, K. 2015. Telemental health for children and adolescents. *International Review of Psychiatry*. **27:** 513-524.  Glueck DA. 2011. Telepsychiatry in private practice. *Child and Adolescent Psychiatric Clinics of North America*. **20**: 1-11.  Gowda, Guru S, Kulkarni, Karishma, Bagewadi, Virupaksha, R. P. S., Shyam, Manjunatha, B. R, Shashidhara, Harihara N, et al. 2018. A study on collaborative telepsychiatric consultations to outpatients of district hospitals of Karnataka, India. *Asian Journal of Psychiatry.* **37:** 161-166  Gratzer D, Torous J, Lam RW, Patten SB, Kutcher S, Chan S, Vigo D, Pajer K, Yatham LN. 2020. Our Digital Moment: Innovations and Opportunities in  Digital Mental Health Care. *The Canadian Journal of Psychiatry.*  Grubaugh, Anouk L, Cain, Gregory D, Elhai, Jon D, Patrick, Sarah L & Frueh, B. Christopher. 2008. Attitudes toward medical and mental health care delivered via telehealth applications among rural and urban primary care patients. *Journal of Nervous and Mental Disease.* **19:** 166-170.  Gunter, T.D, Srinivasaraghavan, J. Terry, N.P. 2003. Misinformed Regulation of Electronic Medicine Is Unfair to Responsible Telepsychiatry. *Journal of the American Academy of Psychiatry and the Law.* **31:** 10-14.  Haghnia Y., Samad-Soltani T., Yousefi M., Sadr H., Rezaei-Hachesu P. 2010. Telepsychiatry-based care for the treatment follow-up of Iranian war veterans with post-traumatic stress disorder: a randomized controlled trial. *Iranian journal of medical sciences*. **44**: 291-298.  Graham, M.A. 1996. Telepsychiatry in Appalachia. *American Behavioral Scientist*. **39:** 602-615.  Hailey D, Jacobs P, Simpson J, Doze S. 1999. An assessment framework for telemedicine applications. *Journal of telemedicine and telecare.* **5**:162-70.  Hariman K, Ventriglio A, Bhugra D. 2019. The Future of Digital Psychiatry. *Current Psychiatry Reports.* **13:** 88.  Hariman K, Ventriglio A, Bhugra D. 2019. The Future of Digital Psychiatry. *Current Psychiatry Reports.* **13:** 88.  Harley, J., McLaren, P., Blackwood, G., Tierney, K., Everett, M. 2002. The use of videoconferencing to enhance tertiary mental health service provision to the Island of Jersey. *Journal of Telemedicine and Telecare*. **8:** 36-38.  Harley, J. 2006. Economic evaluation of a tertiary telepsychiatry service to an island. *Journal of Telemedicine and Telecare*. **12:** 354-357.  Hasselberg MJ. 2020. The Digital Revolution in Behavioral Health. *Journal of the American Psychiatric Nurses Association.*  **26:** 102-111.  Hensel J, Graham R, Isaak C, Ahmed N, Sareen J, Bolton J. 2020. A Novel Emergency Telepsychiatry Program in a Canadian Urban Setting: Identifying and Addressing Perceived Barriers for Successful Implementation: Un nouveau programme de télépsychiatrie d'urgence en milieu urbain canadien: Identifier et aborder les obstacles perçus d'une mise en œuvre réussie. *Canadian Journal of Psychiatry.* **65:** 559-567.  Hilty DM, Crawford A, Teshima J, Chan S, Sunderji N, Yellowlees PM, Kramer G, O'neill P, Fore C, Luo J, Li ST. 2015. A framework  for telepsychiatric training and e-health: Competency-based education, evaluation and implications. *International Review of Psychiatry.* **27:** 569-592.  Hilty DM, Ferrer DC, Parish MB, Johnston B, Callahan EJ, Yellowlees PM. 2013. The effectiveness of telemental health. *Journal of telemedicine and telecare.* **19:** 444-454.  Hilty DM, Gentry MT, McKean AJ, Cowan KE, Lim RF, Lu FG. 2020. Telehealth for rural diverse populations: telebehavioral and cultural competencies, clinical outcomes and administrative approaches. *mHealth Journal*. **6**: 20  Hilty DM, Luo JS, Morache C, Marcelo, Divine A & Nesbitt, TS. 2002. Telepsychiatry: An overview for psychiatrists. *CNS Drugs.* **16:** 527-548.  Hilty DM, Yellowlees PM, Parrish MB, Chan S. 2015. Telepsychiatry: Effective, Evidence-Based, and at a Tipping Point in Health Care Delivery?. *Psychiatric Clinics of North America.* **38**: 559-92.  Hockey AD, Yellowlees PM, Murphy S. 2004. Evaluation  of a pilot second-opinion child telepsychiatry service. *Journal of telemedicine and telecare*. **10**: 48-50.  Hubley S, Lynch SB, Schneck C, Thomas M, Shore J. 2016. Review  of key telepsychiatry outcomes. *World Journal of Psychiatry*. **22:** 269-82  Hulsbosch, A.M., Nugter, M.A., Tamis, P., Kroon, H. 2017. Videoconferencing in a mental health service in The Netherlands: A randomized controlled trial on patient satisfaction and clinical outcomes for outpatients with severe mental illness. *Journal of Telemedicine and Telecare*. **23:** 513-520.  Hungerbuehler I, Valiengo L, Loch AA, Rössler W, Gattaz WF. 2016. Home-Based  Psychiatric Outpatient Care Through Videoconferencing for Depression: A  Randomized Controlled Follow-Up Trial. *Journal of Medical Internet Research.* **3:** e36.  Hungerbuehler I., Leite R.F.M., van de Bilt M.T., Gattaz W.F. 2015. A randomized clinical trial of home-based telepsychiatric outpatient care via videoconferencing: design, methodology, and implementation. *Revista de psiquiatria clinica.* **42**: 76-78.  Jacob MK, Larson JC, Craighead WE. 2012. Establishing a telepsychiatry consultation practice in rural Georgia for primary care  physicians: a feasibility report. *Clinical Paediatrics.* **51:** 1041-1047.  Jefee B.H., Mani N. International telepsychiatry: a review of what has been published. *Journal* Telemed Telecare. 2013; **19:** 293-4.  Jefee-Bahloul H. 2014. Telemental health in the middle East: overcoming the barriers. *Frontiers in Public Health*. **2:** 86  Jones, Roland M, Leonard, S., Birmingham, L. Setting up a telepsychiatry service. *Psychiatric Bulletin*. 2006; **30:** 464-467  Kaftarian E. Lessons Learned in Prison and Jail-Based Telepsychiatry. *Current Psychiatric Reports*. **21:** 15.  Kalin ML, Garlow SJ, Thertus K, Peterson MJ. Rapid Implementation of Telehealth in Hospital Psychiatry in Response to COVID-19. *American Journal of Psychiatry.* **177**: 636-637.  Karlinsky H. 2004. Psychiatry, Technology, and the Corn Fields of Iowa. *The Canadian Journal of Psychiatry / La Revue canadienne de psychiatri*e, **49:** 1-3.  Katz CL, Washington FB, Sacco M, Schuetz-Mueller J. A Resident-Based Telepsychiatry Supervision Pilot Program in Liberia. *Psychiatric Services.* **70:** 243-246.  Kavanagh S, Hawker F. 2001. The fall and rise of the South Australian telepsychiatry network. *Journal of Telemedicine and Telecare*. **7**: 41-3.  Keilman, P. 2005. Telepsychiatry with Child Welfare Families Referred to a Family Service Agency. *Telemedicine and e-Health.* **11:** 98-101.  Kennedy C, Yellowlees P. 2000. A community-based approach to evaluation of health outcomes and costs for telepsychiatry in a rural population: preliminary results. *Journal of Telemedicine and Telecare*. **6:** S155-7.  Khalifa, N, Saleem, Y., Stankard, P. 2008. The use of telepsychiatry within forensic practice: A literature review on the use of videolink. *Journal of Forensic Psychiatry & Psychology.* **19:** 2-13.  Kimmel, R.J., Toor, R. 2019. Telepsychiatry by a public, academic medical center for inpatient consults at an unaffiliated, community hospital. Psychosomatics: *Journal of Consultation and Liaison Psychiatry*. **60**: 468-473.  Kopel H, Nunn K, Dossetor D. 2001. Evaluating satisfaction with a child and adolescent psychological telemedicine outreach  service. *Journal of Telemedicine and Telecare*. **7**: 35-40.  Kornbluh RA. 2014. Staying true to the mission: adapting telepsychiatry to a new environment. *CNS Spectrums*. **19**: 482-3.  LaBelle B, Franklyn AM, Pkh Nguyen V, Anderson KE, Eibl JK, Marsh DC. Characterizing the Use of Telepsychiatry for Patients with Opioid Use Disorder and Cooccurring Mental Health Disorders in Ontario, Canada. *International Journal of Telemedicine and Applications.* **2018:** 7937610.  Lal, S., Abdel-Baki, A., Sujanani, S., Bourbeau, F., Sahed, I., Whitehead, J. 2020. Perspectives of young adults on receiving telepsychiatry services in an urban early intervention program for first-episode psychosis: A cross-sectional, descriptive survey study. *Frontiers in Psychiatry*. 11  Lau, M.E., Way, B.B., Fremont, W.P. 2011. Assessment of SUNY Upstate Medical University's child telepsychiatry consultation program. I*nternational Journal of Psychiatry in Medicine*. **42:** 93-104  Lin, C., Bai, Y., Chen, J. 2003. Reliability of Information Provided by Patients of a Virtual Psychiatric Clinic. *Psychiatric Services.* **54:** 1167-1168.  Lingley-Pottie, P., McGrath, P.J. 2008. Telehealth: A child and family-friendly approach to mental health-care reform. *Journal of Telemedicine and Telecare*. **14:** 225-226.  Litwack S.D, Jackson C.E, Chen M, Sloan D.M, Hatgis C, Litz, B.T. & Marx B.P.. 2014. Validation of the use of video teleconferencing technology in the assessment of PTSD*. Psychological Services.* 2014; **11:** 290-294.  Madhavan G. 2019. Telepsychiatry in intellectual disability psychiatry: literature  review. *BJPsych Bulletin.* **43**: 167-173  Malhotra S, Chakrabarti S, Shah R. 2013. Telepsychiatry:Promise, potential, and challenges. *Indian Journal of Psychiatry.* **55**: 3-11.  Malhotra, S., Shah, R. 2018. Telepsychiatry and digital mental health care in child and adolescent psychiatry: Implications for service delivery in low- and middle-income countries. Hodes, Matthew [Ed], Shur-Fen Gau, Susan [Ed], De Vries, Petrus J [Ed]. Understanding uniqueness and diversity in child and adolescent mental health. San Diego, CA, US: Elsevier Academic Press, US; pp. 263-287  Mannion, L, Fahy, T. J, Duffy, C, Broderick, M., Gethins, E. 1998. 'Telepsychiatry': Keeping a link with an island. *Psychiatric Bulletin.* **22:** 47-49.  May CR, Ellis NT, Atkinson T, Gask L, Mair F, Smith C. 1999. Psychiatry by videophone:  a trial service in north west England. *Studies in Health Technology and Informatics*. **68**: 207-10.  Mazhari S, Ghaffari Nejad A, Mofakhami O, Raaii F, Bahaadinbeigy K. 2019. Evaluating  the Diagnostic Agreement between Telepsychiatry Assessment and Face-to-Face  Visit: A Preliminary Study. *Iranian Journal of Psychiatry*. **14:** 236-241.  McCann RA, Erickson JM, Palm-Cruz KJ. 2020. The  Development, Implementation, and Evaluation of a Novel Telepsychiatry  Curriculum for Integrated Care Psychiatry Fellows. *Academic Psychiatry.* **44:** 451-454.  McLaren P. 2004. Telepsychiatry in Europe. *International Psychiatry.* **1**: 8-10.  Meltzer, B. 1997. Telemedicine in emergency psychiatry. *Psychiatric Services.* **48:** 1141-1142.  Menon, A. Srikumar, K., Prasad, K., Popuri, Chrismer, J.B., Raskin, A., Hebel, J.R. et al. 2001. Evaluation of a portable low cost videophone system in the assessment of depressive symptoms and cognitive function in elderly medically ill veterans. *Journal of Nervous and Mental Disease*. **189:** 399-401.  Mettner J. 2013. The doctor is in another town: telepsychiatry brings care to people in rural Minnesota. *Minnesota Medicine*. **96**: 22-5.  Mielonen, M., Ohinmaa, A., Moring, J., Isohanni, M. 2002. Videoconferencing in telepsychiatry. *Journal of Technology in Human Services.* **20:** 183-199.  Clarke CS. 2018. Telepsychiatry in Asperger's syndrome. *Irish Journal of Psychological Medicine.* **35:** 325-328.  Ikelheimer, D.M. 2008. Treatment of opioid dependence via home- based telepsychiatry. *Psychiatric Services.* **59:** 1218-1219.  Benyakorn S. 2016. Implementing Telepsychiatry in Thailand Benefits and Challenges. *Journal of the Medical Association of Thailand*. **99**: S260-S266.  Hilty DM., Yellowlees, P.M. 2015. Collaborative mental health services using multiple technologies: The new way to practice and a new standard of practice? *Journal of the American Academy of Child & Adolescent Psychiatry.* **54:** 245-246.  Gabel S. 2009. Telepsychiatry, public mental health, and the workforce shortage in child and adolescent psychiatry. *Journal of the American Academy of Child & Adolescent Psychiatry.* **48:** 1127-1128.  Heravian A, Chang BP. 2018. Mental health and telemedicine in the acute care setting: Applications of telepsychiatry in the ED.*The American Journal of Emergency Medicine.* **36:** 1118-1119  Greenwood, J., Chamberlain, C., Parker, G. 2004. Evaluation of a rural telepsychiatry service. *Australasian Psychiatry.* **12:** 268-272.  Whitten P, Kuwahara E. 2004. A multi-phase telepsychiatry programme in Michigan: organizational factors affecting utilization and user perceptions. *Journal of Telemedicine and Telecare.* **10:** 254-261.  Mucic D. 2008. International telepsychiatry: a study of patient acceptability. *Journal of Telemedicine and Telecare.* **14:** 241-243.  Myers KM, Valentine JM, Melzer SM. 2008. Child and adolescent telepsychiatry: utilization and satisfaction.*Telemedicine and e-Health.* **14:** 131-137.  Nelson EL, Bui TN, Velasquez SE. 2011. Telepsychology: research and practice overview. *Child and Adolescent Psychiatric Clinics of North America.* **20:** 67-79.  Ye J, Shim R, Lukaszewski T, Yun K, Kim SH, Ruth G. 2012. Telepsychiatry services for Korean immigrants. *Telemedicine and e-Health*. 2012; **18:** 797-802.  Narasimhan M, Druss BG, Hockenberry JM, Royer J, Weiss P, Glick G, Marcus SC, Magill J. 2015. Impact of a Telepsychiatry Program at Emergency Departments Statewide on the Quality, Utilization, and Costs of Mental Health Services. *Psychiatric Services.* **66:** 1167-72  May C, Gask L, Ellis N, Atkinson T, Mair F, Smith C, Pidd S, Esmail A. 2000. Telepsychiatry evaluation in the north-west of England: preliminary results of a qualitative study. *Journal of Telemedicine and Telecare.* **6:** S20-22.  Matsuura S, Hosaka T, Yukiyama T, Ogushi Y, Okada Y, Haruki Y, Nakamura M. 2000. Application of telepsychiatry: a preliminary study. *Psychiatry and Clinical Neurosciences.* **54:** 55-58.  Rohland BM, Saleh SS, Rohrer JE, Romitti PA. 2000. Acceptability of telepsychiatry to a rural population. *Psychiatric Services.* **51:** 672-674.  Brodey BB, Claypoole KH, Motto J, Arias RG, Goss R. 2000. Satisfaction of forensic psychiatric patients with remote telepsychiatric evaluation. *Psychiatric Services.* **51:** 1305-1307.  May C, Gask L, Atkinson T, Ellis N, Mair F, Esmail A. 2001. Resisting and promoting new technologies in clinical practice: the case of telepsychiatry. *Social Science & Medicine.* **52:** 1889-1901.  Johnston D, Jones BN 3rd. Telepsychiatry consultations to a rural nursing facility: a 2-year experience. *Journal of Geriatric Psychiatry and Neurology.* **14:** 72-75.  Rohland BM. 2001. Telepsychiatry in the heartland: if we build it, will they come?. *Community Mental Health Journal.* **37:** 449-459.  Pollard SE, LePage JP. 2001. Telepsychiatry in a rural inpatient setting. *Psychiatric Services.* **52:** 1659.  Krupinski EA, Barker G, Lopez AM, Weinstein RS. 2004. An analysis of unsuccessful teleconsultations. *Journal of Telemedicine Telecare.* **10:** 6-10.  Myers K, Valentine J, Morganthaler R, Melzer S. 2006. Telepsychiatry with incarcerated youth. *Journal of Adolescent Health.* **38:** 643-648.  Fox KC, Connor P, McCullers E, Waters T. 2008. Effect of a behavioural health and specialty care telemedicine programme on goal attainment for youths in juvenile detention. *Journal of Telemedicine and Telecare.* **14:** 227-230.  Saleem Y, Taylor MH, Khalifa N. 2008. Forensic telepsychiatry in the United Kingdom. *Behavioural Sciences and the Law*. **26:** 333-344.  Rabinowitz T, Murphy KM, Amour JL, Ricci MA, Caputo MP, Newhouse PA. 2010. Benefits of a telepsychiatry consultation service for rural nursing home residents. *Telemedicine and e-Health.* **16:** 34-40.  Ulzen T, Williamson L, Foster PP, Parris-Barnes K. 2013. The evolution of a community-based telepsychiatry program in rural Alabama: lessons learned-a brief report. *Community Mental Health Journal.* **49:** 101-5.  Myers K. 2013. Telepsychiatry: time to connect. *Journal of the American Academy of Child and Adolescent Psychiatry.* **52:** 217-219.  Wallace D, Rayner S. 2013. Telepsychiatry services in the Australian Defence Force. *Australasian Psychiatry.* **21:** 278-279.  Jefee-Bahloul H. 2014. Use of telepsychiatry in areas of conflict: the Syrian refugee crisis as an example. *Journal of Telemedicine and Telecare.* **20:** 167-168.  Nassan M, Frye MA, Adi A, Alarcón RD. 2015. Telepsychiatry for post-traumatic stress disorder: a call for action in the Syrian conflict. *Lancet Psychiatry*. **2:** 866.  Das S, Manjunatha N, Kumar CN, Math SB, Thirthalli J. 2020. Tele-psychiatric after care clinic for the continuity of care: A pilot study from an academic hospital. *Asian Journal of Psychiatry.* **48:** 101886.  Frueh BC, Deitsch SE, Santos AB, Gold PB, Johnson MR, Meisler N, Magruder KM, Ballenger JC. 2000. Procedural and methodological issues in telepsychiatry research and program development. *Psychiatric Services.* **51:** 1522-1527.  Monnier J, Knapp RG, Frueh BC. 2003. Recent advances in telepsychiatry: an updated review. *Psychiatric Services.* **54:** 1604-1609.  McGinty KL, Saeed SA, Simmons SC, Yildirim Y. 2006. Telepsychiatry and e-mental health services: potential for improving access to mental health care. *Psychiatric Quarterly.* **77:** 335-342.  Urness D, Wass M, Gordon A, Tian E, Bulger T. 2006. Client acceptability and quality of life--telepsychiatry compared to in-person consultation. *Journal of Telemedicine and Telecare.* **12:** 251-254.  Modai I, Jabarin M, Kurs R, Barak P, Hanan I, Kitain L. 2007. Cost effectiveness, safety, and satisfaction with video telepsychiatry versus face-to-face care in ambulatory settings. *Telemedicine and e-Health*. **12:** 515-520.  Myers KM, Valentine JM, Melzer SM. 2007. Feasibility, acceptability, and sustainability of telepsychiatry for children and adolescents. *Psychiatric Services.* **58:** 1493-1496.  Yellowlees P, Burke MM, Marks SL, Hilty DM, Shore JH. 2008. Emergency telepsychiatry. *Journal of Telemedicine and Telecare.* **14:** 277-281.  García-Lizana F, Muñoz-Mayorga I. 2010. What about telepsychiatry? A systematic review.*The Primary Care Companion to the Journal of Clinical Psychiatry.* **12.**  Myers KM, Vander Stoep A, McCarty CA, Klein JB, Palmer NB, Geyer JR, Melzer SM. 2010. Child and adolescent telepsychiatry: variations in utilization, referral patterns and practice trends. *Journal of Telemedicine and Telecare.* **16:** 128-133.  Szeftel R, Mandelbaum S, Sulman-Smith H, Naqvi S, Lawrence L, Szeftel Z, Coleman S, Gross L. 2011. Telepsychiatry for children with developmental disabilities: applications for patient care and medical education. *Child and Adolescent Psychiatric Clinics in North America.* **20:** 95-111.  Pignatiello A, Teshima J, Boydell KM, Minden D, Volpe T, Braunberger PG. 2011. Child and youth telepsychiatry in rural and remote primary care. *Child and Adolescent Psychiatric Clinics in North America.* **20:** 13-28.  Grady BJ, Lever N, Cunningham D, Stephan S. 2011. Telepsychiatry and school mental health. *Child and Adolescent Psychiatric Clinics in North America.* **20:** 81-94.  Wood J, Stathis S, Smith A, Krause J. 2012. E-CYMHS: an expansion of a child and youth telepsychiatry model in Queensland. *Australasian Psychiatry.* **20:P** 333-337.  Ramos-Ríos R, Mateos R, Lojo D, Conn DK, Patterson T. 2012. Telepsychogeriatrics: a new horizon in the care of mental health problems in the elderly. *International Psychogeriatrics.* **24:** 1708-1724.  Shore JH. 2013. Telepsychiatry: videoconferencing in the delivery of psychiatric care. *American Journal of Psychiatry.* **170:** 256-262.  Ellington E. 2013. Telepsychiatry by APRNs: an answer to the shortage of pediatric providers?. *Issues in Mental Health Nursing.* **34:** 719-721.  Salmoiraghi A, Hussain S. 2015. A Systematic Review of the Use of Telepsychiatry in Acute Settings. *Journal of Psychiatric Practice*. **21:** 389-393.  Shore J. 2015. The evolution and history of telepsychiatry and its impact on psychiatric care: Current implications for psychiatrists and psychiatric organizations. *International Review of Psychiatry.* **27:** 469-475.  Yellowlees P, Richard Chan S, Burke Parish M. 2015. The hybrid doctor-patient relationship in the age of technology - Telepsychiatry consultations and the use of virtual space. *International Review Psychiatry.* **27:** 476-489.  Fortney JC, Pyne JM, Turner EE, Farris KM, Normoyle TM, Avery MD, Hilty DM, Unützer J. 2015. Telepsychiatry integration of mental health services into rural primary care settings. *International Review Psychiatry.* **27:** 525-539.  Lauckner C, Whitten P. 2016. The State and Sustainability of Telepsychiatry Programs. *The Journal of Behavioural Health Services and Research.* **43:** 305-318.  Nelson EL, Cain S, Sharp S. 2017. Considerations for Conducting Telemental Health with Children and Adolescents. *Child and Adolescent Psychiatric Clinics in North America.* **26:** 77-91.  Roberts N, Hu T, Axas N, Repetti L. 2017. Child and Adolescent Emergency and Urgent Mental Health Delivery Through Telepsychiatry: 12-Month Prospective Study. *Telemedicine and e-Health.* **23:** 842-846.  Serhal E, Crawford A, Cheng J, Kurdyak P. 2017. Implementation and Utilisation of Telepsychiatry in Ontario: A Population-Based Study. *Canadian Journal of Psychiatry.* **62:** 716-725.  Swanson CL, Trestman RL. 2018. Rural Assertive Community Treatment and Telepsychiatry. *Journal of Psychiatric Practice.* **24:** 269-273.  Hassan A, Sharif K. 2019. Efficacy of Telepsychiatry in Refugee Populations: A Systematic Review of the Evidence. *Cureus.* **11**: e3984.  Roth DE, Ramtekkar U, Zeković-Roth S. 2019. Telepsychiatry: A New Treatment Venue for Pediatric Depression. *Child and Adolescent Psychiatric Clinics in North America.* **28:** 377-395.  Smith K, Ostinelli E, Macdonald O, Cipriani A. 2020. COVID-19 and telepsychiatry: an evidence-based guidance for clinicians. *Journal of Medical Internet Research - International Scientific Journal for Medical Research.* **10.**  Myers K., Vander Stoep A., McCarty C.A., Katon W. 2015. Effectiveness of a telehealth service model for treating attention-deficit/hyperactivity disorder: a community-based randomized controlled trial. Journal of the American Academy of Child and Adolescent Psychiatry. 54: 263-274.  Shulman M., John M., Kane J.M. 2017. Home-based outpatient telepsychiatry to improve adherence with treatment appointments: a pilot study. *Psychiatric services.* **68:** 743-746.  O’Reilly R., Bishop J., Maddox K., Hutchinson L., Fisman M., Takhar J. 2007. Is telepsychiatry equivalent to face-to-face psychiatry? Results from a randomized controlled equivalence trial. *Psychiatric Services*. **58:** 863-843  Fishkind, Avrim B & Cuyler, Robert N. 2013. The role of telepsychiatry. Zun, Leslie S [Ed], Chepenik, Lara G [Ed], Mallory, Mary Nan S [Ed]. Behavioral emergencies for the emergency physician. New York, NY, US: Cambridge University Press, US; pp. 303-307.  Simpson J, Doze S, Urness D, Hailey D, Jacobs P. 2001. Evaluation of a routine telepsychiatry service. *Journal of Telemedicine and Telecare.* **7:** 90-98.  Mahmoud H, Vogt E. 2019. Telepsychiatry: an Innovative Approach to Addressing the Opioid Crisis. *Journal of Behavioural Health Sciences and Research.* **46:** 680-685.  Jones BN 3rd. 2001. Telepsychiatry and geriatric care. *Current Psychiatry Reports.* **3:** 29-36.  Zaylor C, Whitten P, Kingsley C. 2000. Telemedicine services to a county jail. *Journal of Telemedicine and Telecare.* **6:** S93-5.  Zaylor C, Nelson EL, Cook DJ. 2001. Clinical outcomes in a prison telepsychiatry clinic. *Journal of Telemedicine and Telecare.* **1:** 47-49.  Yellowlees P, Nakagawa K, Pakyurek M, Hanson A, Elder J, Kales HC. 2020. Rapid  Conversion of an Outpatient Psychiatric Clinic to a 100% Virtual Telepsychiatry  Clinic in Response to COVID-19. *Psychiatric Services.* **71:** 749-752.  Work Group on Quality Issues (WGQI) & American Academy of Child and Adolescent Psychiatry (AACAP), US. 2008. Practice parameter for telepsychiatry with children and adolescents. *Journal of the American Academy of Child & Adolescent Psychiatry.* **47:** 1468-1483.  Wilshire, Thea W. 2012. Telepsychiatry services at a tribally run behavioral health clinic. *Psychological Services*. **9:** 318-319.  Whaibeh, Emile, Mahmoud, Hossam & Vogt, Emily L. 2019. Reducing the treatment gap for lgbt mental health needs: The potential of telepsychiatry. *The Journal of Behavioral Health Services & Research*. No Pagination Specified  Wallace, Duncan & Hodges, Samantha. 2018. Telepsychiatry in the Australian Defence Force: A success story. *Australasian Psychiatry*. **26:** 105-106.  Vought, Rhonda G, Grigsby, R. Kevin, Adams, Laura N & Shevitz, Stewart A. 2000. Telepsychiatry: Addressing mental health needs in Georgia. *Community Mental Health Journal.* **36:** 525-536.  Volpe T, Boydell KM, Pignatiello A. 2013. Attracting Child Psychiatrists to a Televideo Consultation Service: The TeleLink  Experience.*International Journal of Telemedicine and Applications.* **2013:** 146858.  Volicer L. 2015. Nursing home telepsychiatry. *Journal of the American Medical Directors Association*. **16**: 7-8.  Vernig, P.M. 2016. Telemental health: Digital disruption and the opportunity to expand care. *Journal of the American Psychiatric Nurses Association*. **22:** 73-75.  van Wynsberghe A, Gastmans C. 2009. Telepsychiatry and the meaning of in-person contact: a preliminary ethical appraisal. *Medicine, Health Care and Philosophy.* **12:** 469-476.  Valdagno M, Goracci A, di Volo S, Fagiolini A. 2014. Telepsychiatry: new perspectives and open issues. *CNS Spectrums.* **19:** 479-481.  Turner, J.W. 2001. Telepsychiatry as a case study of presence: Do you know what you are missing? *Journal of Computer-Mediated Communication.* **6.**  Trondsen MV, Bolle SR, Stensland GØ, Tjora A. 2014. Video-confidence:  a qualitative exploration of videoconferencing for psychiatric  emergencies. *BMC Health Service Research*. **14:** 544.  Toperczer T. 2011. Telepsychiatry in the cloud: reaching rural communities  in underserved markets. *Health Management Technology.* **32**: 28-9.  Toombs E, Kowatch KR, Dalicandro L, McConkey S, Hopkins C, Mushquash CJ. 2020. A systematic  review of electronic mental health interventions for Indigenous youth: Results  and recommendations. *Journal of Telemedicine and Telecare.* **14:** 1357633X19899231.  Thompson D.A., Leimig R., Gower G., Winsett R.P. 2009. Assessment of depressive symptoms during post-transplant follow-up care performed via telehealth. *Telemedicine journal and e-health*. **15:** 700-706.  Thomas JF, Novins DK, Hosokawa PW, Olson CA, Hunter D, Brent AS, Frunzi G, Libby AM. 2018. The Use of  Telepsychiatry to Provide Cost-Efficient Care During Pediatric Mental Health Emergencies. *Psychiatric Services.* **1:** 161-168.  Taylor M, Kikkawa N, Hoehn E, Haydon H, Neuhaus M, Smith AC, Caffery LJ. 2019. The importance of external clinical facilitation for a perinatal and infant telemental health service. *Journal of Telemedicine and Telecare.* **25:** 566-571.  Tang S, Helmeste D. 2000. Digital psychiatry. *Psychiatry and Clinical Neurosciences.* **54:** 1-10.  Swanson B. 1999. Information technology and under-served  communities. *Journal of Telemedicine and Telecare.* **5:** S3-10.  Sulzbacher S, Vallin, T, Waetzig E.Z. 2006. Telepsychiatry improves paediatric behavioural health care in rural communities. *Journal of Telemedicine and Telecare.* **12:** 285-288  Stevens, A., Doidge, N., Goldbloom, D., Voore, P., Farewell, J. 1999. Pilot study of televideo psychiatric assessments in an underserviced community. *The American Journal of Psychiatry.* **156:** 783-785  Starling, J., Foley, S. 2006. From pilot to permanent service: Ten years of paediatric telepsychiatry. *Journal of Telemedicine and Telecare.* **12:** 80-82.  Starling, J., Dossetor, D. 2005. Child and Adolescent Telepsychiatry. Wootton, Richard [Ed], Batch, Jennifer [Ed]. Telepediatrics: Telemedicine and child health. Lo: Royal Society of Medicine Press; pp. 77-87.  Spaulding, R., Cain, S., Sonnenschein, K. 2011. Urban telepsychiatry: Uncommon service for a common need. *Child and Adolescent Psychiatric Clinics of North America.* **20:** 29-39  Sousa A, Karia S. 2020. Telepsychiatry  during COVID-19: Some clinical, public health, and ethical dilemmas. *Indian Journal of Public Health.* **64:** S245-S246.  Mucic, D. 200). Telepsychiatry within European e-health. Lazakidou, Athina A [Ed], Siassiakos, Konstantinos M [Ed]. Handbook of research on distributed medical informatics and e-health. Hershey, PA, US: Medical Information Science Reference/IGI Global, US; pp. 129-136.  Pakyurek, M, Yellowlees, P., Hilty, D. 2010. The child and adolescent telepsychiatry consultation: Can it be a more effective clinical process for certain patients than conventional practice? *Telemedicine and e-Health.* **16:** 289-292.  Pesämaa L, Ebeling H, Kuusimäki ML, Winblad I, Isohanni M, Moilanen I. 2004. Videoconferencing in child and adolescent telepsychiatry: a systematic review of the literature. *Journal of Telemedicine and Telecare.* **10:** 187-192.  Reinhardt I, Gouzoulis-Mayfrank E, Zielasek J. 2019. Use of Telepsychiatry in Emergency and Crisis Intervention: Current Evidence. *Current Psychiatric Reports.* **1:** 63.  Reliford A, Adebanjo B. 2019. Use of Telepsychiatry in Pediatric Emergency Room to Decrease Length of Stay for Psychiatric Patients, Improve Resident On-Call Burden, and Reduce Factors Related to Physician Burnout. *Telemedicine and e-Health.* **25**: 828-832.  Report from the Alberta Heritage Foundation for Medical Research. 1998. Evaluation of a telepsychiatry pilot project. *International Journal of Technology Assessment in Health Care. 1998;* **14:** 583-4.  Rowe N, Gibson S, Morley S, Krupinski EA. 2008. Ten-year experience of a private nonprofit telepsychiatry service. *Telemedicine and e-Health.* **14:** 1078-1086.  McLaren P, Ahlbom J, Riley A, Mohammedali A, Denis M. 2002. The North Lewisham telepsychiatry project: beyond the pilot phase. *Journal of Telemedicine and Telecare.* **8:** 90-100.  Sales, C.P., McSweeney, L., Saleem, Y., Khalifa, N. 2018. The use of telepsychiatry within forensic practice: A literature review on the use of videolink-A ten-year follow-up. *Journal of Forensic Psychiatry & Psychology.* **29:** 387-402.  Samuels, A. 1999. International telepsychiatry: A link between New Zealand and Australia. *Australian and New Zealand Journal of Psychiatry*. **33:** 284-286.  Savin, D., Garry, M.T., Zuccaro, P., Novins, D. 2006. Telepsychiatry for treating rural American Indian Youth. *Journal of the American Academy of Child & Adolescent Psychiatry*. **45:** 484-488.  Schubert NJ, Backman PJ, Bhatla R, Corace KM. 2019. Telepsychiatry and patient-provider concordance. *Canadian Journal of Rural Medicine.* **24:** 75-82.  Seritan AL, Heiry M, Iosif AM, Dodge M, Ostrem JL. 2019. Telepsychiatry  for patients with movement disorders: a feasibility and patient satisfaction  study. *Journal of Clinical Movement Disorders.* **6:** 1.  Shores, M.M., Ryan-Dykes, P., Williams, R.M., Mamerto, B., Sadak, T., Pascualy, M. et al. 2004. Identifying undiagnosed dementia in residential care veterans: comparing telemedicine to in-person clinical examination. *International Journal of Geriatric Psychiatry.* **19:** 101-108  Simpson J, Doze S, Urness D, Hailey D, Jacobs P. 2001. Telepsychiatry  as a routine service--the perspective of the patient. *Journal of Telemedicine and Telecare.* **7:** 155-160. |
| Technology | Aboujaoude, E. 2018. Telemental health: Why the revolution has not arrived. *World Psychiatry.* **17:** 277-278  Abrams J, Sossong S, Schwamm LH, Barsanti L, Carter M, Kling N, Kotarski M, Leddy J, Meller B, Simoni M, Sullivan M, Wozniak J. Practical Issues in Delivery of Clinician-to-Patient Telemental Health in an Academic Medical Center. *Harvard Review of Psychiatry.* 2017; **25**:135-145.  Agarwal PP, Manjunatha N, Gowda GS, Kumar MNG, Shanthaveeranna N, Kumar CN, Math SB. 2019. Collaborative Tele-Neuropsychiatry Consultation Services for Patients in Central Prisons. *Journal of Neurosciences in Rural Practice.* **10**:101-105.  American Academy of Child and Adolescent Psychiatry (AACAP) Committee on Telepsychiatry and AACAP Committee on Quality Issues. 2017. Clinical update: Telepsychiatry with children and adolescents. *Journal of the American Academy of Child & Adolescent Psychiatry.* **56**: 875-893  Augusterfer EF, Mollica RF, Lavelle J. 2018. Leveraging - Technology in Post-Disaster Settings: the Role of Digital Health/Telemental Health. *Current Psychiatry Reports*. **20:** 88.  Barnett, Michael L & Huskamp, Haiden A. 2020. Telemedicine for mental health in the United States: Making progress, still a long way to go. *Psychiatric Services.* **71:** 197-198.  Batastini, Ashley B, McDonald, Brendan R & Morgan, Robert D. 2013. Videoteleconferencing in forensic and correctional practice. Myers, Kathleen [Ed], Turvey, Carolyn L [Ed]. Telemental health: Clinical, technical, and administrative foundations for evidence-based practice. Amsterdam, Netherlands: Elsevier, Netherlands; pp. 251-271.  Boydell KM, Hodgins M, Pignatiello A, Teshima J, Edwards H, Willis D. 2014. Using technology to deliver mental health services to children and youth: a scoping review. *Journal of the Canadian Academy of Child and Adolescent Psychiatry*. **23**:87-99.  Boydell, K.M., Volpe, T., Kertes, A., Greenberg, N. 2007. A review of the outcomes of the recommendations made during paediatric telepsychiatry consultations. *Journal of Telemedicine and Telecare.* **13:** 277-281.  Brown FW. 1998. Rural telepsychiatry. *Psychiatric Services.* **49**:963-4.  Buist A, Coman G, Silvas A, Burrows G. 2000. An evaluation of the telepsychiatry programme in Victoria, Australia. *Journal of Telemedicine and Telecare.* **6**: 216-21.  Campbell R, O'Gorman J, Cernovsky ZZ. 2015. Reactions of Psychiatric Patients to Telepsychiatry. *Mental Illness Journal.* **7**: 6101.  Chakrabarti S. 2015. Usefulness of telepsychiatry: A critical evaluation of videoconferencing-based  approaches. *World Journal of Psychiatry.* **22:** 286-304.  Chan S, Parish M, Yellowlees P. 2015. Telepsychiatry Today.*Current Psychiatry Reports.* **17:** :89  Caudill, R.L., Sager, Z. 2015. Institutionally based videoconferencing. *International Review of Psychiatry*. **27:** 496-503.  Chipps J, Ramlall S, Madigoe T, King H, Mars M. 2012. Developing telepsychiatry services in KwaZulu-Natal -- an action research study. *African Journal of Psychiatry*. **15:** 255-63.  Chipps J, Ramlall S, Mars M. 2012. A telepsychiatry model to support psychiatric outreach in the public sector in South Africa. *African Journal of Psychiatry.* **15**:264-70.  Chipps J, Ramlall S, Mars M. 2012. Practice guidelines for videoconference-based telepsychiatry in South Africa. *African Journal of Psychiatry.* **15:** :271-82.  Chong, J., Moreno, F. 2012. Feasibility and acceptability of clinic-based telepsychiatry for low-income Hispanic primary care patients. *Telemedicine and e-Health.* **18**: 297-304  American Academy of Child and Adolescent Psychiatry (AACAP) Committee on Telepsychiatry and AACAP Committee on Quality Issues. 2017. Clinical Update: Telepsychiatry With Children and Adolescents. *Journal of the American Academy of Child & Adolescent Psychiatry.* **56:** 875-893  Cowan KE, McKean AJ, Gentry MT, Hilty DM. 2019. Barriers to Use of Telepsychiatry: Clinicians as Gatekeepers. *Mayo Clinic Proceedings.* **94:** 2510-2523.  Deslich S, Stec B, Tomblin S, Coustasse A. 2013. Telepsychiatry  in the 21(st) century: transforming healthcare with technology. *Perspectives in Health Information Management.* **10:** 1f.  Deslich SA, Thistlethwaite T, Coustasse A. 2013. Telepsychiatry  in correctional facilities: using technology to improve access and decrease  costs of mental health care in underserved populations. *The Permanente Journal* **17:** 80-86.  Detweiler MB, Arif S, Candelario J, Altman J, Murphy PF, Halling MH, Detweiler JG, Vasudeva S. 2011. A telepsychiatry transition clinic: the first 12 months  experience. *Journal of Telemedicine and Telecare.* **17:** 293-297.  Dham, P., Gupta, N., Alexander, J., Black, W., Rajji, T., Skinner, E. 2018. Community based telepsychiatry service for older adults residing in a rural and remote region- utilization pattern and satisfaction among stakeholders. *BMC Psychiatry.* 18.  Donley, E., McClaren, A., Jones, R., Katz, P., Goh, J. 2017. Evaluation and implementation of a telepsychiatry trial in the emergency department of a metropolitan public hospital. *Journal of Technology in Human Services*. **35:** 292-313.  Fatehi, F., Armfield, N.R., Dimitrijevic, M., Gray, L.C. 2014. Clinical applications of videoconferencing: A scoping review of the literature for the period 2002-2012. J*ournal of Telemedicine and Telecare*. **20:** 377-383.  Fegert JM, Vitiello B, Plener PL, Clemens V. 2020. Challenges and  burden of the Coronavirus 2019 (COVID-19) pandemic for child and adolescent  mental health: a narrative review to highlight clinical and research needs in  the acute phase and the long return to normality. *Child and Adolescent Psychiatry and Mental Health.* **14:** 20.  Fortney JC, Heagerty PJ, Bauer AM, Cerimele JM, Kaysen D, Pfeiffer PN, Zielinski MJ, Pyne JM, Bowen D, Russo J, Ferro L, Moore D, Nolan JP, Fee FC, Heral T, Freyholtz-London J, McDonald B, Mullins J, Hafer E, Solberg L, Unützer J. 2020. Study to promote innovation in rural integrated telepsychiatry (SPIRIT): Rationale and design of a randomized comparative effectiveness trial of managing complex psychiatric disorders in rural primary care clinics. *Contemporary Clinical Trials.* **90:**  105873.  Gammon D, Bergvik S, Bergmo T, Pedersen S.1996. Videoconferencing  in psychiatry: a survey of use in northern Norway. *Journal of Telemedicine and Telecare.* **2:** 192-198.  Gardner JS, Plaven BE, Yellowlees P, Shore JH. 2020. Remote Telepsychiatry Workforce: a Solution to Psychiatry's Workforce Issues. *Current Psychiatric Reports.* **22:** 8.  Elford DR, White H, St John K, Maddigan B, Ghandi M, Bowering R. 2001. A prospective satisfaction study and cost analysis of a pilot child telepsychiatry service in Newfoundland. *Journal of Telemedicine and Telecare.* **7:** 73-81.  Gabel S. 2009. Telepsychiatry, public mental health, and the workforce shortage in child and adolescent psychiatry. *Journal of the American Academy of Child & Adolescent Psychiatry.* **48:** 1127-1128.  Gelber H, Alexander M. 1999. An evaluation of an Australian videoconferencing project for child and adolescent telepsychiatry. *Journal of Telemedicine and Telecare.* **5**: S21-3.  Gelber H. 2001. The experience in Victoria with telepsychiatry for the child and adolescent mental health service. *Journal of Telemedicine and Telecare.* **7:** 32-4.  Elford R., White H., Bowering R., Ghandi A., Maddiggan B., St John K., House M., Harnett J., West R., Battcock A. 2000. A randomized, controlled trial of child psychiatric assessments conducted using videoconferencing. *Journal of telemedicine and telecare.* **6:** 73-82  Gloff, N.E, LeNoue, S.R, Novins, D.K., Myers, K. 2015. Telemental health for children and adolescents. *International Review of Psychiatry*. **27:** 513-524.  Glueck DA. 2011. Telepsychiatry in private practice. *Child and Adolescent Psychiatric Clinics of North America*. **20**: 1-11.  Gowda, Guru S, Kulkarni, Karishma, Bagewadi, Virupaksha, R. P. S., Shyam, Manjunatha, B. R, Shashidhara, Harihara N, et al. 2018. A study on collaborative telepsychiatric consultations to outpatients of district hospitals of Karnataka, India. *Asian Journal of Psychiatry.* **37:** 161-166  Grady B. 2012. Promises and limitations of telepsychiatry in rural adult mental health care. *World Psychiatry.* **11**: 199-201.  Grubaugh, Anouk L, Cain, Gregory D, Elhai, Jon D, Patrick, Sarah L & Frueh, B. Christopher. 2008. Attitudes toward medical and mental health care delivered via telehealth applications among rural and urban primary care patients. *Journal of Nervous and Mental Disease.* **19:** 166-170.  Haghnia Y., Samad-Soltani T., Yousefi M., Sadr H., Rezaei-Hachesu P. 2010. Telepsychiatry-based care for the treatment follow-up of Iranian war veterans with post-traumatic stress disorder: a randomized controlled trial. *Iranian journal of medical sciences*. **44**: 291-298.  Graham, M.A. 1996. Telepsychiatry in Appalachia. *American Behavioral Scientist*. **39:** 602-615.  Hailey D, Jacobs P, Simpson J, Doze S. 1999. An assessment framework for telemedicine applications. *Journal of telemedicine and telecare.* **5**:162-70.  Hailey, David, Ohinmaa, Arto & Roine, Risto. 2009. Limitations in the routine use of telepsychiatry. *Journal of Telemedicine and Telecare.* **15:** 28-31.  Hampton, T. 2006. Researchers provide psychiatric care from afar. *The Journal of the American Medical Association*: **295:** 21-22.  Haslam R, McLaren P. Interactive  television for an urban adult mental health service: the Guy's Psychiatric  Intensive Care Unit Telepsychiatry Project. *Journal of Telemedicine and Telecare. 2000.* **6:** S50-2  Hilt, R.J. Telemedicine for child collaborative or integrated care. *Child and Adolescent Psychiatric Clinics of North America. 2017.* **26:** 637-645.  Hilty DM, Ferrer DC, Parish MB, Johnston B, Callahan EJ, Yellowlees PM. 2013. The effectiveness of telemental health. *Journal of telemedicine and telecare.* **19:** 444-454.  Hilty DM, Luo, John S, Morache, Chris, Marcelo, Divine A & Nesbitt, Thomas S. 2002. Telepsychiatry: An overview for psychiatrists. *CNS Drugs.* **16:** 527-548.  Hilty DM, Marks, S.L., Urness, D., Yellowlees, P.M., Nesbitt, T.S.2004. Clinical and Educational Telepsychiatry Applications: A Review. *The Canadian Journal of Psychiatry / La Revue canadienne de psychiatrie*. **49:** 12-23.  Hungerbuehler I, Valiengo L, Loch AA, Rössler W, Gattaz WF. 2016. Home-Based  Psychiatric Outpatient Care Through Videoconferencing for Depression: A  Randomized Controlled Follow-Up Trial. *Journal of Medical Internet Research.* **3:** e36.  Jacob MK, Larson JC, Craighead WE. 2012. Establishing a telepsychiatry consultation practice in rural Georgia for primary care  physicians: a feasibility report. *Clinical Paediatrics.* **51:** 1041-1047.  Jefee-Bahloul H. 2014. Telemental health in the middle East: overcoming the barriers. *Frontiers in Public Health*. **2:** 86  Jones, Roland M, Leonard, S., Birmingham, L. 2006. Setting up a telepsychiatry service. *Psychiatric Bulletin*. **30:** 464-467  Kaftarian E. Lessons Learned in Prison and Jail-Based Telepsychiatry. *Current Psychiatric Reports*. **21:** 15.  Kannarkat JT, Smith NN, McLeod-Bryant SA. Mobilization of Telepsychiatry in Response to COVID-19-Moving Toward 21st Century  Access to Care. *Administration and Policy in Mental Health.* **47**:489-491.  Katz CL, Washington FB, Sacco M, Schuetz-Mueller J. A Resident-Based Telepsychiatry Supervision Pilot Program in Liberia. *Psychiatric Services.* **70:** 243-246.  Kennedy C, Yellowlees P. 2000. A community-based approach to evaluation of health outcomes and costs for telepsychiatry in a rural population: preliminary results. *Journal of Telemedicine and Telecare*. **6:** S155-7.  Khalifa, N, Saleem, Y., Stankard, P. 2008. The use of telepsychiatry within forensic practice: A literature review on the use of videolink. *Journal of Forensic Psychiatry & Psychology.* **19:** 2-13.  Kopel H, Nunn K, Dossetor D. 2001. Evaluating satisfaction with a child and adolescent psychological telemedicine outreach  service. *Journal of Telemedicine and Telecare*. **7**: 35-40.  LaBelle B, Franklyn AM, Pkh Nguyen V, Anderson KE, Eibl JK, Marsh DC. 2018. Characterizing the Use of Telepsychiatry for Patients with Opioid Use Disorder and Cooccurring Mental Health Disorders in Ontario, Canada. *International Journal of Telemedicine and Applications.* **2018:** 7937610.  Lal, S., Abdel-Baki, A., Sujanani, S., Bourbeau, F., Sahed, I., Whitehead, J. 2020. Perspectives of young adults on receiving telepsychiatry services in an urban early intervention program for first-episode psychosis: A cross-sectional, descriptive survey study. *Frontiers in Psychiatry*. 11  Langarizadeh M, Tabatabaei MS, Tavakol K, Naghipour M, Rostami A, Moghbeli F. 2017. Telemental Health Care, an Effective Alternative to Conventional Mental Care: a Systematic  Review. *Acta Informatica Medica*. **25:** 240-246.  Lee A, S.N., O'Connell F, Dyer A, Boniface K, Betz J. Telepsychiatric assessment of a mariner expressing suicidal ideation. *International Maritime Health.2 015;* **66**: 49-51.  Leonard S. 2004. The successes and challenges of developing a prison telepsychiatry service. *Journal of Telemedicine and Telecare*. **10**: 69-71.  Lexcen, F.J., Hawk, G.L., Herrick, S., Blank, M.B. 2006. Use of Video Conferencing for Psychiatric and Forensic Evaluations. *Psychiatric Services.* **57:** 713-715.  Looi JC, Pring W. 2020. Private metropolitan telepsychiatry in Australia during  Covid-19: current practice and future developments. *Australas Psychiatry*. **2**: 1039856220930675.  Madhavan G. 2019. Telepsychiatry in intellectual disability psychiatry: literature  review. *BJPsych Bulletin.* **43**: 167-173  Malhotra S, Chakrabarti S, Shah R. 2013. Telepsychiatry:Promise, potential, and challenges. *Indian Journal of Psychiatry.* **55**: 3-11.  Malhotra, S., Shah, R. 2018. Telepsychiatry and digital mental health care in child and adolescent psychiatry: Implications for service delivery in low- and middle-income countries. Hodes, Matthew [Ed], Shur-Fen Gau, Susan [Ed], De Vries, Petrus J [Ed]. Understanding uniqueness and diversity in child and adolescent mental health. San Diego, CA, US: Elsevier Academic Press, US; pp. 263-287  Mannion, L, Fahy, T. J, Duffy, C, Broderick, M., Gethins, E. 1998. 'Telepsychiatry': Keeping a link with an island. *Psychiatric Bulletin.* **22:** 47-49.  Math SB, Moirangthem S, Kumar NC. 2015. Tele-Psychiatry: After Mars, Can we Reach the Unreached?. *Indian Journal of Psychological Medicine.* **37:** 120-1.  May CR, Ellis NT, Atkinson T, Gask L, Mair F, Smith C. 1999. Psychiatry by videophone:  a trial service in north west England. *Studies in Health Technology and Informatics*. **68**: 207-10.  McGrath J. 2020. ADHD and  Covid-19: Current roadblocks and future opportunities. *The Irish Journal of Psychological Medicine.* **21:** 1-22.  McLaren P. 2004. Telepsychiatry in Europe. *International Psychiatry.* **1**: 8-10.  Meltzer, B. 1997. Telemedicine in emergency psychiatry. *Psychiatric Services.* **48:** 1141-1142.  Menon, A. Srikumar, K., Prasad, K., Popuri, Chrismer, J.B., Raskin, A., Hebel, J.R. et al. 2001. Evaluation of a portable low cost videophone system in the assessment of depressive symptoms and cognitive function in elderly medically ill veterans. *Journal of Nervous and Mental Disease*. **189:** 399-401.  Mettner J. 2013. The doctor is in another town: telepsychiatry brings care to people in rural Minnesota. *Minnesota Medicine*. **96**: 22-5.  Mielonen, M., Ohinmaa, A., Moring, J., Isohanni, M. 2002. Videoconferencing in telepsychiatry. *Journal of Technology in Human Services.* **20:** 183-199.  Clarke CS. 2018. Telepsychiatry in Asperger's syndrome. *Irish Journal of Psychological Medicine.* **35:** 325-328.  Ikelheimer, D.M. 2008. Treatment of opioid dependence via home- based telepsychiatry. *Psychiatric Services.* **59:** 1218-1219.  Benyakorn S. 2016. Implementing Telepsychiatry in Thailand Benefits and Challenges. *Journal of the Medical Association of Thailand*. **99**: S260-S266.  Mucic D. 2008. International telepsychiatry: a study of patient acceptability. *Journal of Telemedicine and Telecare.* **14:** 241-243.  Ye J, Shim R, Lukaszewski T, Yun K, Kim SH, Ruth G. 2012. Telepsychiatry services for Korean immigrants. *Telemedicine and e-Health*. **18:** 797-802.  Werner A, Anderson LE. 1998. Rural telepsychiatry is economically unsupportable: the Concorde crashes in a cornfield. *Psychiatric Services.* **49:** 1287-1290.  Matsuura S, Hosaka T, Yukiyama T, Ogushi Y, Okada Y, Haruki Y, Nakamura M. 2000. Application of telepsychiatry: a preliminary study. *Psychiatry and Clinical Neurosciences.* **54:** 55-58.  May C, Gask L, Atkinson T, Ellis N, Mair F, Esmail A. 2001. Resisting and promoting new technologies in clinical practice: the case of telepsychiatry. *Social Science & Medicine.* **52:** 1889-1901.  Johnston D, Jones BN 3rd. Telepsychiatry consultations to a rural nursing facility: a 2-year experience. *Journal of Geriatric Psychiatry and Neurology.* **14:** 72-75.  Jones BN 3rd, Johnston D, Reboussin B, McCall WV. 2001. Reliability of telepsychiatry assessments: subjective versus observational ratings. *Journal of Geriatric Neurology and Psychiatry.* **14:** 66-71.  Miller TW, Clark J, Veltkamp LJ, Burton DC, Swope M. 2008. Teleconferencing model for forensic consultation, court testimony, and continuing education. *Behavioural Sciences and the Law.* **26:** 301-313.  Westphal A, Dingjan P, Attoe R. 2010. What can low and high technologies do for late-life mental disorders?. *Current Opinion in Psychiatry.* **23:** 510-515.  Myers KM, Palmer NB, Geyer JR. Research in child and adolescent telemental health. *Child and Adolescent Psychiatric Clinics in North America. 2011;* **20:** 155-171.  Savin D, Glueck DA, Chardavoyne J, Yager J, Novins DK. 2011. Bridging cultures: child psychiatry via videoconferencing. *Child and Adolescent Psychiatric Clinics in North America.* **20:** 125-134.  Mars M. 2012. Telepsychiatry in Africa -- a way forward?. *African Journal of Psychiatry.* **15:** 215, 217.  Myers K. 2013. Telepsychiatry: time to connect. *Journal of the American Academy of Child and Adolescent Psychiatry.* **52:** 217-219.  Jefee-Bahloul H. 2014. Use of telepsychiatry in areas of conflict: the Syrian refugee crisis as an example. *Journal of Telemedicine and Telecare.* **20:** 167-168.  Sharma A, Sasser T, Schoenfelder Gonzalez E, Vander Stoep A, Myers K. Implementation of Home-Based Telemental Health in a Large Child Psychiatry Department During the COVID-19 Crisis. *Journal of Child and Adolescent Psychopharmacology. 2020; 8.*  Frueh BC, Deitsch SE, Santos AB, Gold PB, Johnson MR, Meisler N, Magruder KM, Ballenger JC. 2000. Procedural and methodological issues in telepsychiatry research and program development. *Psychiatric Services.* **51:** 1522-1527.  Jones BN 3rd, Ruskin PE. 2001. Telemedicine and geriatric psychiatry: directions for future research and policy. *Journal of Geriatric Psychiatry and Neurology.* **14:** 59-62.  Monnier J, Knapp RG, Frueh BC. 2003. Recent advances in telepsychiatry: an updated review. *Psychiatric Services.* **54:** 1604-1609.  Miller TW, Burton DC, Hill K, Luftman G, Veltkemp LJ, Swope M. 2005. Telepsychiatry: critical dimensions for forensic services. *Journal of Academic Psychiatry and Law.* **33:** 539-546.  McGinty KL, Saeed SA, Simmons SC, Yildirim Y. 2006. Telepsychiatry and e-mental health services: potential for improving access to mental health care. *Psychiatric Quarterly.* **77:** 335-342.  Nieves JE, Stack KM. 2007. Hispanics and telepsychiatry. *Psychiatric Services.* **58**: 877-8.  García-Lizana F, Muñoz-Mayorga I. 2010. What about telepsychiatry? A systematic review.*The Primary Care Companion to the Journal of Clinical Psychiatry.* **12.**  Grady BJ, Lever N, Cunningham D, Stephan S. 2011. Telepsychiatry and school mental health. *Child and Adolescent Psychiatric Clinics in North America.* **20:** 81-94.  Ramos-Ríos R, Mateos R, Lojo D, Conn DK, Patterson T. 2012. Telepsychogeriatrics: a new horizon in the care of mental health problems in the elderly. *International Psychogeriatrics.* **24:** 1708-1724.  Shore JH. 2013. Telepsychiatry: videoconferencing in the delivery of psychiatric care. *American Journal of Psychiatry.* **170:** 256-262.  Salmoiraghi A, Hussain S. 2015. A Systematic Review of the Use of Telepsychiatry in Acute Settings. *Journal of Psychiatric Practice*. **21:** 389-393.  Nelson EL, Cain S, Sharp S. 2017. Considerations for Conducting Telemental Health with Children and Adolescents. *Child and Adolescent Psychiatric Clinics in North America.* **26:** 77-91.  Roberts N, Hu T, Axas N, Repetti L. 2017. Child and Adolescent Emergency and Urgent Mental Health Delivery Through Telepsychiatry: 12-Month Prospective Study. *Telemedicine and e-Health.* **23:** 842-846.  Saeed SA. 2018. Tower of Babel Problem in Telehealth: Addressing the Health Information Exchange Needs of the North Carolina Statewide Telepsychiatry Program (NC-STeP). *Psychiatry Quarterly.* **89:** 489-495.  Shulman M., John M., Kane J.M. 2017. Home-based outpatient telepsychiatry to improve adherence with treatment appointments: a pilot study. *Psychiatric services.* **68:** 743-746.  Simpson J, Doze S, Urness D, Hailey D, Jacobs P. 2001. Evaluation of a routine telepsychiatry service. *Journal of Telemedicine and Telecare.* **7:** 90-98.  Mahmoud H, Vogt E. 2019. Telepsychiatry: an Innovative Approach to Addressing the Opioid Crisis. *Journal of Behavioural Health Sciences and Research.* **46:** 680-685.  Zulfic Z, Liu D, Lloyd C, Rowan J, Schubert KO. 2020. Is telepsychiatry care a realistic option for community mental health services during the COVID-19 pandemic?. *Australian and New Zealand Journal of Psychiatry.* **22**;:4867420937788.  Yellowlees, Peter M, Hilty, Donald M & Mucic, Davor. 2016. Global/worldwide e-mental health: International and futuristic perspectives of telepsychiatry and the future. Mucic, Davor [Ed], Hilty, Donald M [Ed]. e-Mental health. Cham, Switzerland: Springer International Publishing, Switzerland; pp. 233-249  Yellowlees P, Nakagawa K, Pakyurek M, Hanson A, Elder J, Kales HC. 2020. Rapid  Conversion of an Outpatient Psychiatric Clinic to a 100% Virtual Telepsychiatry  Clinic in Response to COVID-19. *Psychiatric Services.* **71:** 749-752.  Work Group on Quality Issues (WGQI) & American Academy of Child and Adolescent Psychiatry (AACAP), US. 2008. Practice parameter for telepsychiatry with children and adolescents. *Journal of the American Academy of Child & Adolescent Psychiatry.* **47:** 1468-1483.  Wojtuszek, Magdalena, Kachnic, Justyna, Krysta, Krzysztof & Wutke, Joanna. 2015. Telepsychiatry in Polish patients' and doctors' opinion. *Psychiatria Danubina.* **27:** S379-S382.  Volpe T, Boydell KM, Pignatiello A. 2013. Attracting Child Psychiatrists to a Televideo Consultation Service: The TeleLink  Experience.*International Journal of Telemedicine and Applications.* **2013:** 146858.  Vernig, P.M. 2016. Telemental health: Digital disruption and the opportunity to expand care. *Journal of the American Psychiatric Nurses Association*. **22:** 73-75.  Vega, W.A., Pollitt, A., Mays, R.A. 2007. Reply to Hispanics and Telepsychiatry. *Psychiatric Services*. **58:** 878.  Vanderpool, D. 2015. An overview of practicing high quality telepsychiatry. Dewan, Naakesh A [Ed], Luo, John S [Ed], Lorenzi, Nancy M [Ed]. Mental health practice in a digital world: A clinician's guide. Cham, Switzerland: Springer International Publishing, Switzerland; pp. 159-181  Toombs E, Kowatch KR, Dalicandro L, McConkey S, Hopkins C, Mushquash CJ. 2020. A systematic  review of electronic mental health interventions for Indigenous youth: Results  and recommendations. *Journal of Telemedicine and Telecare.* **14:** 1357633X19899231.  Thomas RK, Suleman R, Mackay M, Hayer L, Singh M, Correll CU, Dursun S. 2020. Adapting to the impact of COVID-19 on mental health: an international perspective. *Journal of Psychiatry and Neuroscience* **45**: 229-233.  Tang S, Helmeste D. 2000. Digital psychiatry. *Psychiatry and Clinical Neurosciences.* **54:** 1-10.  Starling, J., Dossetor, D. 2005. Child and Adolescent Telepsychiatry. Wootton, Richard [Ed], Batch, Jennifer [Ed]. Telepediatrics: Telemedicine and child health. Lo: Royal Society of Medicine Press; pp. 77-87.  Spaulding, R., Cain, S., Sonnenschein, K. 2011. Urban telepsychiatry: Uncommon service for a common need. *Child and Adolescent Psychiatric Clinics of North America.* **20:** 29-39  Sousa A, Karia S. 2020. Telepsychiatry  during COVID-19: Some clinical, public health, and ethical dilemmas. *Indian Journal of Public Health.* **64:** S245-S246.  Moirangthem S, Rao S, Kumar CN, Narayana M, Raviprakash N, Math SB. 2017. Telepsychiatry as an Economically Better Model for Reaching the Unreached: A Retrospective Report from South India. *Indian Journal of Psychological Medicine.* **39:** 271-275.  Munro C.C., Hynan L.S, Grosch M, Parikh M, Weiner MF. Teleneuropsychology:  evidence for video teleconference-based neuropsychological assessment. *Journal of the International Neuropsychological Society. 2014.* **20:** 1028-33.  Myers K, Cain S. 2008. Practice parameter for telepsychiatry with children and  adolescents. *Journal of the American Academy of Child and Adolescent Psychiatry*. **47:** 1468-83.  Myers K, Nelson EL, Rabinowitz T, Hilty D, Baker D, Barnwell SS, Boyce G, Bufka LF, Cain S, Chui L, Comer JS, Cradock C, Goldstein F, Johnston B, Krupinski E, Lo K, Luxton DD, McSwain SD, McWilliams J, North S, Ostrowsky J, Pignatiello A, Roth D, Shore J, Turvey C, Varrell JR, Wright S, Bernard J. 2017. American  Telemedicine Association Practice Guidelines for Telemental Health with  Children and Adolescents. *Telemedicine and e-Health.* **23:** 779-804.  Naskar S, Victor R, Das H, Nath K. 2017. Telepsychiatry  in India - Where Do We Stand? A Comparative Review between Global and Indian  Telepsychiatry Programs. *Indian Journal of Psychological Medicine.* **39:** 223-242.  Norman S. 2006.The use of telemedicine in psychiatry. *Journal of Psychiatric and Mental Health Nursing.* **13:** 771-777.  O'Brien M, McNicholas F. The Use of Telepsychiatry During COVID-19 and Beyond. *Irish Journal of Psychological Medicine.* 2020. **21:** 1-17.  O'Keefe M, White K, Jennings JC. 2019. Asynchronous telepsychiatry: A systematic review. *Journal of Telemedicine and Telecare.* **29**: 1357633X19867189  Odor, A., Yellowlees, P., Hilty, D., Parish, M.B., Nafiz, N., Iosif, A. 2011. PsychVACS: A system for asynchronous telepsychiatry. *Telemedicine and e-Health*. **17:** 299-303  Paing WW, Weller RA, Welsh B, Foster T, Birnkrant JM, Weller EB. Telemedicine  in children and adolescents. *Current Psychiatric Reports. 2009.* **11:** 114-119.  Ramalho R, Adiukwu F, Gashi Bytyçi D, El Hayek S, Gonzalez-Diaz JM, Larnaout A, Grandinetti P, Kundadak GK, Nofal M, Pereira-Sanchez V, Pinto da Costa M, Ransing R, Schuh Teixeira AL, Shalbafan M, Soler-Vidal J, Syarif Z, Orsolini L. Telepsychiatry and healthcare access inequities during  the COVID-19 pandemic. 2020. *Asian Journal of Psychiatry.* **53:** 102234.  Report from the Alberta Heritage Foundation for Medical Research. 1998. Evaluation of a telepsychiatry pilot project. *International Journal of Technology Assessment in Health Care*. **14:** 583-4.  Rothchild, E. Telepsychiatry: Why do it? *Psychiatric Annals*. 1999. **29:** 394-401.  Sabin JE, Skimming K. A framework of ethics for telepsychiatry practice. *International Review of Psychiatry. 2015;* **27:** 490-495.  Samuels, A. 1999. International telepsychiatry: A link between New Zealand and Australia. *Australian and New Zealand Journal of Psychiatry*. **33:** 284-286.  Savin, D., Garry, M.T., Zuccaro, P., Novins, D. 2006. Telepsychiatry for treating rural American Indian Youth. *Journal of the American Academy of Child & Adolescent Psychiatry*. **45:** 484-488.  Seritan AL, Heiry M, Iosif AM, Dodge M, Ostrem JL. 2019. Telepsychiatry  for patients with movement disorders: a feasibility and patient satisfaction  study. *Journal of Clinical Movement Disorders.* **6:** 1.  Shore JH, Yellowlees P, Caudill R, Johnston B, Turvey C, Mishkind M, Krupinski E, Myers K, Shore P, Kaftarian E, Hilty D. 2018. Best Practices in Videoconferencing-Based Telemental Health April 2018. *Telemedicine and e-Health.* **24:** 827-832.  Shores, M.M., Ryan-Dykes, P., Williams, R.M., Mamerto, B., Sadak, T., Pascualy, M. et al. 2004. Identifying undiagnosed dementia in residential care veterans: comparing telemedicine to in-person clinical examination. *International Journal of Geriatric Psychiatry.* **19:** 101-108  Smith HA. 1999. Defending telepsychiatry. *Psychiatric Services.* **50:** 266-267. |
| Professional Guidance | Aboujaoude, E. Telemental health: Why the revolution has not arrived. *World Psychiatry.* 2018; **17:** 277-278  American Academy of Child and Adolescent Psychiatry (AACAP) Committee on Telepsychiatry and AACAP Committee on Quality Issues. 2017. Clinical update: Telepsychiatry with children and adolescents. *Journal of the American Academy of Child & Adolescent Psychiatry.* **56**: 875-893  Boydell KM, Hodgins M, Pignatiello A, Teshima J, Edwards H, Willis D. 2014. Using technology to deliver mental health services to children and youth: a scoping review. *Journal of the Canadian Academy of Child and Adolescent Psychiatry*. **23**:87-99.  Chipps J, Ramlall S, Mars M. 2012. Practice guidelines for videoconference-based telepsychiatry in South Africa. *African Journal of Psychiatry.* **15:** :271-82.  American Academy of Child and Adolescent Psychiatry (AACAP) Committee on Telepsychiatry and AACAP Committee on Quality Issues. 2017. Clinical Update: Telepsychiatry With Children and Adolescents. *Journal of the American Academy of Child & Adolescent Psychiatry.* **56:** 875-893  Ferrer DC, Yellowlees PM. 2012. Telepsychiatry: licensing and professional boundary  concerns. *Virtual Mentor*. **14**: 477-82.  Freudenberg N, Yellowlees PM. 2014. Telepsychiatry as Part of a Comprehensive Care Plan. *Virtual Mentor*. **16**: 964-8.  Gardner JS, Plaven BE, Yellowlees P, Shore JH. 2020. Remote Telepsychiatry Workforce: a Solution to Psychiatry's Workforce Issues. *Current Psychiatric Reports.* **22:** 8.  Grosch, M.C., Gottlieb, M.C., Cullum, C.M. Initial practice recommendations for teleneuropsychology. 2011. *The Clinical Neuropsychologist*. **25:** 1119-1133.  Hariman K, Ventriglio A, Bhugra D. 2019. The Future of Digital Psychiatry. *Current Psychiatry Reports.* **13:** 88.  Hilty DM, Luo, John S, Morache, Chris, Marcelo, Divine A & Nesbitt, Thomas S. 2002. Telepsychiatry: An overview for psychiatrists. *CNS Drugs.* **16:** 527-548.  Hilty DM, Marks, S.L., Urness, D., Yellowlees, P.M., Nesbitt, T.S.2004. Clinical and Educational Telepsychiatry Applications: A Review. *The Canadian Journal of Psychiatry / La Revue canadienne de psychiatrie*. **49:** 12-23.  Hilty DM, Yellowlees PM, Parrish MB, Chan S. 2015. Telepsychiatry: Effective, Evidence-Based, and at a Tipping Point in Health Care Delivery?. *Psychiatric Clinics of North America.* **38**: 559-92.  Hulsbosch, A.M., Nugter, M.A., Tamis, P., Kroon, H. 2017. Videoconferencing in a mental health service in The Netherlands: A randomized controlled trial on patient satisfaction and clinical outcomes for outpatients with severe mental illness. *Journal of Telemedicine and Telecare*. **23:** 513-520.  Jefee B.H., Mani N. International telepsychiatry: a review of what has been  published. *Journal of Telemedicine and Telecare*. **19:** 293-4.  Jones, Roland M, Leonard, S., Birmingham, L. 2006. Setting up a telepsychiatry service. *Psychiatric Bulletin*. **30:** 464-467  Lal, S., Abdel-Baki, A., Sujanani, S., Bourbeau, F., Sahed, I., Whitehead, J. 2020. Perspectives of young adults on receiving telepsychiatry services in an urban early intervention program for first-episode psychosis: A cross-sectional, descriptive survey study. *Frontiers in Psychiatry*. 11  Madhavan G. 2019. Telepsychiatry in intellectual disability psychiatry: literature  review. *BJPsych Bulletin.* **43**: 167-173  Malhotra S, Chakrabarti S, Shah R. 2013. Telepsychiatry:Promise, potential, and challenges. *Indian Journal of Psychiatry.* **55**: 3-11.  Math SB, Moirangthem S, Kumar NC. 2015. Tele-Psychiatry: After Mars, Can we Reach the Unreached?. *Indian Journal of Psychological Medicine.* **37:** 120-1.  McGrath J. 2020. ADHD and  Covid-19: Current roadblocks and future opportunities. *The Irish Journal of Psychological Medicine.* **21:** 1-22.  Clarke CS. 2018. Telepsychiatry in Asperger's syndrome. *Irish Journal of Psychological Medicine.* **35:** 325-328.  Whitten P, Kuwahara E. 2004. A multi-phase telepsychiatry programme in Michigan: organizational factors affecting utilization and user perceptions. *Journal of Telemedicine and Telecare.* **10:** 254-261.  Jones BN 3rd, Johnston D, Reboussin B, McCall WV. 2001. Reliability of telepsychiatry assessments: subjective versus observational ratings. *Journal of Geriatric Neurology and Psychiatry.* **14:** 66-71.  Saleem Y, Taylor MH, Khalifa N. 2008. Forensic telepsychiatry in the United Kingdom. *Behavioural Sciences and the Law*. **26:** 333-344.  Myers K. 2013. Telepsychiatry: time to connect. *Journal of the American Academy of Child and Adolescent Psychiatry.* **52:** 217-219.  Sharma A, Sasser T, Schoenfelder Gonzalez E, Vander Stoep A, Myers K.2020. Implementation of Home-Based Telemental Health in a Large Child Psychiatry Department During the COVID-19 Crisis. *Journal of Child and Adolescent Psychopharmacology.*  Frueh BC, Deitsch SE, Santos AB, Gold PB, Johnson MR, Meisler N, Magruder KM, Ballenger JC. 2000. Procedural and methodological issues in telepsychiatry research and program development. *Psychiatric Services.* **51:** 1522-1527.  Jones BN 3rd, Ruskin PE. 2001. Telemedicine and geriatric psychiatry: directions for future research and policy. *Journal of Geriatric Psychiatry and Neurology.* **14:** 59-62.  Monnier J, Knapp RG, Frueh BC. 2003. Recent advances in telepsychiatry: an updated review. *Psychiatric Services.* **54:** 1604-1609.  Hyler SE, Gangure DP. 2004. Legal and ethical challenges in telepsychiatry. *Journal of Psychiatric Practice.* **10:** 272-276.  Miller TW, Burton DC, Hill K, Luftman G, Veltkemp LJ, Swope M. 2005. Telepsychiatry: critical dimensions for forensic services. *Journal of Academic Psychiatry and Law.* **33:** 539-546.  Shore JH, Hilty DM, Yellowlees P. 2007. Emergency management guidelines for telepsychiatry. *General Hospital Psychiatry.* **29:** 199-206.  Pignatiello A, Teshima J, Boydell KM, Minden D, Volpe T, Braunberger PG. 2011. Child and youth telepsychiatry in rural and remote primary care. *Child and Adolescent Psychiatric Clinics in North America.* **20:** 13-28.  Shore JH. 2013. Telepsychiatry: videoconferencing in the delivery of psychiatric care. *American Journal of Psychiatry.* **170:** 256-262.  Salmoiraghi A, Hussain S. 2015. A Systematic Review of the Use of Telepsychiatry in Acute Settings. *Journal of Psychiatric Practice*. **21:** 389-393.  Nelson EL, Cain S, Sharp S. 2017. Considerations for Conducting Telemental Health with Children and Adolescents. *Child and Adolescent Psychiatric Clinics in North America.* **26:** 77-91.  Roth DE, Ramtekkar U, Zeković-Roth S. 2019. Telepsychiatry: A New Treatment Venue for Pediatric Depression. *Child and Adolescent Psychiatric Clinics in North America.* **28:** 377-395.  Smith K, Ostinelli E, Macdonald O, Cipriani A. 2020. COVID-19 and telepsychiatry: an evidence-based guidance for clinicians. *Journal of Medical Internet Research - International Scientific Journal for Medical Research.* **10.**  Fishkind, Avrim B & Cuyler, Robert N. 2013. The role of telepsychiatry. Zun, Leslie S [Ed], Chepenik, Lara G [Ed], Mallory, Mary Nan S [Ed]. Behavioral emergencies for the emergency physician. New York, NY, US: Cambridge University Press, US; pp. 303-307.  Yellowlees, Peter M, Hilty, Donald M & Mucic, Davor. 2016. Global/worldwide e-mental health: International and futuristic perspectives of telepsychiatry and the future. Mucic, Davor [Ed], Hilty, Donald M [Ed]. e-Mental health. Cham, Switzerland: Springer International Publishing, Switzerland; pp. 233-249  Vernig, P.M. 2016. Telemental health: Digital disruption and the opportunity to expand care. *Journal of the American Psychiatric Nurses Association*. **22:** 73-75.  Vanderpool, D. 2015. An overview of practicing high quality telepsychiatry. Dewan, Naakesh A [Ed], Luo, John S [Ed], Lorenzi, Nancy M [Ed]. Mental health practice in a digital world: A clinician's guide. Cham, Switzerland: Springer International Publishing, Switzerland; pp. 159-181  Valdagno M, Goracci A, di Volo S, Fagiolini A. 2014. Telepsychiatry: new perspectives and open issues. *CNS Spectrums.* **19:** 479-481.  Torous, J., Keshavan, M. & Gutheil, T. 2014. Promise and perils of digital psychiatry. *Asian Journal of Psychiatry.* **10:** 120-122.  Sunjaya AP, Chris A, Novianti D. 2020. Efficacy,  patient-doctor relationship, costs and benefits of utilizing telepsychiatry for  the management of post-traumatic stress disorder (PTSD): a systematic  review. *Trends in Psychiatry and Psychotherapy.* **42:** 102-110.  Starling, J., Dossetor, D. 2005. Child and Adolescent Telepsychiatry. Wootton, Richard [Ed], Batch, Jennifer [Ed]. Telepediatrics: Telemedicine and child health. Lo: Royal Society of Medicine Press; pp. 77-87.  Spaulding, R., Cain, S., Sonnenschein, K. 2011. Urban telepsychiatry: Uncommon service for a common need. *Child and Adolescent Psychiatric Clinics of North America.* **20:** 29-39  Sousa A, Karia S. 2020. Telepsychiatry  during COVID-19: Some clinical, public health, and ethical dilemmas. *Indian Journal of Public Health.* **64:** S245-S246.  Munro C.C., Hynan L.S, Grosch M, Parikh M, Weiner MF. 2014. Teleneuropsychology:  evidence for video teleconference-based neuropsychological assessment. *Journal of the International Neuropsychological Society.* **20:** 1028-33.  Myers K, Nelson EL, Rabinowitz T, Hilty D, Baker D, Barnwell SS, Boyce G, Bufka LF, Cain S, Chui L, Comer JS, Cradock C, Goldstein F, Johnston B, Krupinski E, Lo K, Luxton DD, McSwain SD, McWilliams J, North S, Ostrowsky J, Pignatiello A, Roth D, Shore J, Turvey C, Varrell JR, Wright S, Bernard J. 2017. American  Telemedicine Association Practice Guidelines for Telemental Health with  Children and Adolescents. *Telemedicine and e-Health.* **23:** 779-804.  Naskar S, Victor R, Das H, Nath K. Telepsychiatry in India - Where Do We Stand? A Comparative Review between Global and Indian  Telepsychiatry Programs. *Indian Journal of Psychological Medicine. 2017;* **39:** 223-242.  O'Brien M, McNicholas F. 2020. The Use of Telepsychiatry During COVID-19 and Beyond. *Irish Journal of Psychological Medicine.* **21:** 1-17.  Sales, C.P., McSweeney, L., Saleem, Y., Khalifa, N. 2018. The use of telepsychiatry within forensic practice: A literature review on the use of videolink-A ten-year follow-up. *Journal of Forensic Psychiatry & Psychology. 2018;*  **29:** 387-402.  Shore JH, Yellowlees P, Caudill R, Johnston B, Turvey C, Mishkind M, Krupinski E, Myers K, Shore P, Kaftarian E, Hilty D. 2018. Best Practices  in Videoconferencing-Based Telemental Health April 2018. *Telemedicine and e-Health.* **24:** 827-832. |

## *Appendix 2 Table 3: Table of Results*

| **Theme** | **Papers** |
| --- | --- |
| Diagnostic Reliability | Adaji A, Fortney J. 2017. Telepsychiatry in Integrated Care Settings. *Focus, The Journal of Lifelong Learning in Psychiatry.* **15:**257-263.  Amirsadri A, Burns J, Pizzuti A, Arfken CL. 2017. Home-Based Telepsychiatry in US Urban Area. *Case Reports in Psychiatry.* **2017:**6296423.  Balon R, Beresin EV, Coverdale JH, Louie AK, Roberts LW. Strengthening  telepsychiatry's role in clinical care and education. *Academic Psychiatry. 2015;*  **39**: 6-9  Bashshur RL, Shannon GW, Bashshur N, Yellowlees PM. 2016. The  Empirical Evidence for Telemedicine Interventions in Mental Disorders. *Telemedicine and e-Health.*  **22**:87-113  Bishop J.E., O’Reilly R.L., Maddox K., Hutchinson L.J. 2002. Client satisfaction in a feasibility study comparing face-to-face interviews with telepsychiatry. *Journal of telemedicine and telecare*. **8**: 217-221  Boydell KM, Hodgins M, Pignatiello A, Teshima J, Edwards H, Willis D. 2014. Using technology to deliver mental health services to children and youth: a scoping review. *Journal of the Canadian Academy of Child and Adolescent Psychiatry*. **23**:87-99.  Campbell R, O'Gorman J, Cernovsky ZZ. 2015. Reactions of Psychiatric Patients to Telepsychiatry. *Mental Illness Journal.* **7**: 6101.  Chakrabarti S. 2015. Usefulness of telepsychiatry: A critical evaluation of videoconferencing-based  approaches. *World Journal of Psychiatry.* **22:** 286-304.  Chipps J, Brysiewicz P, Mars M. 2012. Effectiveness and feasibility of telepsychiatry in resource constrained environments? A systematic review of the evidence. *African Journal of Psychiatry.* **15**: 235-43.  Chipps J, Ramlall S, Madigoe T, King H, Mars M. 2012. Developing telepsychiatry services in KwaZulu-Natal -- an action research study. *African Journal of Psychiatry*. **15:** 255-63.  De Las Cuevas C, A.J, De La Fuente J., Serrano P. 2003. Telepsychiatry in the Canary Islands: user acceptance and satisfaction. *Journal of Telemedicine and Telecare.* **9**: 221-4.  Deslich S, Stec B, Tomblin S, Coustasse A. 2013. Telepsychiatry  in the 21(st) century: transforming healthcare with technology. *Perspectives in Health Information Management.* **10:** 1f.  Diamond, J.M., Bloch, R.M. 2010. Telepsychiatry assessments of child or adolescent behavior disorders: A review of evidence and issues. *Telemedicine and e-Health.* **16:** 712-716  Gammon D, Bergvik S, Bergmo T, Pedersen S.1996. Videoconferencing  in psychiatry: a survey of use in northern Norway. *Journal of Telemedicine and Telecare.* **2:** 192-198.  Elford DR, White H, St John K, Maddigan B, Ghandi M, Bowering R. 2001. A prospective satisfaction study and cost analysis of a pilot child telepsychiatry service in Newfoundland. *Journal of Telemedicine and Telecare.* **7:** 73-81.  Elford R., White H., Bowering R., Ghandi A., Maddiggan B., St John K., House M., Harnett J., West R., Battcock A. 2000. A randomized, controlled trial of child psychiatric assessments conducted using videoconferencing. *Journal of telemedicine and telecare.* **6:** 73-82  Glueck DA. 2011. Telepsychiatry in private practice. *Child and Adolescent Psychiatric Clinics of North America*. **20**: 1-11.  Green AS, Ruchman SG, Katz CL, Singer EK. 2020. Piloting forensic tele-mental health evaluations of asylum seekers. *Psychiatry Research*. **291**: 113256.  Gunter, T.D, Srinivasaraghavan, J. Terry, N.P. 2003. Misinformed Regulation of Electronic Medicine Is Unfair to Responsible Telepsychiatry. *Journal of the American Academy of Psychiatry and the Law.* **31:** 10-14.  Grosch, M.C., Gottlieb, M.C., Cullum, C.M. 2011. Initial practice recommendations for teleneuropsychology. *The Clinical Neuropsychologist*. **25:** 1119-1133.  Hailey D, Jacobs P, Simpson J, Doze S. An  assessment framework for telemedicine applications. *Journal of telemedicine and telecare*. **5:** 162-70.  Hariman K, Ventriglio A, Bhugra D. 2019. The Future of Digital Psychiatry. *Current Psychiatry Reports.* **13:** 88.  Hilt, R.J. 2017. Telemedicine for child collaborative or integrated care. *Child and Adolescent Psychiatric Clinics of North America.* **26:** 637-645.  Hilty DM Johnston, B McCarron, R.M. 2016. How e-Mental health adds to traditional outpatient and newer models of integrated care for patients, providers, and systems. Mucic, Davor [Ed], Hilty, Donald M [Ed]. e-Mental health. Cham, Switzerland: Springer International Publishing, Switzerland; pp. 129-149.  Hilty DM, Bourgeois, J.A., Nesbitt, T.S., Hales, R.E. 2004. Cost issues with telepsychiatry in the United States. *Psychiatric Bulletin.* **28:** 6-8.  Hilty DM, Ferrer DC, Parish MB, Johnston B, Callahan EJ, Yellowlees PM. 2013. The effectiveness of telemental health. *Journal of telemedicine and telecare.* **19:** 444-454.  Hilty DM, Luo, John S, Morache, Chris, Marcelo, Divine A & Nesbitt, Thomas S. 2002. Telepsychiatry: An overview for psychiatrists. *CNS Drugs.* **16:** 527-548.  Hilty DM, Marks, S.L., Urness, D., Yellowlees, P.M., Nesbitt, T.S.2004. Clinical and Educational Telepsychiatry Applications: A Review. *The Canadian Journal of Psychiatry / La Revue canadienne de psychiatrie*. **49:** 12-23.  Hubley S, Lynch SB, Schneck C, Thomas M, Shore J. 2016. Review  of key telepsychiatry outcomes. *World Journal of Psychiatry*. **22:** 269-82  Jacob MK, Larson JC, Craighead WE. 2012. Establishing a telepsychiatry consultation practice in rural Georgia for primary care  physicians: a feasibility report. *Clinical Paediatrics.* **51:** 1041-1047.  Jones, B.N. 2002. Suicide among the elderly: The promise of telecommunications. *The British Journal of Psychiatry*. **181:** 191-192.  Kaftarian E. Lessons Learned in Prison and Jail-Based Telepsychiatry. *Current Psychiatric Reports*. **21:** 15.  Khalifa, N, Saleem, Y., Stankard, P. 2008. The use of telepsychiatry within forensic practice: A literature review on the use of videolink. *Journal of Forensic Psychiatry & Psychology.* **19:** 2-13.  Lau, M.E., Way, B.B., Fremont, W.P. 2011. Assessment of SUNY Upstate Medical University's child telepsychiatry consultation program. I*nternational Journal of Psychiatry in Medicine*. **42:** 93-104  Lee A, S.N., O'Connell F, Dyer A, Boniface K, Betz J. 2015. Telepsychiatric assessment of a mariner expressing suicidal ideation. *International Maritime Health.* **66**: 49-51.  Leonard S. 2004. The successes and challenges of developing a prison telepsychiatry service. *Journal of Telemedicine and Telecare*. **10**: 69-71.  Lexcen, F.J., Hawk, G.L., Herrick, S., Blank, M.B. 2006. Use of Video Conferencing for Psychiatric and Forensic Evaluations. *Psychiatric Services.* **57:** 713-715.  Litwack, S.D., Jackson, C.E., Chen, M., Sloan, D.M., Hatgis, C., Litz, B.T. et al. 2014. Validation of the use of video teleconferencing technology in the assessment of PTSD*. Psychological Services.* **11:** 290-294.  Loh, P. K, Maher, S., Goldswain, P., Flicker, L., Ramesh, P., Saligari, J. 2005. Diagnostic accuracy of telehealth community dementia assessments. *Journal of the American Geriatrics Society.* **53:** 2043-2044  Loh, P.K., Donaldson, M., Flicker, L., Maher, Sean S., Goldswain, P. 2007. Development of a telemedicine protocol for the diagnosis of Alzheimer's disease. *Journal of Telemedicine and Telecare.* **13:** 90-94.  Malhotra S, Chakrabarti S, Shah R. 2013. Telepsychiatry:Promise, potential, and challenges. *Indian Journal of Psychiatry.* **55**: 3-11.  Martin-Khan, M., Wootton, R., Whited, J., Gray, L.C. 2011. A systematic review of studies concerning observer agreement during medical specialist diagnosis using videoconferencing. *Journal of Telemedicine and Telecare*. **17:** 350-357.  Mazhari S, Ghaffari Nejad A, Mofakhami O, Raaii F, Bahaadinbeigy K. Evaluating  the Diagnostic Agreement between Telepsychiatry Assessment and Face-to-Face  Visit: A Preliminary Study. *Iranian Journal of Psychiatry*. 2019; **14:** 236-241.  Menon, A. Srikumar, K., Prasad, K., Popuri, Chrismer, J.B., Raskin, A., Hebel, J.R. et al. 2001. Evaluation of a portable low cost videophone system in the assessment of depressive symptoms and cognitive function in elderly medically ill veterans. *Journal of Nervous and Mental Disease*. **189:** 399-401.  Heravian A, Chang BP. 2018. Mental health and telemedicine in the acute care setting: Applications of telepsychiatry in the ED.*The American Journal of Emergency Medicine.* **36:** 1118-1119  Matsuura S, Hosaka T, Yukiyama T, Ogushi Y, Okada Y, Haruki Y, Nakamura M. 2000. Application of telepsychiatry: a preliminary study. *Psychiatry and Clinical Neurosciences.* **54:** 55-58.  Brodey BB, Claypoole KH, Motto J, Arias RG, Goss R. 2000. Satisfaction of forensic psychiatric patients with remote telepsychiatric evaluation. *Psychiatric Services.* **51:** 1305-1307.  Jones BN 3rd, Johnston D, Reboussin B, McCall WV. 2001. Reliability of telepsychiatry assessments: subjective versus observational ratings. *Journal of Geriatric Neurology and Psychiatry.* **14:** 66-71.  Rohland BM. 2001. Telepsychiatry in the heartland: if we build it, will they come?. *Community Mental Health Journal.* **37:** 449-459.  Shore JH, Savin D, Orton H, Beals J, Manson SM. 2007. Diagnostic reliability of telepsychiatry in American Indian veterans. *American Journal of Psychiatry.* **164:** 115-1118.  Singh SP, Arya D, Peters T. 2007. Accuracy of telepsychiatric assessment of new routine outpatient referrals. *BMC Psychiatry.* **7**: 55.  Myers KM, Palmer NB, Geyer JR. 2011. Research in child and adolescent telemental health. *Child and Adolescent Psychiatric Clinics in North America.* **20:** 155-171.  Savin D, Glueck DA, Chardavoyne J, Yager J, Novins DK. 2011. Bridging cultures: child psychiatry via videoconferencing. *Child and Adolescent Psychiatric Clinics in North America.* **20:** 125-134.  Myers K. Telepsychiatry: time to connect. *Journal of the American Academy of Child and Adolescent Psychiatry. 2013;* **52:** 217-219.  Nassan M, Frye MA, Adi A, Alarcón RD. 2015. Telepsychiatry for post-traumatic stress disorder: a call for action in the Syrian conflict. *Lancet Psychiatry*. **2:** 866.  Das S, Manjunatha N, Kumar CN, Math SB, Thirthalli J. 2020. Tele-psychiatric after care clinic for the continuity of care: A pilot study from an academic hospital. *Asian Journal of Psychiatry.* **48:** 101886.  Sharma A, Sasser T, Schoenfelder Gonzalez E, Vander Stoep A, Myers K.2020. Implementation of Home-Based Telemental Health in a Large Child Psychiatry Department During the COVID-19 Crisis. *Journal of Child and Adolescent Psychopharmacology.*  Frueh BC, Deitsch SE, Santos AB, Gold PB, Johnson MR, Meisler N, Magruder KM, Ballenger JC. 2000. Procedural and methodological issues in telepsychiatry research and program development. *Psychiatric Services.* **51:** 1522-1527.  Jones BN 3rd, Ruskin PE. 2001. Telemedicine and geriatric psychiatry: directions for future research and policy. *Journal of Geriatric Psychiatry and Neurology.* **14:** 59-62.  Monnier J, Knapp RG, Frueh BC. 2003. Recent advances in telepsychiatry: an updated review. *Psychiatric Services.* **54:** 1604-1609.  Modai I, Jabarin M, Kurs R, Barak P, Hanan I, Kitain L. 2007. Cost effectiveness, safety, and satisfaction with video telepsychiatry versus face-to-face care in ambulatory settings. *Telemedicine and e-Health*. **12:** 515-520.  Ramos-Ríos R, Mateos R, Lojo D, Conn DK, Patterson T. 2012. Telepsychogeriatrics: a new horizon in the care of mental health problems in the elderly. *International Psychogeriatrics.* **24:** 1708-1724.  Yellowlees P, Richard Chan S, Burke Parish M. 2015. The hybrid doctor-patient relationship in the age of technology - Telepsychiatry consultations and the use of virtual space. *International Review Psychiatry.* **27:** 476-489.  Fortney JC, Pyne JM, Turner EE, Farris KM, Normoyle TM, Avery MD, Hilty DM, Unützer J. 2015. Telepsychiatry integration of mental health services into rural primary care settings. *International Review Psychiatry.* **27:** 525-539.  Roth DE, Ramtekkar U, Zeković-Roth S. 2019. Telepsychiatry: A New Treatment Venue for Pediatric Depression. *Child and Adolescent Psychiatric Clinics in North America.* **28:** 377-395.  Farabee D., Calhoun S., Veliz R. 2016. An experimental comparison of telepsychiatry and conventional psychiatry for parolees. *Psychiatric services.* **67**: 562-565.  O’Reilly R., Bishop J., Maddox K., Hutchinson L., Fisman M., Takhar J. 2007. Is telepsychiatry equivalent to face-to-face psychiatry? Results from a randomized controlled equivalence trial. *Psychiatric Services*. **58:** 863-843  Fishkind, Avrim B & Cuyler, Robert N. 2013. The role of telepsychiatry. Zun, Leslie S [Ed], Chepenik, Lara G [Ed], Mallory, Mary Nan S [Ed]. Behavioral emergencies for the emergency physician. New York, NY, US: Cambridge University Press, US; pp. 303-307.  Alessi N. 2002. High-bandwidth interactive telepsychiatry. *Psychiatric Services.* **53:** 901-902.  Jones BN 3rd. 2001. Telepsychiatry and geriatric care. *Current Psychiatry Reports.* **3:** 29-36.  Zaylor C. 1999. Clinical outcomes in telepsychiatry. *Journal of Telemedicine and Telecare.* **5:** S59-60.  Zaylor C, Nelson EL, Cook DJ. 2001. Clinical outcomes in a prison telepsychiatry clinic. *Journal of Telemedicine and Telecare.* **1:** 47-49.  Yellowlees, Peter M, Hilty, Donald M, Marks, Shayna L, Neufeld, Jonathan & Bourgeois, James A. 2008. A retrospective analysis of a child and adolescent eMental Health program. *Journal of the American Academy of Child & Adolescent Psychiatry.* **47:** 103-107.  Work Group on Quality Issues (WGQI) & American Academy of Child and Adolescent Psychiatry (AACAP), US. 2008. Practice parameter for telepsychiatry with children and adolescents. *Journal of the American Academy of Child & Adolescent Psychiatry.* **47:** 1468-1483.  Volicer L. 2015. Nursing home telepsychiatry. *Journal of the American Medical Directors Association*. **16**: 7-8.  van Wynsberghe A, Gastmans C. 2009. Telepsychiatry and the meaning of in-person contact: a preliminary ethical appraisal. *Medicine, Health Care and Philosophy.* **12:** 469-476.  Sunjaya AP, Chris A, Novianti D. 2020. Efficacy,  patient-doctor relationship, costs and benefits of utilizing telepsychiatry for  the management of post-traumatic stress disorder (PTSD): a systematic  review. *Trends in Psychiatry and Psychotherapy.* **42:** 102-110.  Starling, J., Dossetor, D. 2005. Child and Adolescent Telepsychiatry. Wootton, Richard [Ed], Batch, Jennifer [Ed]. Telepediatrics: Telemedicine and child health. Lo: Royal Society of Medicine Press; pp. 77-87.  Munro C.C., Hynan L.S, Grosch M, Parikh M, Weiner MF. 2014. Teleneuropsychology:  evidence for video teleconference-based neuropsychological assessment. *Journal of the International Neuropsychological Society.* **20:** 1028-33.  Nelson EL, Zaylor C, Cook D. 2004. A comparison of psychiatrist evaluation and patient symptom report in a jail telepsychiatry  clinic. *Telemedicine and e-Health.* **2:**S54-59  Saeed SA, Anand V. 2015. Use of Telepsychiatry in Psychodynamic Psychiatry. *Psychodynamic Psychiatry*. **43**: 569-83.  Setterberg, S.R., Busseri, M.A., Fleissner, R.M., Kenney, E.M., Flom, J.A., Fischer, K.J. Remote assessment of the use of seclusion and restraint with paediatric psychiatric patients. *Journal of Telemedicine and Telecare. 2003;*  **9:** 176-179  Shores, M.M., Ryan-Dykes, P., Williams, R.M., Mamerto, B., Sadak, T., Pascualy, M. et al. 2004. Identifying undiagnosed dementia in residential care veterans: comparing telemedicine to in-person clinical examination. *International Journal of Geriatric Psychiatry.* **19:** 101-108 |
| Outcomes | Aboujaoude, E., Gega. 2020. From digital mental health interventions to digital "addiction": Where the two fields converge. *Frontiers in Psychiatry.* 10.  Abrams J, Sossong S, Schwamm LH, Barsanti L, Carter M, Kling N, Kotarski M, Leddy J, Meller B, Simoni M, Sullivan M, Wozniak J. 2017. Practical Issues in Delivery of Clinician-to-Patient Telemental Health in an Academic Medical Center. *Harvard Review of Psychiatry.* **25**:135-145.  Adaji A, Fortney J. 2017. Telepsychiatry in Integrated Care Settings. *Focus, The Journal of Lifelong Learning in Psychiatry.* **15:**257-263.  American Academy of Child and Adolescent Psychiatry (AACAP) Committee on Telepsychiatry and AACAP Committee on Quality Issues. 2017. Clinical update: Telepsychiatry with children and adolescents. *Journal of the American Academy of Child & Adolescent Psychiatry.* **56**: 875-893  Barrera-Valencia C, Benito-Devia AV, Vélez-Álvarez C, Figueroa-Barrera M, Franco-Idárraga SM. 2017. Cost-effectiveness  of Synchronous vs. Asynchronous Telepsychiatry in Prison Inmates With  Depression. *Rev Colomb Psiquiatr*. **46**: 65-73.  Bashshur RL, Shannon GW, Bashshur N, Yellowlees PM. 2016. The  Empirical Evidence for Telemedicine Interventions in Mental Disorders. *Telemedicine and e-Health.*  **22**: 87-113  Batastini, Ashley B, McDonald, Brendan R & Morgan, Robert D. 2013. Videoteleconferencing in forensic and correctional practice. Myers, Kathleen [Ed], Turvey, Carolyn L [Ed]. Telemental health: Clinical, technical, and administrative foundations for evidence-based practice. Amsterdam, Netherlands: Elsevier, Netherlands; pp. 251-271.  Behere PB, Mansharamani HD, Kumar K. 2017. Telepsychiatry: Reaching the unreached. *Indian Journal of Medical Research.* **146:** 150-152.  Bishop J.E., O’Reilly R.L., Maddox K., Hutchinson L.J. 2002. Client satisfaction in a feasibility study comparing face-to-face interviews with telepsychiatry. *Journal of telemedicine and telecare*. **8**: 217-221  Bahloul, H.J., Mani, N. 2013. International telepsychiatry: A review of what has been published. *Journal of Telemedicine and Telecare.* **19**: 293-294.  Bolle R.R., Trondsen M.V., Stensland G.Ø., Tjora A. 2018. Usefulness of  videoconferencing in psychiatric emergencies -- a qualitative study. *Health and Technology.*  **8:** 111-117.  Borders C.B. 2017. Realizing the Promises of Telepsychiatry in Special Populations. *Mental Illness Journal.* **9:** 7135.  Boydell KM, Hodgins M, Pignatiello A, Teshima J, Edwards H, Willis D. 2014. Using technology to deliver mental health services to children and youth: a scoping review. *Journal of the Canadian Academy of Child and Adolescent Psychiatry*. **23**:87-99.  Buist A, Coman G, Silvas A, Burrows G. 2000. An evaluation of the telepsychiatry programme in Victoria, Australia. *Journal of Telemedicine and Telecare.* **6**: 216-21.  Butterfield, A. 2018. Telepsychiatric evaluation and consultation in emergency care settings. *Child and Adolescent Psychiatric Clinics of North America.* **27:** 467-478.  Chakrabarti S. 2015. Usefulness of telepsychiatry: A critical evaluation of videoconferencing-based  approaches. *World Journal of Psychiatry.* **22:** 286-304.  Chan S, Parish M, Yellowlees P. 2015. Telepsychiatry Today.*Current Psychiatry Reports.* **17:** :89  Chan SR, Torous J, Hinton L, Yellowlees P. 2014. Mobile Tele-Mental Health: Increasing Applications and a Move to Hybrid Models of Care. *Healthcare (Basel)*. **6**: 220-33.  Cheng KM, Siu BW, Au Yeung CC, Chiang TP, So MH, Yeung MC. 2018. Telepsychiatry for stable Chinese psychiatric out-patients in custody in Hong Kong: a case-control pilot study. *Hong Kong Medical Journal.* **24:** 378-383.  Chipps J, Brysiewicz P, Mars M. 2012. Effectiveness and feasibility of telepsychiatry in resource constrained environments? A systematic review of the evidence. *African Journal of Psychiatry.* **15**: 235-43.  Caudill, R.L., Sager, Z. 2015. Institutionally based videoconferencing. *International Review of Psychiatry*. **27:** 496-503.  Chipps J, Ramlall S, Madigoe T, King H, Mars M. 2012. Developing telepsychiatry services in KwaZulu-Natal -- an action research study. *African Journal of Psychiatry*. **15:** 255-63.  Chong, J., Moreno, F. 2012. Feasibility and acceptability of clinic-based telepsychiatry for low-income Hispanic primary care patients. *Telemedicine and e-Health.* **18**: 297-304  American Academy of Child and Adolescent Psychiatry (AACAP) Committee on Telepsychiatry and AACAP Committee on Quality Issues. 2017. Clinical Update: Telepsychiatry With Children and Adolescents. *Journal of the American Academy of Child & Adolescent Psychiatry.* **56:** 875-893  Cowan KE, McKean AJ, Gentry MT, Hilty DM. 2019. Barriers to Use of Telepsychiatry: Clinicians as Gatekeepers. *Mayo Clinic Proceedings.* **94:** 2510-2523.  Crowe T, Jani S, Jani S, Jani N, Jani R. 2016. A pilot program in rural telepsychiatry for deaf and hard of hearing  populations. *Heliyon*. **2**: e00077  Deslich S, Stec B, Tomblin S, Coustasse A. Telepsychiatry in the 21(st) century: transforming healthcare with technology. *Perspectives in Health Information Management. 2013;* **10:** 1f.  Deslich SA, Thistlethwaite T, Coustasse A. 2013. Telepsychiatry  in correctional facilities: using technology to improve access and decrease  costs of mental health care in underserved populations. *The Permanente Journal* **17:** 80-86.  Detweiler MB, Arif S, Candelario J, Altman J, Murphy PF, Halling MH, Detweiler JG, Vasudeva S. 2011. A telepsychiatry transition clinic: the first 12 months  experience. *Journal of Telemedicine and Telecare.* **17:** 293-297.  Diamond, J.M., Bloch, R.M. 2010. Telepsychiatry assessments of child or adolescent behavior disorders: A review of evidence and issues. *Telemedicine and e-Health.* **16:** 712-716  Donley, E., McClaren, A., Jones, R., Katz, P., Goh, J. 2017. Evaluation and implementation of a telepsychiatry trial in the emergency department of a metropolitan public hospital. *Journal of Technology in Human Services*. **35:** 292-313.  Dossetor, D. R, Nunn, K. P, Fairley, M., Eggleton, D. 1999. A child and adolescent psychiatric outreach service for rural New South Wales: A telemedicine pilot study. *Journal of Paediatrics and Child Health*. **35:** 525-529.  Egede LE, Frueh CB, Richardson LK, Acierno R, Mauldin PD, Knapp RG, Lejuez C. 2009. Rationale and design: telepsychology service delivery for depressed elderly veterans. *Trials.* **10:** 22  Ellington, E., Repique, R., John R. 2013. Telemental health adoption can change psychiatric-mental health nursing practice. *Journal of the American Psychiatric Nurses Association.* **19:** 222-224  Flaum MA. 2017. When Will Telepsychiatry Reach Its "Tipping Point"?. *Psychiatric Services*. **68**:1205.  Freudenberg N, Yellowlees PM. 2014. Telepsychiatry as Part of a Comprehensive Care Plan. *Virtual Mentor*. **16**: 964-8.  Gardner JS, Plaven BE, Yellowlees P, Shore JH. 2020. Remote Telepsychiatry Workforce: a Solution to Psychiatry's Workforce Issues. *Current Psychiatric Reports.* **22:** 8.  Doze S, Simpson J, Hailey D, Jacobs P. 1999. Evaluation of a telepsychiatry pilot project. *Journal of Telemedicine and Telecare.* **5:** 38-46  Elford DR, White H, St John K, Maddigan B, Ghandi M, Bowering R. 2001. A prospective satisfaction study and cost analysis of a pilot child telepsychiatry service in Newfoundland. *Journal of Telemedicine and Telecare.* **7:** 73-81.  Elford R., White H., Bowering R., Ghandi A., Maddiggan B., St John K., House M., Harnett J., West R., Battcock A. 2000. A randomized, controlled trial of child psychiatric assessments conducted using videoconferencing. *Journal of telemedicine and telecare.* **6:** 73-82  Gentile, J.P, Cowan, A.E., Harper, B., Mast, R., Merrill, B. 2018. Reaching rural Ohio with intellectual disability psychiatry. *Journal of Telemedicine and Telecare.* **24**: 434-439  Gloff, N.E, LeNoue, S.R, Novins, D.K., Myers, K. 2015. Telemental health for children and adolescents. *International Review of Psychiatry*. **27:** 513-524.  Glueck DA. 2011. Telepsychiatry in private practice. *Child and Adolescent Psychiatric Clinics of North America*. **20**: 1-11.  Gopalan P, Shenai N, Dunn S, Bilderback A. 2020. Healthcare  utilization in patients pre-and post-telepsychiatry consultation compared to  in-person consultation-liaison sites. *General Hospital Psychiatry.* **67:** 154-155.  Gowda, Guru S, Kulkarni, Karishma, Bagewadi, Virupaksha, R. P. S., Shyam, Manjunatha, B. R, Shashidhara, Harihara N, et al. 2018. A study on collaborative telepsychiatric consultations to outpatients of district hospitals of Karnataka, India. *Asian Journal of Psychiatry.* **37:** 161-166  Grady B. 2012. Promises and limitations of telepsychiatry in rural adult mental health care. *World Psychiatry.* **11**: 199-201.  Haghnia Y., Samad-Soltani T., Yousefi M., Sadr H., Rezaei-Hachesu P. 2010. Telepsychiatry-based care for the treatment follow-up of Iranian war veterans with post-traumatic stress disorder: a randomized controlled trial. *Iranian journal of medical sciences*. **44**: 291-298.  Hariman K, Ventriglio A, Bhugra D. 2019. The Future of Digital Psychiatry. *Current Psychiatry Reports.* **13:** 88.  Hensel J, Graham R, Isaak C, Ahmed N, Sareen J, Bolton J. A Novel Emergency Telepsychiatry Program in a Canadian Urban Setting: Identifying and Addressing Perceived Barriers for Successful Implementation: Un nouveau programme de télépsychiatrie d'urgence en milieu urbain canadien: Identifier et aborder les obstacles perçus d'une mise en œuvre réussie. *Canadian Journal of Psychiatry; 2020* **65:** 559-567.  Hilt, R.J. 2017. Telemedicine for child collaborative or integrated care. *Child and Adolescent Psychiatric Clinics of North America.* **26:** 637-645.  Hilty DM Johnston, B McCarron, R.M. 2016. How e-Mental health adds to traditional outpatient and newer models of integrated care for patients, providers, and systems. Mucic, Davor [Ed], Hilty, Donald M [Ed]. e-Mental health. Cham, Switzerland: Springer International Publishing, Switzerland; pp. 129-149.  Hilty DM, Mucic, D. 2016. Technology, health, and contemporary practice: How does e-mental health fit it and what does it offer? Mucic, Davor [Ed], Hilty, Donald M [Ed]. e-Mental health. Cham, Switzerland: Springer International Publishing, Switzerland; pp. 3-27  Hilty DM, Bourgeois, J.A., Nesbitt, T.S., Hales, R.E. 2004. Cost issues with telepsychiatry in the United States. *Psychiatric Bulletin.* **28:** 6-8.  Hilty DM, Cobb, H.C., Neufeld, J.D., Bourgeois, J.A., Yellowlees, P.M. 2008. Telepsychiatry reduces geographic physician disparity in rural settings, but is it financially feasible because of reimbursement? *Psychiatric Clinics of North America*. **31:** 85-94  Hilty DM, Crawford A, Teshima J, Chan S, Sunderji N, Yellowlees PM, Kramer G, O'neill P, Fore C, Luo J, Li ST. 2015. A framework  for telepsychiatric training and e-health: Competency-based education, evaluation and implications. *International Review of Psychiatry.* **27:** 569-592.  Hilty DM, Ferrer DC, Parish MB, Johnston B, Callahan EJ, Yellowlees PM. 2013. The effectiveness of telemental health. *Journal of telemedicine and telecare.* **19:** 444-454.  Hilty DM, Luo, John S, Morache, Chris, Marcelo, Divine A & Nesbitt, Thomas S. 2002. Telepsychiatry: An overview for psychiatrists. *CNS Drugs.* **16:** 527-548.  Hilty DM, Marks, S.L., Urness, D., Yellowlees, P.M., Nesbitt, T.S.2004. Clinical and Educational Telepsychiatry Applications: A Review. *The Canadian Journal of Psychiatry / La Revue canadienne de psychiatrie*. **49:** 12-23.  Hilty DM, Sunderji, N., Suo, S., Chan, S., McCarron, R.M. 2018. Telepsychiatry and other technologies for integrated care: Evidence base, best practice models and competencies. *International Review of Psychiatry.* **30**: 292-309.  Hilty DM, Yellowlees PM, Parrish MB, Chan S. 2015. Telepsychiatry: Effective, Evidence-Based, and at a Tipping Point in Health Care Delivery?. *Psychiatric Clinics of North America.* **38**: 559-92.  Hubley S, Lynch SB, Schneck C, Thomas M, Shore J. 2016. Review  of key telepsychiatry outcomes. *World Journal of Psychiatry*. **22:** 269-82  Hungerbuehler I, Valiengo L, Loch AA, Rössler W, Gattaz WF. 2016. Home-Based  Psychiatric Outpatient Care Through Videoconferencing for Depression: A  Randomized Controlled Follow-Up Trial. *Journal of Medical Internet Research.* **3:** e36.  Jacob MK, Larson JC, Craighead WE. 2012. Establishing a telepsychiatry consultation practice in rural Georgia for primary care  physicians: a feasibility report. *Clinical Paediatrics.* **51:** 1041-1047.  Jefee B.H., Mani N. International telepsychiatry: a review of what has been  published. *Journal of Telemedicine and Telecare*. **19:** 293-4.  Jefee-Bahloul H. Telemental health in the middle East: overcoming the barriers. *Frontiers in Public Health*. 2014; **2:** 86  Kaftarian E. Lessons Learned in Prison and Jail-Based Telepsychiatry. *Current Psychiatric Reports*. **21:** 15.  Keilman, P. 2005. Telepsychiatry with Child Welfare Families Referred to a Family Service Agency. *Telemedicine and e-Health.* **11:** 98-101.  Kennedy C, Yellowlees P. 2000. A community-based approach to evaluation of health outcomes and costs for telepsychiatry in a rural population: preliminary results. *Journal of Telemedicine and Telecare*. **6:** S155-7.  Khalifa, N, Saleem, Y., Stankard, P. 2008. The use of telepsychiatry within forensic practice: A literature review on the use of videolink. *Journal of Forensic Psychiatry & Psychology.* **19:** 2-13.  Koblauch, H., Reinhardt, S.M., Lissau, W.J. & Jensen, P. 2018. The effect of telepsychiatric modalities on reduction of readmissions in psychiatric settings: A systematic review. *Journal of Telemedicine and Telecare.* **24:**, 31-36.  Kornbluh RA. Staying true to the mission: adapting telepsychiatry to a new environment. *CNS Spectrums*. 2014; **19**: 482-3.  Krzystanek M, Krysta K, Skałacka K. 2017. Treatment Compliance in the Long-Term Paranoid Schizophrenia Telemedicine Study. *Journal of Technology in Behavioural Science*. **2**: 84-87.  Krzystanek, M., Krzeszowski, D., Jagoda, K., Krysta, K. 2015. Long term telemedicine study of compliance in paranoid schizophrenia. *Psychiatria Danubina.* **27:** S266-S268.  LaBelle B, Franklyn AM, Pkh Nguyen V, Anderson KE, Eibl JK, Marsh DC. 2018. Characterizing the Use of Telepsychiatry for Patients with Opioid Use Disorder and Cooccurring Mental Health Disorders in Ontario, Canada. *International Journal of Telemedicine and Applications.* **2018:** 7937610.  Lal, S., Abdel-Baki, A., Sujanani, S., Bourbeau, F., Sahed, I., Whitehead, J. 2020. Perspectives of young adults on receiving telepsychiatry services in an urban early intervention program for first-episode psychosis: A cross-sectional, descriptive survey study. *Frontiers in Psychiatry*. 11  Lau, M.E., Way, B.B., Fremont, W.P. 2011. Assessment of SUNY Upstate Medical University's child telepsychiatry consultation program. I*nternational Journal of Psychiatry in Medicine*. **42:** 93-104  Madhavan G. 2019. Telepsychiatry in intellectual disability psychiatry: literature  review. *BJPsych Bulletin.* **43**: 167-173  Mahmoud H, Vogt EL, Dahdouh R, Raymond ML. 2020. Using Continuous Quality Improvement to Design and Implement a Telepsychiatry Program in Rural Illinois. *Psychiatric Services.* **15**: appips201900231.  Malhotra S, Chakrabarti S, Shah R. 2013. Telepsychiatry:Promise, potential, and challenges. *Indian Journal of Psychiatry.* **55**: 3-11.  Malhotra, S., Shah, R. 2018. Telepsychiatry and digital mental health care in child and adolescent psychiatry: Implications for service delivery in low- and middle-income countries. Hodes, Matthew [Ed], Shur-Fen Gau, Susan [Ed], De Vries, Petrus J [Ed]. Understanding uniqueness and diversity in child and adolescent mental health. San Diego, CA, US: Elsevier Academic Press, US; pp. 263-287  Math SB, Moirangthem S, Kumar NC. 2015. Tele-Psychiatry: After Mars, Can we Reach the Unreached?. *Indian Journal of Psychological Medicine.* **37:** 120-1.  Mazhari S, Ghaffari Nejad A, Mofakhami O, Raaii F, Bahaadinbeigy K. 2019. Evaluating  the Diagnostic Agreement between Telepsychiatry Assessment and Face-to-Face  Visit: A Preliminary Study. *Iranian Journal of Psychiatry*. **14:** 236-241.  McGrath J. ADHD and  Covid-19: Current roadblocks and future opportunities. *The Irish Journal of Psychological Medicine. 2020.* **21:** 1-22.  McLaren P. 2004. Telepsychiatry in Europe. *International Psychiatry.* **1**: 8-10.  Mettner J. The doctor is in another town: telepsychiatry brings care to people in rural Minnesota. *Minnesota Medicine*. 2013; **96**: 22-5.  Mielonen M, Ohinmaa A., Moring J & Isohanni M. Videoconferencing in telepsychiatry. *Journal of Technology in Human Services.* 2002; **20:** 183-199.  Clarke CS. 2018. Telepsychiatry in Asperger's syndrome. *Irish Journal of Psychological Medicine.* **35:** 325-328.  Ikelheimer, D.M. 2008. Treatment of opioid dependence via home- based telepsychiatry. *Psychiatric Services.* **59:** 1218-1219.  Benyakorn S. 2016. Implementing Telepsychiatry in Thailand Benefits and Challenges. *Journal of the Medical Association of Thailand*. **99**: S260-S266.  Hilty DM., Yellowlees, P.M. 2015. Collaborative mental health services using multiple technologies: The new way to practice and a new standard of practice? *Journal of the American Academy of Child & Adolescent Psychiatry.* **54:** 245-246.  Gabel S. 2009. Telepsychiatry, public mental health, and the workforce shortage in child and adolescent psychiatry. *Journal of the American Academy of Child & Adolescent Psychiatry.* **48:** 1127-1128.  Whitten P, Kuwahara E. 2004. A multi-phase telepsychiatry programme in Michigan: organizational factors affecting utilization and user perceptions. *Journal of Telemedicine and Telecare.* **10:** 254-261.  Narasimhan M, Druss BG, Hockenberry JM, Royer J, Weiss P, Glick G, Marcus SC, Magill J. 2015. Impact of a Telepsychiatry Program at Emergency Departments Statewide on the Quality, Utilization, and Costs of Mental Health Services. *Psychiatric Services.* **66:** 1167-72  Brodey BB, Claypoole KH, Motto J, Arias RG, Goss R. 2000. Satisfaction of forensic psychiatric patients with remote telepsychiatric evaluation. *Psychiatric Services.* **51:** 1305-1307.  Rohland BM. 2001. Telepsychiatry in the heartland: if we build it, will they come?. *Community Mental Health Journal.* **37:** 449-459.  Singh SP, Arya D, Peters T. 2007. Accuracy of telepsychiatric assessment of new routine outpatient referrals. *BMC Psychiatry.* **7**: 55.  Fox KC, Connor P, McCullers E, Waters T. 2008. Effect of a behavioural health and specialty care telemedicine programme on goal attainment for youths in juvenile detention. *Journal of Telemedicine and Telecare.* **14:** 227-230.  Myers KM, Palmer NB, Geyer JR. 2011. Research in child and adolescent telemental health. *Child and Adolescent Psychiatric Clinics in North America.* **20:** 155-171.  Szeftel R, Federico C, Hakak R, Szeftel Z, Jacobson M. 2012. Improved access to mental health evaluation for patients with developmental disabilities using telepsychiatry. *Journal of Telemedicine and Telecare.* **18:** 317-321.  Ulzen T, Williamson L, Foster PP, Parris-Barnes K. 2013. The evolution of a community-based telepsychiatry program in rural Alabama: lessons learned-a brief report. *Community Mental Health Journal.* **49:** 101-5.  Myers K. 2013. Telepsychiatry: time to connect. *Journal of the American Academy of Child and Adolescent Psychiatry.* **52:** 217-219.  Sharma A, Sasser T, Schoenfelder Gonzalez E, Vander Stoep A, Myers K.2020. Implementation of Home-Based Telemental Health in a Large Child Psychiatry Department During the COVID-19 Crisis. *Journal of Child and Adolescent Psychopharmacology.*  Frueh BC, Deitsch SE, Santos AB, Gold PB, Johnson MR, Meisler N, Magruder KM, Ballenger JC. 2000. Procedural and methodological issues in telepsychiatry research and program development. *Psychiatric Services.* **51:** 1522-1527.  Monnier J, Knapp RG, Frueh BC. 2003. Recent advances in telepsychiatry: an updated review. *Psychiatric Services.* **54:** 1604-1609.  McGinty KL, Saeed SA, Simmons SC, Yildirim Y. 2006. Telepsychiatry and e-mental health services: potential for improving access to mental health care. *Psychiatric Quarterly.* **77:** 335-342.  Urness D, Wass M, Gordon A, Tian E, Bulger T. 2006. Client acceptability and quality of life--telepsychiatry compared to in-person consultation. *Journal of Telemedicine and Telecare.* **12:** 251-254.  Modai I, Jabarin M, Kurs R, Barak P, Hanan I, Kitain L. 2007. Cost effectiveness, safety, and satisfaction with video telepsychiatry versus face-to-face care in ambulatory settings. *Telemedicine and e-Health*. **12:** 515-520.  Yellowlees P, Burke MM, Marks SL, Hilty DM, Shore JH. 2008. Emergency telepsychiatry. *Journal of Telemedicine and Telecare.* **14:** 277-281.  García-Lizana F, Muñoz-Mayorga I. 2010. What about telepsychiatry? A systematic review.*The Primary Care Companion to the Journal of Clinical Psychiatry.* **12.**  Myers KM, Vander Stoep A, McCarty CA, Klein JB, Palmer NB, Geyer JR, Melzer SM. 2010. Child and adolescent telepsychiatry: variations in utilization, referral patterns and practice trends. *Journal of Telemedicine and Telecare.* **16:** 128-133.  Szeftel R, Mandelbaum S, Sulman-Smith H, Naqvi S, Lawrence L, Szeftel Z, Coleman S, Gross L. 2011. Telepsychiatry for children with developmental disabilities: applications for patient care and medical education. *Child and Adolescent Psychiatric Clinics in North America.* **20:** 95-111.  Grady BJ, Lever N, Cunningham D, Stephan S. 2011. Telepsychiatry and school mental health. *Child and Adolescent Psychiatric Clinics in North America.* **20:** 81-94.  Shim R, Ye J, Yun K. 2012. Treating culturally and linguistically isolated Koreans via telepsychiatry. *Psychiatric Services.* **63:** 946.  Ramos-Ríos R, Mateos R, Lojo D, Conn DK, Patterson T. 2012. Telepsychogeriatrics: a new horizon in the care of mental health problems in the elderly. *International Psychogeriatrics.* **24:** 1708-1724.  Shore JH. 2013. Telepsychiatry: videoconferencing in the delivery of psychiatric care. *American Journal of Psychiatry.* **170:** 256-262.  Salmoiraghi A, Hussain S. 2015. A Systematic Review of the Use of Telepsychiatry in Acute Settings. *Journal of Psychiatric Practice*. **21:** 389-393.  Shore J. 2015. The evolution and history of telepsychiatry and its impact on psychiatric care: Current implications for psychiatrists and psychiatric organizations. *International Review of Psychiatry.* **27:** 469-475.  Yellowlees P, Richard Chan S, Burke Parish M. 2015. The hybrid doctor-patient relationship in the age of technology - Telepsychiatry consultations and the use of virtual space. *International Review Psychiatry.* **27:** 476-489.  Fortney JC, Pyne JM, Turner EE, Farris KM, Normoyle TM, Avery MD, Hilty DM, Unützer J. 2015. Telepsychiatry integration of mental health services into rural primary care settings. *International Review Psychiatry.* **27:** 525-539.  Lauckner C, Whitten P. 2016. The State and Sustainability of Telepsychiatry Programs. *The Journal of Behavioural Health Services and Research.* **43:** 305-318.  Nelson EL, Cain S, Sharp S. 2017. Considerations for Conducting Telemental Health with Children and Adolescents. *Child and Adolescent Psychiatric Clinics in North America.* **26:** 77-91.  Roberts N, Hu T, Axas N, Repetti L. 2017. Child and Adolescent Emergency and Urgent Mental Health Delivery Through Telepsychiatry: 12-Month Prospective Study. *Telemedicine and e-Health.* **23:** 842-846.  Swanson CL, Trestman RL. 2018. Rural Assertive Community Treatment and Telepsychiatry. *Journal of Psychiatric Practice.* **24:** 269-273.  Hassan A, Sharif K. 2019. Efficacy of Telepsychiatry in Refugee Populations: A Systematic Review of the Evidence. *Cureus.* **11**: e3984.  Smith K, Ostinelli E, Macdonald O, Cipriani A. 2020. COVID-19 and telepsychiatry: an evidence-based guidance for clinicians. *Journal of Medical Internet Research - International Scientific Journal for Medical Research.* **10.**  Myers K., Vander Stoep A., McCarty C.A., Katon W. 2015. Effectiveness of a telehealth service model for treating attention-deficit/hyperactivity disorder: a community-based randomized controlled trial. *Journal of the American Academy of Child and Adolescent Psychiatry.* **54:** 263-274.  Shulman M., John M., Kane J.M. 2017. Home-based outpatient telepsychiatry to improve adherence with treatment appointments: a pilot study. *Psychiatric services.* **68:** 743-746.  Farabee D., Calhoun S., Veliz R. 2016. An experimental comparison of telepsychiatry and conventional psychiatry for parolees. *Psychiatric services.* **67**: 562-565.  O’Reilly R., Bishop J., Maddox K., Hutchinson L., Fisman M., Takhar J. 2007. Is telepsychiatry equivalent to face-to-face psychiatry? Results from a randomized controlled equivalence trial. *Psychiatric Services*. **58:** 863-843  Fishkind, Avrim B & Cuyler, Robert N. 2013. The role of telepsychiatry. Zun, Leslie S [Ed], Chepenik, Lara G [Ed], Mallory, Mary Nan S [Ed]. Behavioral emergencies for the emergency physician. New York, NY, US: Cambridge University Press, US; pp. 303-307.  Saurman E, Lyle D, Perkins D, Roberts R. 2014. Successful provision of emergency mental health care to rural and remote New South Wales: an evaluation of the Mental Health Emergency Care-Rural Access Program. *Australian Health Review.* **38:** 58-64.  Mahmoud H, Vogt E. 2019. Telepsychiatry: an Innovative Approach to Addressing the Opioid Crisis. *Journal of Behavioural Health Sciences and Research.* **46:** 680-685.  Jones BN 3rd. 2001. Telepsychiatry and geriatric care. *Current Psychiatry Reports.* **3:** 29-36.  Zaylor C. 1999. Clinical outcomes in telepsychiatry. *Journal of Telemedicine and Telecare.* **5:** S59-60.  Yilmaz SK, Horn BP, Fore C, Bonham CA. 2019. An economic cost analysis of an expanding, multi-state behavioural telehealth  intervention. *Journal of Telemedicine and Telecare.* **25:** 353-364.  Yellowlees, Peter M, Hilty, Donald M, Marks, Shayna L, Neufeld, Jonathan & Bourgeois, James A. 2008. A retrospective analysis of a child and adolescent eMental Health program. *Journal of the American Academy of Child & Adolescent Psychiatry.* **47:** 103-107.  Yellowlees, Peter M, Hilty, Donald M & Mucic, Davor. 2016. Global/worldwide e-mental health: International and futuristic perspectives of telepsychiatry and the future. Mucic, Davor [Ed], Hilty, Donald M [Ed]. e-Mental health. Cham, Switzerland: Springer International Publishing, Switzerland; pp. 233-249  Work Group on Quality Issues (WGQI) & American Academy of Child and Adolescent Psychiatry (AACAP), US. 2008. Practice parameter for telepsychiatry with children and adolescents. *Journal of the American Academy of Child & Adolescent Psychiatry.* **47:** 1468-1483.  Wojtuszek, Magdalena, Kachnic, Justyna, Krysta, Krzysztof & Wutke, Joanna. 2015. Telepsychiatry in Polish patients' and doctors' opinion. *Psychiatria Danubina.* **27:** S379-S382.  Wallace, Duncan & Hodges, Samantha. 2018. Telepsychiatry in the Australian Defence Force: A success story. *Australasian Psychiatry*. **26:** 105-106.  Ventriglio, Antonio, T.J., Castaldelli-Maia, J. 2017. Telepsychiatry and social psychiatry. *International Journal of Social Psychiatry.* **63:** 387-388.  Vanderpool, D. 2015. An overview of practicing high quality telepsychiatry. Dewan, Naakesh A [Ed], Luo, John S [Ed], Lorenzi, Nancy M [Ed]. Mental health practice in a digital world: A clinician's guide. Cham, Switzerland: Springer International Publishing, Switzerland; pp. 159-181  Vander S.A.,Myers K. 2013. Methodology for conducting the children's attention-deficit hyperactivity disorder telemental health treatment study in multiple underserved communities. *Clinical Trials.* **10:** 949-958.  van Wynsberghe A, Gastmans C. 2009. Telepsychiatry and the meaning of in-person contact: a preliminary ethical appraisal. *Medicine, Health Care and Philosophy.* **12:** 469-476.  Valdagno M, Goracci A, di Volo S, Fagiolini A. 2014. Telepsychiatry: new perspectives and open issues. *CNS Spectrums.* **19:** 479-481.  Trondsen MV, Bolle SR, Stensland GØ, Tjora A. 2014. Video-confidence: a qualitative exploration of videoconferencing for psychiatric emergencies. *BC Health Service Research*. **14:** 544.  Thompson D.A., Leimig R., Gower G., Winsett R.P. 2009. Assessment of depressive symptoms during post-transplant follow-up care performed via telehealth. *Telemedicine journal and e-health*. **15:** 700-706.  Thomas RK, Suleman R, Mackay M, Hayer L, Singh M, Correll CU, Dursun S. 2020. Adapting to the impact of COVID-19 on mental health: an international perspective. *Journal of Psychiatry and Neuroscience* **45**: 229-233.  Thomas JF, Novins DK, Hosokawa PW, Olson CA, Hunter D, Brent AS, Frunzi G, Libby AM. 2018. The Use of  Telepsychiatry to Provide Cost-Efficient Care During Pediatric Mental Health Emergencies. *Psychiatric Services.* **1:** 161-168.  Thiele, J.S., Doarn, C.R., Shore, J.H. 2015. Locum tenens and telepsychiatry: Trends in psychiatric care. *Telemedicine and e-Health*. **21:** 510-513.  Sunjaya AP, Chris A, Novianti D. 2020. Efficacy,  patient-doctor relationship, costs and benefits of utilizing telepsychiatry for  the management of post-traumatic stress disorder (PTSD): a systematic  review. *Trends in Psychiatry and Psychotherapy.* **42:** 102-110.  Sulzbacher, S.,, Vallin, T., Waetzig, E.Z. 2006. Telepsychiatry improves paediatric behavioural health care in rural communities. *Journal of Telemedicine and Telecare.* **12:** 285-288  Starling, J., Dossetor, D. 2005. Child and Adolescent Telepsychiatry. Wootton, Richard [Ed], Batch, Jennifer [Ed]. Telepediatrics: Telemedicine and child health. Lo: Royal Society of Medicine Press; pp. 77-87.  Stankard, P., Younus, S. 2007. Forensic telepsychiatry. *Psychiatric Bulletin*. **31:** 155.  Spaulding, R., Cain, S., Sonnenschein, K. 2011. Urban telepsychiatry: Uncommon service for a common need. *Child and Adolescent Psychiatric Clinics of North America.* **20:** 29-39  Moirangthem S, Rao S, Kumar CN, Narayana M, Raviprakash N, Math SB. Telepsychiatry as an Economically Better Model for Reaching the Unreached: A Retrospective Report from South India. *Indian Journal of Psychological Medicine. 2017.*  **39:** 271-275.  Pelton D, Wangelin B, Tuerk P. 2015. Utilizing Telehealth to Support Treatment of Acute Stress Disorder in a Theater of War:  Prolonged Exposure via Clinical Videoconferencing. *Telemedicine and e-Health.* **21:** 382-387.  Reliford A, Adebanjo B. 2019. Use of Telepsychiatry in Pediatric Emergency Room to Decrease Length of Stay for Psychiatric Patients, Improve Resident On-Call Burden, and Reduce Factors Related to Physician Burnout. *Telemedicine and e-Health.* **25**: 828-832.  Rockhill, C.M., Tse, Y.J., Fesinmeyer, M.D., Garcia, J., & Myers, K. 2016. Telepsychiatrists' medication treatment strategies in the children's attention-deficit/hyperactivity disorder telemental health treatment study. *Journal of Child and Adolescent Psychopharmacology.* **26:** 662-671  Ruskin PE, Silver-Aylaian M, Kling MA, Reed SA, Bradham DD, Hebel JR, Barrett D, Knowles F 3rd, Hauser P. 2004. Treatment  outcomes in depression: comparison of remote treatment through telepsychiatry  to in-person treatment. *American Journal of Psychiatry.* **161:** 1471-1476.  Saeed SA, Anand V. 2015. Use of Telepsychiatry in Psychodynamic Psychiatry. *Psychodynamic Psychiatry*. **43**: 569-83.  McLaren P, Ahlbom J, Riley A, Mohammedali A, Denis M. 2002. The North Lewisham telepsychiatry project: beyond the pilot phase. *Journal of Telemedicine and Telecare.* **8:** 90-100.  Sales, C.P., McSweeney, L., Saleem, Y., Khalifa, N. 2018. The use of telepsychiatry within forensic practice: A literature review on the use of videolink-A ten-year follow-up. *Journal of Forensic Psychiatry & Psychology.* **29:** 387-402.  Setterberg, S.R., Busseri, M.A., Fleissner, R.M., Kenney, E.M., Flom, J.A., Fischer, K.J. 2003. Remote assessment of the use of seclusion and restraint with paediatric psychiatric patients. *Journal of Telemedicine and Telecare.* **9:** 176-179 |
| Patient & Clinician Satisfaction | 2011. A telepsychiatry solution for rural eastern Texas. Burke Center Mental Health Emergency Center, Lufkin, Texas. *Psychiatric Services*. **62**:1384-6.  Aadil M, Cosme RM, Forcen FE, Khan AR. 2017. A Call for Emergency Action: Telepsychiatry for Trauma Treatment Among Syrian Refugees. *Cureus*. 2017; **18**:e1578.  Abba-Aji, A. 2006. Telepsychiatry: A solution to clinical efficacy or cost effectiveness. *Irish Journal of Psychological Medicine.* **23:**84.  Abdi YA, Elmi JY. 2011. Internet based telepsychiatry: a pilot case in Somaliland. *Medicine, Conflict and Survival.* **27**:145-50.  Aboujaoude, E., Gega. 2020. From digital mental health interventions to digital "addiction": Where the two fields converge. *Frontiers in Psychiatry.* 10.  Agarwal PP, Manjunatha N, Gowda GS, Kumar MNG, Shanthaveeranna N, Kumar CN, Math SB. 2019. Collaborative Tele-Neuropsychiatry Consultation Services for Patients in Central Prisons. *Journal of Neurosciences in Rural Practice.* **10**:101-105.  Alexander J, Lattanzio A. Utility of telepsychiatry for Aboriginal Australians. *Australian & New Zealand Journal of Psychiatry. 2009;* **43:** 1185.  Amirsadri A, Burns J, Pizzuti A, Arfken CL. 2017. Home-Based Telepsychiatry in US Urban Area. *Case Reports in Psychiatry.* **2017:**6296423.  Bashshur RL, Shannon GW, Bashshur N, Yellowlees PM. 2016. The  Empirical Evidence for Telemedicine Interventions in Mental Disorders. *Telemedicine and e-Health.*  **22**:87-113  Batastini, Ashley B, McDonald, Brendan R & Morgan, Robert D. 2013. Videoteleconferencing in forensic and correctional practice. Myers, Kathleen [Ed], Turvey, Carolyn L [Ed]. Telemental health: Clinical, technical, and administrative foundations for evidence-based practice. Amsterdam, Netherlands: Elsevier, Netherlands; pp. 251-271.  Behere PB, Mansharamani HD, Kumar K. 2017. Telepsychiatry: Reaching the unreached. *Indian Journal of Medical Research.* **146:** 150-152.  Ben-Zeev D. 2020. The Digital Mental Health Genie Is Out of the Bottle. *Psychiatric Services*. **24**:appips202000306.  Bishop J.E., O’Reilly R.L., Maddox K., Hutchinson L.J. 2002. Client satisfaction in a feasibility study comparing face-to-face interviews with telepsychiatry. *Journal of telemedicine and telecare*. **8**: 217-221  Bahloul, H.J., Mani, N. 2013. International telepsychiatry: A review of what has been published. *Journal of Telemedicine and Telecare.* **19**: 293-294.  Blackmon, L.A., Kaak, H.O., Ranseen, J. 1997. Consumer satisfaction with telemedicine child psychiatry consultation in rural Kentucky. *Psychiatric Services.* **48**: 1464-1466  Bolle R.R., Trondsen M.V., Stensland G.Ø., Tjora A. 2018. Usefulness of  videoconferencing in psychiatric emergencies -- a qualitative study. *Health and Technology.*  **8:** 111-117.  Borders C.B. 2017. Realizing the Promises of Telepsychiatry in Special Populations. *Mental Illness Journal.* **9:** 7135.  Boydell KM, Hodgins M, Pignatiello A, Teshima J, Edwards H, Willis D. 2014. Using technology to deliver mental health services to children and youth: a scoping review. *Journal of the Canadian Academy of Child and Adolescent Psychiatry*. **23**:87-99.  Boydell KM, Volpe T, Pignatiello A. 2010. A qualitative study of young people's perspectives on receiving psychiatric  services via televideo. *Journal of the Canadian Academy of Child and Adolescent Psychiatry*. **19**:5-11.  Boydell, K.M., Volpe, T., Kertes, A., Greenberg, N. 2007. A review of the outcomes of the recommendations made during paediatric telepsychiatry consultations. *Journal of Telemedicine and Telecare.* **13:** 277-281.  Buist A, Coman G, Silvas A, Burrows G. 2000. An evaluation of the telepsychiatry programme in Victoria, Australia. *Journal of Telemedicine and Telecare.* **6**: 216-21.  Butterfield, A. 2018. Telepsychiatric evaluation and consultation in emergency care settings. *Child and Adolescent Psychiatric Clinics of North America.* **27:** 467-478.  Campbell R, O'Gorman J, Cernovsky ZZ. 2015. Reactions of Psychiatric Patients to Telepsychiatry. *Mental Illness Journal.* **7**: 6101.  Rachal, J., Sparks, W., Zazzaro C., Blackwell T. 2015. Highlights in Telepsychiatry and Behavioural Health Emergencies.. *Psychiatric Clinics of North America.* **40:** 585-596  Cerda, G.M., Hilty, D.M., Hales, R.E., Nesbitt, T.S. 1999. Use of telemedicine with ethnic groups. *Psychiatric Services.* **50:** 1364.  Chakrabarti S. 2015. Usefulness of telepsychiatry: A critical evaluation of videoconferencing-based  approaches. *World Journal of Psychiatry.* **22:** 286-304.  Chan S, Parish M, Yellowlees P. 2015. Telepsychiatry Today.*Current Psychiatry Reports.* **17:** 89  Cheng KM, Siu BW, Au Yeung CC, Chiang TP, So MH, Yeung MC. 2018. Telepsychiatry for stable Chinese psychiatric out-patients in custody in Hong Kong: a case-control pilot study. *Hong Kong Medical Journal.* **24:** 378-383.  Chipps J, Brysiewicz P, Mars M. 2012. Effectiveness and feasibility of telepsychiatry in resource constrained environments? A systematic review of the evidence. *African Journal of Psychiatry.* **15**: 235-43.  Caudill, R.L., Sager, Z. 2015. Institutionally based videoconferencing. *International Review of Psychiatry*. **27:** 496-503.  Chipps J, Ramlall S, Madigoe T, King H, Mars M. Developing telepsychiatry services in KwaZulu-Natal -- an action research study. *African Journal of Psychiatry*. 2012. **15:** 255-63.  Chipps J, Ramlall S, Mars M. 2012. Practice guidelines for videoconference-based telepsychiatry in South Africa. *African Journal of Psychiatry.* **15:** :271-82.  Chong, J., Moreno, F. 2012. Feasibility and acceptability of clinic-based telepsychiatry for low-income Hispanic primary care patients. *Telemedicine and e-Health.* **18**: 297-304  American Academy of Child and Adolescent Psychiatry (AACAP) Committee on Telepsychiatry and AACAP Committee on Quality Issues. 2017. Clinical Update: Telepsychiatry With Children and Adolescents. *Journal of the American Academy of Child & Adolescent Psychiatry.* **56:** 875-893  Corruble E. 2020. A Viewpoint From Paris on the COVID-19 Pandemic: A Necessary Turn to Telepsychiatry. *Journal of Clinical Psychiatry.* **31:** 81  Cowan KE, McKean AJ, Gentry MT, Hilty DM. 2019. Barriers to Use of Telepsychiatry: Clinicians as Gatekeepers. *Mayo Clinic Proceedings.* **94:** 2510-2523.  Crowe T, Jani S, Jani S, Jani N, Jani R. 2016. A pilot program in rural telepsychiatry for deaf and hard of hearing  populations. *Heliyon*. **2**: e00077  Cruz, M.K., Elizabeth A., Lopez, A.M., Weinstein, R.S. 2005. A review of the first five years of the University of Arizona telepsychiatry programme. *Journal of Telemedicine and Telecare.* **11:** 234-239  De Las Cuevas C, A.J, De La Fuente J., Serrano P. 2003. Telepsychiatry in the Canary Islands: user acceptance and satisfaction. *Journal of Telemedicine and Telecare.* **9**: 221-4.  Deslich S, Stec B, Tomblin S, Coustasse A. 2013. Telepsychiatry  in the 21(st) century: transforming healthcare with technology. *Perspectives in Health Information Management.* **10:** 1f.  Deslich SA, Thistlethwaite T, Coustasse A. 2013. Telepsychiatry  in correctional facilities: using technology to improve access and decrease  costs of mental health care in underserved populations. *The Permanente Journal* **17:** 80-86.  Detweiler MB, Arif S, Candelario J, Altman J, Murphy PF, Halling MH, Detweiler JG, Vasudeva S. 2011. A telepsychiatry transition clinic: the first 12 months  experience. *Journal of Telemedicine and Telecare.* **17:** 293-297.  Dham, P., Gupta, N., Alexander, J., Black, W., Rajji, T., Skinner, E. 2018. Community based telepsychiatry service for older adults residing in a rural and remote region- utilization pattern and satisfaction among stakeholders. *BMC Psychiatry.* 18.  Diamond, J.M., Bloch, R.M. 2010. Telepsychiatry assessments of child or adolescent behavior disorders: A review of evidence and issues. *Telemedicine and e-Health.* **16:** 712-716  Donley, E., McClaren, A., Jones, R., Katz, P., Goh, J. 2017. Evaluation and implementation of a telepsychiatry trial in the emergency department of a metropolitan public hospital. *Journal of Technology in Human Services*. **35:** 292-313.  Dossetor, D. R, Nunn, K. P, Fairley, M., Eggleton, D. 1999. A child and adolescent psychiatric outreach service for rural New South Wales: A telemedicine pilot study. *Journal of Paediatrics and Child Health*. **35:** 525-529.  Egede LE, Frueh CB, Richardson LK, Acierno R, Mauldin PD, Knapp RG, Lejuez C. 2009. Rationale and design: telepsychology service delivery for depressed elderly veterans. *Trials.* **10:** 22  Ermer D.J. 1999. Experience with a rural telepsychiatry clinic for children and adolescents. *Psychiatric Services.* **50:** 260-261.  Gammon D, Bergvik S, Bergmo T, Pedersen S.1996. Videoconferencing  in psychiatry: a survey of use in northern Norway. *Journal of Telemedicine and Telecare.* **2:** 192-198.  Gardner JS, Plaven BE, Yellowlees P, Shore JH. 2020. Remote Telepsychiatry Workforce: a Solution to Psychiatry's Workforce Issues. *Current Psychiatric Reports.* **22:** 8.  Gibson, K., O'Donnell, S., Coulson, H., Kakepetum-Schultz, T. 2011. Mental health professionals' perspectives of telemental health with remote and rural First Nations communities. *Journal of Telemedicine and Telecare.* **17:** 263-267.  Doze S, Simpson J, Hailey D, Jacobs P. 1999. Evaluation of a telepsychiatry pilot project. *Journal of Telemedicine and Telecare.* **5:** 38-46  Elford DR, White H, St John K, Maddigan B, Ghandi M, Bowering R. 2001. A prospective satisfaction study and cost analysis of a pilot child telepsychiatry service in Newfoundland. *Journal of Telemedicine and Telecare.* **7:** 73-81.  Gelber H. 2001. The experience in Victoria with telepsychiatry for the child and adolescent mental health service. *Journal of Telemedicine and Telecare.* **7:** 32-4.  Elford R., White H., Bowering R., Ghandi A., Maddiggan B., St John K., House M., Harnett J., West R., Battcock A. 2000. A randomized, controlled trial of child psychiatric assessments conducted using videoconferencing. *Journal of telemedicine and telecare.* **6:** 73-82  Gloff, N.E, LeNoue, S.R, Novins, D.K., Myers, K. 2015. Telemental health for children and adolescents. *International Review of Psychiatry*. **27:** 513-524.  Glueck DA. 2011. Telepsychiatry in private practice. *Child and Adolescent Psychiatric Clinics of North America*. **20**: 1-11.  Gowda, Guru S, Kulkarni, Karishma, Bagewadi, Virupaksha, R. P. S., Shyam, Manjunatha, B. R, Shashidhara, Harihara N, et al. 2018. A study on collaborative telepsychiatric consultations to outpatients of district hospitals of Karnataka, India. *Asian Journal of Psychiatry.* **37:** 161-166  Gratzer D, Torous J, Lam RW, Patten SB, Kutcher S, Chan S, Vigo D, Pajer K, Yatham LN. 2020. Our Digital Moment: Innovations and Opportunities in  Digital Mental Health Care. *The Canadian Journal of Psychiatry.*  Grubaugh, Anouk L, Cain, Gregory D, Elhai, Jon D, Patrick, Sarah L & Frueh, B. Christopher. 2008. Attitudes toward medical and mental health care delivered via telehealth applications among rural and urban primary care patients. *Journal of Nervous and Mental Disease.* **19:** 166-170.  Gunter, T.D, Srinivasaraghavan, J. Terry, N.P. 2003. Misinformed Regulation of Electronic Medicine Is Unfair to Responsible Telepsychiatry. *Journal of the American Academy of Psychiatry and the Law.* **31:** 10-14.  Haghnia Y., Samad-Soltani T., Yousefi M., Sadr H., Rezaei-Hachesu P. 2010. Telepsychiatry-based care for the treatment follow-up of Iranian war veterans with post-traumatic stress disorder: a randomized controlled trial. *Iranian journal of medical sciences*. **44**: 291-298.  Graham, M.A. 1996. Telepsychiatry in Appalachia. *American Behavioral Scientist*. **39:** 602-615.  Hailey D, Jacobs P, Simpson J, Doze S. 1999. An assessment framework for telemedicine applications. *Journal of telemedicine and telecare.* **5**:162-70.  Hariman K, Ventriglio A, Bhugra D. 2019. The Future of Digital Psychiatry. *Current Psychiatry Reports.* **13:** 88.  Hariman K, Ventriglio A, Bhugra D. 2019. The Future of Digital Psychiatry. *Current Psychiatry Reports.* **13:** 88.  Harley, J., McLaren, P., Blackwood, G., Tierney, K., Everett, M. 2002. The use of videoconferencing to enhance tertiary mental health service provision to the Island of Jersey. *Journal of Telemedicine and Telecare*. **8:** 36-38.  Harley, J. 2006. Economic evaluation of a tertiary telepsychiatry service to an island. *Journal of Telemedicine and Telecare*. **12:** 354-357.  Hasselberg MJ. 2020. The Digital Revolution in Behavioral Health. *Journal of the American Psychiatric Nurses Association.*  **26:** 102-111.  Hensel J, Graham R, Isaak C, Ahmed N, Sareen J, Bolton J. 2020. A Novel Emergency Telepsychiatry Program in a Canadian Urban Setting: Identifying and Addressing Perceived Barriers for Successful Implementation: Un nouveau programme de télépsychiatrie d'urgence en milieu urbain canadien: Identifier et aborder les obstacles perçus d'une mise en œuvre réussie. *Canadian Journal of Psychiatry.* **65:** 559-567.  Hilty DM, Crawford A, Teshima J, Chan S, Sunderji N, Yellowlees PM, Kramer G, O'neill P, Fore C, Luo J, Li ST. 2015. A framework  for telepsychiatric training and e-health: Competency-based education, evaluation and implications. *International Review of Psychiatry.* **27:** 569-592.  Hilty DM, Ferrer DC, Parish MB, Johnston B, Callahan EJ, Yellowlees PM. 2013. The effectiveness of telemental health. *Journal of telemedicine and telecare.* **19:** 444-454.  Hilty DM, Gentry MT, McKean AJ, Cowan KE, Lim RF, Lu FG. 2020. Telehealth for rural diverse populations: telebehavioral and cultural competencies, clinical outcomes and administrative approaches. *mHealth Journal*. **6**: 20  Hilty DM, Luo JS, Morache C, Marcelo, Divine A & Nesbitt, TS. 2002. Telepsychiatry: An overview for psychiatrists. *CNS Drugs.* **16:** 527-548.  Hilty DM, Yellowlees PM, Parrish MB, Chan S. 2015. Telepsychiatry: Effective, Evidence-Based, and at a Tipping Point in Health Care Delivery?. *Psychiatric Clinics of North America.* **38**: 559-92.  Hockey AD, Yellowlees PM, Murphy S. 2004. Evaluation  of a pilot second-opinion child telepsychiatry service. *Journal of telemedicine and telecare*. **10**: 48-50.  Hubley S, Lynch SB, Schneck C, Thomas M, Shore J. 2016. Review  of key telepsychiatry outcomes. *World Journal of Psychiatry*. **22:** 269-82  Hulsbosch, A.M., Nugter, M.A., Tamis, P., Kroon, H. 2017. Videoconferencing in a mental health service in The Netherlands: A randomized controlled trial on patient satisfaction and clinical outcomes for outpatients with severe mental illness. *Journal of Telemedicine and Telecare*. **23:** 513-520.  Hungerbuehler I, Valiengo L, Loch AA, Rössler W, Gattaz WF. 2016. Home-Based  Psychiatric Outpatient Care Through Videoconferencing for Depression: A  Randomized Controlled Follow-Up Trial. *Journal of Medical Internet Research.* **3:** e36.  Hungerbuehler I., Leite R.F.M., van de Bilt M.T., Gattaz W.F. 2015. A randomized clinical trial of home-based telepsychiatric outpatient care via videoconferencing: design, methodology, and implementation. *Revista de psiquiatria clinica.* **42**: 76-78.  Jacob MK, Larson JC, Craighead WE. 2012. Establishing a telepsychiatry consultation practice in rural Georgia for primary care  physicians: a feasibility report. *Clinical Paediatrics.* **51:** 1041-1047.  Jefee B.H., Mani N. International telepsychiatry: a review of what has been published. *Journal* Telemed Telecare. 2013; **19:** 293-4.  Jefee-Bahloul H. 2014. Telemental health in the middle East: overcoming the barriers. *Frontiers in Public Health*. **2:** 86  Jones, Roland M, Leonard, S., Birmingham, L. Setting up a telepsychiatry service. *Psychiatric Bulletin*. 2006; **30:** 464-467  Kaftarian E. Lessons Learned in Prison and Jail-Based Telepsychiatry. *Current Psychiatric Reports*. **21:** 15.  Kalin ML, Garlow SJ, Thertus K, Peterson MJ. Rapid Implementation of Telehealth in Hospital Psychiatry in Response to COVID-19. *American Journal of Psychiatry.* **177**: 636-637.  Karlinsky H. 2004. Psychiatry, Technology, and the Corn Fields of Iowa. *The Canadian Journal of Psychiatry / La Revue canadienne de psychiatri*e, **49:** 1-3.  Katz CL, Washington FB, Sacco M, Schuetz-Mueller J. A Resident-Based Telepsychiatry Supervision Pilot Program in Liberia. *Psychiatric Services.* **70:** 243-246.  Kavanagh S, Hawker F. 2001. The fall and rise of the South Australian telepsychiatry network. *Journal of Telemedicine and Telecare*. **7**: 41-3.  Keilman, P. 2005. Telepsychiatry with Child Welfare Families Referred to a Family Service Agency. *Telemedicine and e-Health.* **11:** 98-101.  Kennedy C, Yellowlees P. 2000. A community-based approach to evaluation of health outcomes and costs for telepsychiatry in a rural population: preliminary results. *Journal of Telemedicine and Telecare*. **6:** S155-7.  Khalifa, N, Saleem, Y., Stankard, P. 2008. The use of telepsychiatry within forensic practice: A literature review on the use of videolink. *Journal of Forensic Psychiatry & Psychology.* **19:** 2-13.  Kimmel, R.J., Toor, R. 2019. Telepsychiatry by a public, academic medical center for inpatient consults at an unaffiliated, community hospital. Psychosomatics: *Journal of Consultation and Liaison Psychiatry*. **60**: 468-473.  Kopel H, Nunn K, Dossetor D. 2001. Evaluating satisfaction with a child and adolescent psychological telemedicine outreach  service. *Journal of Telemedicine and Telecare*. **7**: 35-40.  Kornbluh RA. 2014. Staying true to the mission: adapting telepsychiatry to a new environment. *CNS Spectrums*. **19**: 482-3.  LaBelle B, Franklyn AM, Pkh Nguyen V, Anderson KE, Eibl JK, Marsh DC. Characterizing the Use of Telepsychiatry for Patients with Opioid Use Disorder and Cooccurring Mental Health Disorders in Ontario, Canada. *International Journal of Telemedicine and Applications.* **2018:** 7937610.  Lal, S., Abdel-Baki, A., Sujanani, S., Bourbeau, F., Sahed, I., Whitehead, J. 2020. Perspectives of young adults on receiving telepsychiatry services in an urban early intervention program for first-episode psychosis: A cross-sectional, descriptive survey study. *Frontiers in Psychiatry*. 11  Lau, M.E., Way, B.B., Fremont, W.P. 2011. Assessment of SUNY Upstate Medical University's child telepsychiatry consultation program. I*nternational Journal of Psychiatry in Medicine*. **42:** 93-104  Lin, C., Bai, Y., Chen, J. 2003. Reliability of Information Provided by Patients of a Virtual Psychiatric Clinic. *Psychiatric Services.* **54:** 1167-1168.  Lingley-Pottie, P., McGrath, P.J. 2008. Telehealth: A child and family-friendly approach to mental health-care reform. *Journal of Telemedicine and Telecare*. **14:** 225-226.  Litwack S.D, Jackson C.E, Chen M, Sloan D.M, Hatgis C, Litz, B.T. & Marx B.P.. 2014. Validation of the use of video teleconferencing technology in the assessment of PTSD*. Psychological Services.* 2014; **11:** 290-294.  Madhavan G. 2019. Telepsychiatry in intellectual disability psychiatry: literature  review. *BJPsych Bulletin.* **43**: 167-173  Malhotra S, Chakrabarti S, Shah R. 2013. Telepsychiatry:Promise, potential, and challenges. *Indian Journal of Psychiatry.* **55**: 3-11.  Malhotra, S., Shah, R. 2018. Telepsychiatry and digital mental health care in child and adolescent psychiatry: Implications for service delivery in low- and middle-income countries. Hodes, Matthew [Ed], Shur-Fen Gau, Susan [Ed], De Vries, Petrus J [Ed]. Understanding uniqueness and diversity in child and adolescent mental health. San Diego, CA, US: Elsevier Academic Press, US; pp. 263-287  Mannion, L, Fahy, T. J, Duffy, C, Broderick, M., Gethins, E. 1998. 'Telepsychiatry': Keeping a link with an island. *Psychiatric Bulletin.* **22:** 47-49.  May CR, Ellis NT, Atkinson T, Gask L, Mair F, Smith C. 1999. Psychiatry by videophone:  a trial service in north west England. *Studies in Health Technology and Informatics*. **68**: 207-10.  Mazhari S, Ghaffari Nejad A, Mofakhami O, Raaii F, Bahaadinbeigy K. 2019. Evaluating  the Diagnostic Agreement between Telepsychiatry Assessment and Face-to-Face  Visit: A Preliminary Study. *Iranian Journal of Psychiatry*. **14:** 236-241.  McCann RA, Erickson JM, Palm-Cruz KJ. 2020. The  Development, Implementation, and Evaluation of a Novel Telepsychiatry  Curriculum for Integrated Care Psychiatry Fellows. *Academic Psychiatry.* **44:** 451-454.  McLaren P. 2004. Telepsychiatry in Europe. *International Psychiatry.* **1**: 8-10.  Meltzer, B. 1997. Telemedicine in emergency psychiatry. *Psychiatric Services.* **48:** 1141-1142.  Menon, A. Srikumar, K., Prasad, K., Popuri, Chrismer, J.B., Raskin, A., Hebel, J.R. et al. 2001. Evaluation of a portable low cost videophone system in the assessment of depressive symptoms and cognitive function in elderly medically ill veterans. *Journal of Nervous and Mental Disease*. **189:** 399-401.  Mettner J. 2013. The doctor is in another town: telepsychiatry brings care to people in rural Minnesota. *Minnesota Medicine*. **96**: 22-5.  Mielonen, M., Ohinmaa, A., Moring, J., Isohanni, M. 2002. Videoconferencing in telepsychiatry. *Journal of Technology in Human Services.* **20:** 183-199.  Clarke CS. 2018. Telepsychiatry in Asperger's syndrome. *Irish Journal of Psychological Medicine.* **35:** 325-328.  Ikelheimer, D.M. 2008. Treatment of opioid dependence via home- based telepsychiatry. *Psychiatric Services.* **59:** 1218-1219.  Benyakorn S. 2016. Implementing Telepsychiatry in Thailand Benefits and Challenges. *Journal of the Medical Association of Thailand*. **99**: S260-S266.  Hilty DM., Yellowlees, P.M. 2015. Collaborative mental health services using multiple technologies: The new way to practice and a new standard of practice? *Journal of the American Academy of Child & Adolescent Psychiatry.* **54:** 245-246.  Gabel S. 2009. Telepsychiatry, public mental health, and the workforce shortage in child and adolescent psychiatry. *Journal of the American Academy of Child & Adolescent Psychiatry.* **48:** 1127-1128.  Heravian A, Chang BP. 2018. Mental health and telemedicine in the acute care setting: Applications of telepsychiatry in the ED.*The American Journal of Emergency Medicine.* **36:** 1118-1119  Greenwood, J., Chamberlain, C., Parker, G. 2004. Evaluation of a rural telepsychiatry service. *Australasian Psychiatry.* **12:** 268-272.  Whitten P, Kuwahara E. 2004. A multi-phase telepsychiatry programme in Michigan: organizational factors affecting utilization and user perceptions. *Journal of Telemedicine and Telecare.* **10:** 254-261.  Mucic D. 2008. International telepsychiatry: a study of patient acceptability. *Journal of Telemedicine and Telecare.* **14:** 241-243.  Myers KM, Valentine JM, Melzer SM. 2008. Child and adolescent telepsychiatry: utilization and satisfaction.*Telemedicine and e-Health.* **14:** 131-137.  Nelson EL, Bui TN, Velasquez SE. 2011. Telepsychology: research and practice overview. *Child and Adolescent Psychiatric Clinics of North America.* **20:** 67-79.  Ye J, Shim R, Lukaszewski T, Yun K, Kim SH, Ruth G. 2012. Telepsychiatry services for Korean immigrants. *Telemedicine and e-Health*. 2012; **18:** 797-802.  Narasimhan M, Druss BG, Hockenberry JM, Royer J, Weiss P, Glick G, Marcus SC, Magill J. 2015. Impact of a Telepsychiatry Program at Emergency Departments Statewide on the Quality, Utilization, and Costs of Mental Health Services. *Psychiatric Services.* **66:** 1167-72  May C, Gask L, Ellis N, Atkinson T, Mair F, Smith C, Pidd S, Esmail A. 2000. Telepsychiatry evaluation in the north-west of England: preliminary results of a qualitative study. *Journal of Telemedicine and Telecare.* **6:** S20-22.  Matsuura S, Hosaka T, Yukiyama T, Ogushi Y, Okada Y, Haruki Y, Nakamura M. 2000. Application of telepsychiatry: a preliminary study. *Psychiatry and Clinical Neurosciences.* **54:** 55-58.  Rohland BM, Saleh SS, Rohrer JE, Romitti PA. 2000. Acceptability of telepsychiatry to a rural population. *Psychiatric Services.* **51:** 672-674.  Brodey BB, Claypoole KH, Motto J, Arias RG, Goss R. 2000. Satisfaction of forensic psychiatric patients with remote telepsychiatric evaluation. *Psychiatric Services.* **51:** 1305-1307.  May C, Gask L, Atkinson T, Ellis N, Mair F, Esmail A. 2001. Resisting and promoting new technologies in clinical practice: the case of telepsychiatry. *Social Science & Medicine.* **52:** 1889-1901.  Johnston D, Jones BN 3rd. Telepsychiatry consultations to a rural nursing facility: a 2-year experience. *Journal of Geriatric Psychiatry and Neurology.* **14:** 72-75.  Rohland BM. 2001. Telepsychiatry in the heartland: if we build it, will they come?. *Community Mental Health Journal.* **37:** 449-459.  Pollard SE, LePage JP. 2001. Telepsychiatry in a rural inpatient setting. *Psychiatric Services.* **52:** 1659.  Krupinski EA, Barker G, Lopez AM, Weinstein RS. 2004. An analysis of unsuccessful teleconsultations. *Journal of Telemedicine Telecare.* **10:** 6-10.  Myers K, Valentine J, Morganthaler R, Melzer S. 2006. Telepsychiatry with incarcerated youth. *Journal of Adolescent Health.* **38:** 643-648.  Fox KC, Connor P, McCullers E, Waters T. 2008. Effect of a behavioural health and specialty care telemedicine programme on goal attainment for youths in juvenile detention. *Journal of Telemedicine and Telecare.* **14:** 227-230.  Saleem Y, Taylor MH, Khalifa N. 2008. Forensic telepsychiatry in the United Kingdom. *Behavioural Sciences and the Law*. **26:** 333-344.  Rabinowitz T, Murphy KM, Amour JL, Ricci MA, Caputo MP, Newhouse PA. 2010. Benefits of a telepsychiatry consultation service for rural nursing home residents. *Telemedicine and e-Health.* **16:** 34-40.  Ulzen T, Williamson L, Foster PP, Parris-Barnes K. 2013. The evolution of a community-based telepsychiatry program in rural Alabama: lessons learned-a brief report. *Community Mental Health Journal.* **49:** 101-5.  Myers K. 2013. Telepsychiatry: time to connect. *Journal of the American Academy of Child and Adolescent Psychiatry.* **52:** 217-219.  Wallace D, Rayner S. 2013. Telepsychiatry services in the Australian Defence Force. *Australasian Psychiatry.* **21:** 278-279.  Jefee-Bahloul H. 2014. Use of telepsychiatry in areas of conflict: the Syrian refugee crisis as an example. *Journal of Telemedicine and Telecare.* **20:** 167-168.  Nassan M, Frye MA, Adi A, Alarcón RD. 2015. Telepsychiatry for post-traumatic stress disorder: a call for action in the Syrian conflict. *Lancet Psychiatry*. **2:** 866.  Das S, Manjunatha N, Kumar CN, Math SB, Thirthalli J. 2020. Tele-psychiatric after care clinic for the continuity of care: A pilot study from an academic hospital. *Asian Journal of Psychiatry.* **48:** 101886.  Frueh BC, Deitsch SE, Santos AB, Gold PB, Johnson MR, Meisler N, Magruder KM, Ballenger JC. 2000. Procedural and methodological issues in telepsychiatry research and program development. *Psychiatric Services.* **51:** 1522-1527.  Monnier J, Knapp RG, Frueh BC. 2003. Recent advances in telepsychiatry: an updated review. *Psychiatric Services.* **54:** 1604-1609.  McGinty KL, Saeed SA, Simmons SC, Yildirim Y. 2006. Telepsychiatry and e-mental health services: potential for improving access to mental health care. *Psychiatric Quarterly.* **77:** 335-342.  Urness D, Wass M, Gordon A, Tian E, Bulger T. 2006. Client acceptability and quality of life--telepsychiatry compared to in-person consultation. *Journal of Telemedicine and Telecare.* **12:** 251-254.  Modai I, Jabarin M, Kurs R, Barak P, Hanan I, Kitain L. 2007. Cost effectiveness, safety, and satisfaction with video telepsychiatry versus face-to-face care in ambulatory settings. *Telemedicine and e-Health*. **12:** 515-520.  Myers KM, Valentine JM, Melzer SM. 2007. Feasibility, acceptability, and sustainability of telepsychiatry for children and adolescents. *Psychiatric Services.* **58:** 1493-1496.  Yellowlees P, Burke MM, Marks SL, Hilty DM, Shore JH. 2008. Emergency telepsychiatry. *Journal of Telemedicine and Telecare.* **14:** 277-281.  García-Lizana F, Muñoz-Mayorga I. 2010. What about telepsychiatry? A systematic review.*The Primary Care Companion to the Journal of Clinical Psychiatry.* **12.**  Myers KM, Vander Stoep A, McCarty CA, Klein JB, Palmer NB, Geyer JR, Melzer SM. 2010. Child and adolescent telepsychiatry: variations in utilization, referral patterns and practice trends. *Journal of Telemedicine and Telecare.* **16:** 128-133.  Szeftel R, Mandelbaum S, Sulman-Smith H, Naqvi S, Lawrence L, Szeftel Z, Coleman S, Gross L. 2011. Telepsychiatry for children with developmental disabilities: applications for patient care and medical education. *Child and Adolescent Psychiatric Clinics in North America.* **20:** 95-111.  Pignatiello A, Teshima J, Boydell KM, Minden D, Volpe T, Braunberger PG. 2011. Child and youth telepsychiatry in rural and remote primary care. *Child and Adolescent Psychiatric Clinics in North America.* **20:** 13-28.  Grady BJ, Lever N, Cunningham D, Stephan S. 2011. Telepsychiatry and school mental health. *Child and Adolescent Psychiatric Clinics in North America.* **20:** 81-94.  Wood J, Stathis S, Smith A, Krause J. 2012. E-CYMHS: an expansion of a child and youth telepsychiatry model in Queensland. *Australasian Psychiatry.* **20:P** 333-337.  Ramos-Ríos R, Mateos R, Lojo D, Conn DK, Patterson T. 2012. Telepsychogeriatrics: a new horizon in the care of mental health problems in the elderly. *International Psychogeriatrics.* **24:** 1708-1724.  Shore JH. 2013. Telepsychiatry: videoconferencing in the delivery of psychiatric care. *American Journal of Psychiatry.* **170:** 256-262.  Ellington E. 2013. Telepsychiatry by APRNs: an answer to the shortage of pediatric providers?. *Issues in Mental Health Nursing.* **34:** 719-721.  Salmoiraghi A, Hussain S. 2015. A Systematic Review of the Use of Telepsychiatry in Acute Settings. *Journal of Psychiatric Practice*. **21:** 389-393.  Shore J. 2015. The evolution and history of telepsychiatry and its impact on psychiatric care: Current implications for psychiatrists and psychiatric organizations. *International Review of Psychiatry.* **27:** 469-475.  Yellowlees P, Richard Chan S, Burke Parish M. 2015. The hybrid doctor-patient relationship in the age of technology - Telepsychiatry consultations and the use of virtual space. *International Review Psychiatry.* **27:** 476-489.  Fortney JC, Pyne JM, Turner EE, Farris KM, Normoyle TM, Avery MD, Hilty DM, Unützer J. 2015. Telepsychiatry integration of mental health services into rural primary care settings. *International Review Psychiatry.* **27:** 525-539.  Lauckner C, Whitten P. 2016. The State and Sustainability of Telepsychiatry Programs. *The Journal of Behavioural Health Services and Research.* **43:** 305-318.  Nelson EL, Cain S, Sharp S. 2017. Considerations for Conducting Telemental Health with Children and Adolescents. *Child and Adolescent Psychiatric Clinics in North America.* **26:** 77-91.  Roberts N, Hu T, Axas N, Repetti L. 2017. Child and Adolescent Emergency and Urgent Mental Health Delivery Through Telepsychiatry: 12-Month Prospective Study. *Telemedicine and e-Health.* **23:** 842-846.  Serhal E, Crawford A, Cheng J, Kurdyak P. 2017. Implementation and Utilisation of Telepsychiatry in Ontario: A Population-Based Study. *Canadian Journal of Psychiatry.* **62:** 716-725.  Swanson CL, Trestman RL. 2018. Rural Assertive Community Treatment and Telepsychiatry. *Journal of Psychiatric Practice.* **24:** 269-273.  Hassan A, Sharif K. 2019. Efficacy of Telepsychiatry in Refugee Populations: A Systematic Review of the Evidence. *Cureus.* **11**: e3984.  Roth DE, Ramtekkar U, Zeković-Roth S. 2019. Telepsychiatry: A New Treatment Venue for Pediatric Depression. *Child and Adolescent Psychiatric Clinics in North America.* **28:** 377-395.  Smith K, Ostinelli E, Macdonald O, Cipriani A. 2020. COVID-19 and telepsychiatry: an evidence-based guidance for clinicians. *Journal of Medical Internet Research - International Scientific Journal for Medical Research.* **10.**  Myers K., Vander Stoep A., McCarty C.A., Katon W. 2015. Effectiveness of a telehealth service model for treating attention-deficit/hyperactivity disorder: a community-based randomized controlled trial. Journal of the American Academy of Child and Adolescent Psychiatry. 54: 263-274.  Shulman M., John M., Kane J.M. 2017. Home-based outpatient telepsychiatry to improve adherence with treatment appointments: a pilot study. *Psychiatric services.* **68:** 743-746.  O’Reilly R., Bishop J., Maddox K., Hutchinson L., Fisman M., Takhar J. 2007. Is telepsychiatry equivalent to face-to-face psychiatry? Results from a randomized controlled equivalence trial. *Psychiatric Services*. **58:** 863-843  Fishkind, Avrim B & Cuyler, Robert N. 2013. The role of telepsychiatry. Zun, Leslie S [Ed], Chepenik, Lara G [Ed], Mallory, Mary Nan S [Ed]. Behavioral emergencies for the emergency physician. New York, NY, US: Cambridge University Press, US; pp. 303-307.  Simpson J, Doze S, Urness D, Hailey D, Jacobs P. 2001. Evaluation of a routine telepsychiatry service. *Journal of Telemedicine and Telecare.* **7:** 90-98.  Mahmoud H, Vogt E. 2019. Telepsychiatry: an Innovative Approach to Addressing the Opioid Crisis. *Journal of Behavioural Health Sciences and Research.* **46:** 680-685.  Jones BN 3rd. 2001. Telepsychiatry and geriatric care. *Current Psychiatry Reports.* **3:** 29-36.  Zaylor C, Whitten P, Kingsley C. 2000. Telemedicine services to a county jail. *Journal of Telemedicine and Telecare.* **6:** S93-5.  Zaylor C, Nelson EL, Cook DJ. 2001. Clinical outcomes in a prison telepsychiatry clinic. *Journal of Telemedicine and Telecare.* **1:** 47-49.  Yellowlees P, Nakagawa K, Pakyurek M, Hanson A, Elder J, Kales HC. 2020. Rapid  Conversion of an Outpatient Psychiatric Clinic to a 100% Virtual Telepsychiatry  Clinic in Response to COVID-19. *Psychiatric Services.* **71:** 749-752.  Work Group on Quality Issues (WGQI) & American Academy of Child and Adolescent Psychiatry (AACAP), US. 2008. Practice parameter for telepsychiatry with children and adolescents. *Journal of the American Academy of Child & Adolescent Psychiatry.* **47:** 1468-1483.  Wilshire, Thea W. 2012. Telepsychiatry services at a tribally run behavioral health clinic. *Psychological Services*. **9:** 318-319.  Whaibeh, Emile, Mahmoud, Hossam & Vogt, Emily L. 2019. Reducing the treatment gap for lgbt mental health needs: The potential of telepsychiatry. *The Journal of Behavioral Health Services & Research*. No Pagination Specified  Wallace, Duncan & Hodges, Samantha. 2018. Telepsychiatry in the Australian Defence Force: A success story. *Australasian Psychiatry*. **26:** 105-106.  Vought, Rhonda G, Grigsby, R. Kevin, Adams, Laura N & Shevitz, Stewart A. 2000. Telepsychiatry: Addressing mental health needs in Georgia. *Community Mental Health Journal.* **36:** 525-536.  Volpe T, Boydell KM, Pignatiello A. 2013. Attracting Child Psychiatrists to a Televideo Consultation Service: The TeleLink  Experience.*International Journal of Telemedicine and Applications.* **2013:** 146858.  Volicer L. 2015. Nursing home telepsychiatry. *Journal of the American Medical Directors Association*. **16**: 7-8.  Vernig, P.M. 2016. Telemental health: Digital disruption and the opportunity to expand care. *Journal of the American Psychiatric Nurses Association*. **22:** 73-75.  van Wynsberghe A, Gastmans C. 2009. Telepsychiatry and the meaning of in-person contact: a preliminary ethical appraisal. *Medicine, Health Care and Philosophy.* **12:** 469-476.  Valdagno M, Goracci A, di Volo S, Fagiolini A. 2014. Telepsychiatry: new perspectives and open issues. *CNS Spectrums.* **19:** 479-481.  Turner, J.W. 2001. Telepsychiatry as a case study of presence: Do you know what you are missing? *Journal of Computer-Mediated Communication.* **6.**  Trondsen MV, Bolle SR, Stensland GØ, Tjora A. 2014. Video-confidence:  a qualitative exploration of videoconferencing for psychiatric  emergencies. *BMC Health Service Research*. **14:** 544.  Toperczer T. 2011. Telepsychiatry in the cloud: reaching rural communities  in underserved markets. *Health Management Technology.* **32**: 28-9.  Toombs E, Kowatch KR, Dalicandro L, McConkey S, Hopkins C, Mushquash CJ. 2020. A systematic  review of electronic mental health interventions for Indigenous youth: Results  and recommendations. *Journal of Telemedicine and Telecare.* **14:** 1357633X19899231.  Thompson D.A., Leimig R., Gower G., Winsett R.P. 2009. Assessment of depressive symptoms during post-transplant follow-up care performed via telehealth. *Telemedicine journal and e-health*. **15:** 700-706.  Thomas JF, Novins DK, Hosokawa PW, Olson CA, Hunter D, Brent AS, Frunzi G, Libby AM. 2018. The Use of  Telepsychiatry to Provide Cost-Efficient Care During Pediatric Mental Health Emergencies. *Psychiatric Services.* **1:** 161-168.  Taylor M, Kikkawa N, Hoehn E, Haydon H, Neuhaus M, Smith AC, Caffery LJ. 2019. The importance of external clinical facilitation for a perinatal and infant telemental health service. *Journal of Telemedicine and Telecare.* **25:** 566-571.  Tang S, Helmeste D. 2000. Digital psychiatry. *Psychiatry and Clinical Neurosciences.* **54:** 1-10.  Swanson B. 1999. Information technology and under-served  communities. *Journal of Telemedicine and Telecare.* **5:** S3-10.  Sulzbacher S, Vallin, T, Waetzig E.Z. 2006. Telepsychiatry improves paediatric behavioural health care in rural communities. *Journal of Telemedicine and Telecare.* **12:** 285-288  Stevens, A., Doidge, N., Goldbloom, D., Voore, P., Farewell, J. 1999. Pilot study of televideo psychiatric assessments in an underserviced community. *The American Journal of Psychiatry.* **156:** 783-785  Starling, J., Foley, S. 2006. From pilot to permanent service: Ten years of paediatric telepsychiatry. *Journal of Telemedicine and Telecare.* **12:** 80-82.  Starling, J., Dossetor, D. 2005. Child and Adolescent Telepsychiatry. Wootton, Richard [Ed], Batch, Jennifer [Ed]. Telepediatrics: Telemedicine and child health. Lo: Royal Society of Medicine Press; pp. 77-87.  Spaulding, R., Cain, S., Sonnenschein, K. 2011. Urban telepsychiatry: Uncommon service for a common need. *Child and Adolescent Psychiatric Clinics of North America.* **20:** 29-39  Sousa A, Karia S. 2020. Telepsychiatry  during COVID-19: Some clinical, public health, and ethical dilemmas. *Indian Journal of Public Health.* **64:** S245-S246.  Mucic, D. 200). Telepsychiatry within European e-health. Lazakidou, Athina A [Ed], Siassiakos, Konstantinos M [Ed]. Handbook of research on distributed medical informatics and e-health. Hershey, PA, US: Medical Information Science Reference/IGI Global, US; pp. 129-136.  Pakyurek, M, Yellowlees, P., Hilty, D. 2010. The child and adolescent telepsychiatry consultation: Can it be a more effective clinical process for certain patients than conventional practice? *Telemedicine and e-Health.* **16:** 289-292.  Pesämaa L, Ebeling H, Kuusimäki ML, Winblad I, Isohanni M, Moilanen I. 2004. Videoconferencing in child and adolescent telepsychiatry: a systematic review of the literature. *Journal of Telemedicine and Telecare.* **10:** 187-192.  Reinhardt I, Gouzoulis-Mayfrank E, Zielasek J. 2019. Use of Telepsychiatry in Emergency and Crisis Intervention: Current Evidence. *Current Psychiatric Reports.* **1:** 63.  Reliford A, Adebanjo B. 2019. Use of Telepsychiatry in Pediatric Emergency Room to Decrease Length of Stay for Psychiatric Patients, Improve Resident On-Call Burden, and Reduce Factors Related to Physician Burnout. *Telemedicine and e-Health.* **25**: 828-832.  Report from the Alberta Heritage Foundation for Medical Research. 1998. Evaluation of a telepsychiatry pilot project. *International Journal of Technology Assessment in Health Care. 1998;* **14:** 583-4.  Rowe N, Gibson S, Morley S, Krupinski EA. 2008. Ten-year experience of a private nonprofit telepsychiatry service. *Telemedicine and e-Health.* **14:** 1078-1086.  McLaren P, Ahlbom J, Riley A, Mohammedali A, Denis M. 2002. The North Lewisham telepsychiatry project: beyond the pilot phase. *Journal of Telemedicine and Telecare.* **8:** 90-100.  Sales, C.P., McSweeney, L., Saleem, Y., Khalifa, N. 2018. The use of telepsychiatry within forensic practice: A literature review on the use of videolink-A ten-year follow-up. *Journal of Forensic Psychiatry & Psychology.* **29:** 387-402.  Samuels, A. 1999. International telepsychiatry: A link between New Zealand and Australia. *Australian and New Zealand Journal of Psychiatry*. **33:** 284-286.  Savin, D., Garry, M.T., Zuccaro, P., Novins, D. 2006. Telepsychiatry for treating rural American Indian Youth. *Journal of the American Academy of Child & Adolescent Psychiatry*. **45:** 484-488.  Schubert NJ, Backman PJ, Bhatla R, Corace KM. 2019. Telepsychiatry and patient-provider concordance. *Canadian Journal of Rural Medicine.* **24:** 75-82.  Seritan AL, Heiry M, Iosif AM, Dodge M, Ostrem JL. 2019. Telepsychiatry  for patients with movement disorders: a feasibility and patient satisfaction  study. *Journal of Clinical Movement Disorders.* **6:** 1.  Shores, M.M., Ryan-Dykes, P., Williams, R.M., Mamerto, B., Sadak, T., Pascualy, M. et al. 2004. Identifying undiagnosed dementia in residential care veterans: comparing telemedicine to in-person clinical examination. *International Journal of Geriatric Psychiatry.* **19:** 101-108  Simpson J, Doze S, Urness D, Hailey D, Jacobs P. 2001. Telepsychiatry  as a routine service--the perspective of the patient. *Journal of Telemedicine and Telecare.* **7:** 155-160. |
| Technology | Aboujaoude, E. 2018. Telemental health: Why the revolution has not arrived. *World Psychiatry.* **17:** 277-278  Abrams J, Sossong S, Schwamm LH, Barsanti L, Carter M, Kling N, Kotarski M, Leddy J, Meller B, Simoni M, Sullivan M, Wozniak J. Practical Issues in Delivery of Clinician-to-Patient Telemental Health in an Academic Medical Center. *Harvard Review of Psychiatry.* 2017; **25**:135-145.  Agarwal PP, Manjunatha N, Gowda GS, Kumar MNG, Shanthaveeranna N, Kumar CN, Math SB. 2019. Collaborative Tele-Neuropsychiatry Consultation Services for Patients in Central Prisons. *Journal of Neurosciences in Rural Practice.* **10**:101-105.  American Academy of Child and Adolescent Psychiatry (AACAP) Committee on Telepsychiatry and AACAP Committee on Quality Issues. 2017. Clinical update: Telepsychiatry with children and adolescents. *Journal of the American Academy of Child & Adolescent Psychiatry.* **56**: 875-893  Augusterfer EF, Mollica RF, Lavelle J. 2018. Leveraging - Technology in Post-Disaster Settings: the Role of Digital Health/Telemental Health. *Current Psychiatry Reports*. **20:** 88.  Barnett, Michael L & Huskamp, Haiden A. 2020. Telemedicine for mental health in the United States: Making progress, still a long way to go. *Psychiatric Services.* **71:** 197-198.  Batastini, Ashley B, McDonald, Brendan R & Morgan, Robert D. 2013. Videoteleconferencing in forensic and correctional practice. Myers, Kathleen [Ed], Turvey, Carolyn L [Ed]. Telemental health: Clinical, technical, and administrative foundations for evidence-based practice. Amsterdam, Netherlands: Elsevier, Netherlands; pp. 251-271.  Boydell KM, Hodgins M, Pignatiello A, Teshima J, Edwards H, Willis D. 2014. Using technology to deliver mental health services to children and youth: a scoping review. *Journal of the Canadian Academy of Child and Adolescent Psychiatry*. **23**:87-99.  Boydell, K.M., Volpe, T., Kertes, A., Greenberg, N. 2007. A review of the outcomes of the recommendations made during paediatric telepsychiatry consultations. *Journal of Telemedicine and Telecare.* **13:** 277-281.  Brown FW. 1998. Rural telepsychiatry. *Psychiatric Services.* **49**:963-4.  Buist A, Coman G, Silvas A, Burrows G. 2000. An evaluation of the telepsychiatry programme in Victoria, Australia. *Journal of Telemedicine and Telecare.* **6**: 216-21.  Campbell R, O'Gorman J, Cernovsky ZZ. 2015. Reactions of Psychiatric Patients to Telepsychiatry. *Mental Illness Journal.* **7**: 6101.  Chakrabarti S. 2015. Usefulness of telepsychiatry: A critical evaluation of videoconferencing-based  approaches. *World Journal of Psychiatry.* **22:** 286-304.  Chan S, Parish M, Yellowlees P. 2015. Telepsychiatry Today.*Current Psychiatry Reports.* **17:** :89  Caudill, R.L., Sager, Z. 2015. Institutionally based videoconferencing. *International Review of Psychiatry*. **27:** 496-503.  Chipps J, Ramlall S, Madigoe T, King H, Mars M. 2012. Developing telepsychiatry services in KwaZulu-Natal -- an action research study. *African Journal of Psychiatry*. **15:** 255-63.  Chipps J, Ramlall S, Mars M. 2012. A telepsychiatry model to support psychiatric outreach in the public sector in South Africa. *African Journal of Psychiatry.* **15**:264-70.  Chipps J, Ramlall S, Mars M. 2012. Practice guidelines for videoconference-based telepsychiatry in South Africa. *African Journal of Psychiatry.* **15:** :271-82.  Chong, J., Moreno, F. 2012. Feasibility and acceptability of clinic-based telepsychiatry for low-income Hispanic primary care patients. *Telemedicine and e-Health.* **18**: 297-304  American Academy of Child and Adolescent Psychiatry (AACAP) Committee on Telepsychiatry and AACAP Committee on Quality Issues. 2017. Clinical Update: Telepsychiatry With Children and Adolescents. *Journal of the American Academy of Child & Adolescent Psychiatry.* **56:** 875-893  Cowan KE, McKean AJ, Gentry MT, Hilty DM. 2019. Barriers to Use of Telepsychiatry: Clinicians as Gatekeepers. *Mayo Clinic Proceedings.* **94:** 2510-2523.  Deslich S, Stec B, Tomblin S, Coustasse A. 2013. Telepsychiatry  in the 21(st) century: transforming healthcare with technology. *Perspectives in Health Information Management.* **10:** 1f.  Deslich SA, Thistlethwaite T, Coustasse A. 2013. Telepsychiatry  in correctional facilities: using technology to improve access and decrease  costs of mental health care in underserved populations. *The Permanente Journal* **17:** 80-86.  Detweiler MB, Arif S, Candelario J, Altman J, Murphy PF, Halling MH, Detweiler JG, Vasudeva S. 2011. A telepsychiatry transition clinic: the first 12 months  experience. *Journal of Telemedicine and Telecare.* **17:** 293-297.  Dham, P., Gupta, N., Alexander, J., Black, W., Rajji, T., Skinner, E. 2018. Community based telepsychiatry service for older adults residing in a rural and remote region- utilization pattern and satisfaction among stakeholders. *BMC Psychiatry.* 18.  Donley, E., McClaren, A., Jones, R., Katz, P., Goh, J. 2017. Evaluation and implementation of a telepsychiatry trial in the emergency department of a metropolitan public hospital. *Journal of Technology in Human Services*. **35:** 292-313.  Fatehi, F., Armfield, N.R., Dimitrijevic, M., Gray, L.C. 2014. Clinical applications of videoconferencing: A scoping review of the literature for the period 2002-2012. J*ournal of Telemedicine and Telecare*. **20:** 377-383.  Fegert JM, Vitiello B, Plener PL, Clemens V. 2020. Challenges and  burden of the Coronavirus 2019 (COVID-19) pandemic for child and adolescent  mental health: a narrative review to highlight clinical and research needs in  the acute phase and the long return to normality. *Child and Adolescent Psychiatry and Mental Health.* **14:** 20.  Fortney JC, Heagerty PJ, Bauer AM, Cerimele JM, Kaysen D, Pfeiffer PN, Zielinski MJ, Pyne JM, Bowen D, Russo J, Ferro L, Moore D, Nolan JP, Fee FC, Heral T, Freyholtz-London J, McDonald B, Mullins J, Hafer E, Solberg L, Unützer J. 2020. Study to promote innovation in rural integrated telepsychiatry (SPIRIT): Rationale and design of a randomized comparative effectiveness trial of managing complex psychiatric disorders in rural primary care clinics. *Contemporary Clinical Trials.* **90:**  105873.  Gammon D, Bergvik S, Bergmo T, Pedersen S.1996. Videoconferencing  in psychiatry: a survey of use in northern Norway. *Journal of Telemedicine and Telecare.* **2:** 192-198.  Gardner JS, Plaven BE, Yellowlees P, Shore JH. 2020. Remote Telepsychiatry Workforce: a Solution to Psychiatry's Workforce Issues. *Current Psychiatric Reports.* **22:** 8.  Elford DR, White H, St John K, Maddigan B, Ghandi M, Bowering R. 2001. A prospective satisfaction study and cost analysis of a pilot child telepsychiatry service in Newfoundland. *Journal of Telemedicine and Telecare.* **7:** 73-81.  Gabel S. 2009. Telepsychiatry, public mental health, and the workforce shortage in child and adolescent psychiatry. *Journal of the American Academy of Child & Adolescent Psychiatry.* **48:** 1127-1128.  Gelber H, Alexander M. 1999. An evaluation of an Australian videoconferencing project for child and adolescent telepsychiatry. *Journal of Telemedicine and Telecare.* **5**: S21-3.  Gelber H. 2001. The experience in Victoria with telepsychiatry for the child and adolescent mental health service. *Journal of Telemedicine and Telecare.* **7:** 32-4.  Elford R., White H., Bowering R., Ghandi A., Maddiggan B., St John K., House M., Harnett J., West R., Battcock A. 2000. A randomized, controlled trial of child psychiatric assessments conducted using videoconferencing. *Journal of telemedicine and telecare.* **6:** 73-82  Gloff, N.E, LeNoue, S.R, Novins, D.K., Myers, K. 2015. Telemental health for children and adolescents. *International Review of Psychiatry*. **27:** 513-524.  Glueck DA. 2011. Telepsychiatry in private practice. *Child and Adolescent Psychiatric Clinics of North America*. **20**: 1-11.  Gowda, Guru S, Kulkarni, Karishma, Bagewadi, Virupaksha, R. P. S., Shyam, Manjunatha, B. R, Shashidhara, Harihara N, et al. 2018. A study on collaborative telepsychiatric consultations to outpatients of district hospitals of Karnataka, India. *Asian Journal of Psychiatry.* **37:** 161-166  Grady B. 2012. Promises and limitations of telepsychiatry in rural adult mental health care. *World Psychiatry.* **11**: 199-201.  Grubaugh, Anouk L, Cain, Gregory D, Elhai, Jon D, Patrick, Sarah L & Frueh, B. Christopher. 2008. Attitudes toward medical and mental health care delivered via telehealth applications among rural and urban primary care patients. *Journal of Nervous and Mental Disease.* **19:** 166-170.  Haghnia Y., Samad-Soltani T., Yousefi M., Sadr H., Rezaei-Hachesu P. 2010. Telepsychiatry-based care for the treatment follow-up of Iranian war veterans with post-traumatic stress disorder: a randomized controlled trial. *Iranian journal of medical sciences*. **44**: 291-298.  Graham, M.A. 1996. Telepsychiatry in Appalachia. *American Behavioral Scientist*. **39:** 602-615.  Hailey D, Jacobs P, Simpson J, Doze S. 1999. An assessment framework for telemedicine applications. *Journal of telemedicine and telecare.* **5**:162-70.  Hailey, David, Ohinmaa, Arto & Roine, Risto. 2009. Limitations in the routine use of telepsychiatry. *Journal of Telemedicine and Telecare.* **15:** 28-31.  Hampton, T. 2006. Researchers provide psychiatric care from afar. *The Journal of the American Medical Association*: **295:** 21-22.  Haslam R, McLaren P. Interactive  television for an urban adult mental health service: the Guy's Psychiatric  Intensive Care Unit Telepsychiatry Project. *Journal of Telemedicine and Telecare. 2000.* **6:** S50-2  Hilt, R.J. Telemedicine for child collaborative or integrated care. *Child and Adolescent Psychiatric Clinics of North America. 2017.* **26:** 637-645.  Hilty DM, Ferrer DC, Parish MB, Johnston B, Callahan EJ, Yellowlees PM. 2013. The effectiveness of telemental health. *Journal of telemedicine and telecare.* **19:** 444-454.  Hilty DM, Luo, John S, Morache, Chris, Marcelo, Divine A & Nesbitt, Thomas S. 2002. Telepsychiatry: An overview for psychiatrists. *CNS Drugs.* **16:** 527-548.  Hilty DM, Marks, S.L., Urness, D., Yellowlees, P.M., Nesbitt, T.S.2004. Clinical and Educational Telepsychiatry Applications: A Review. *The Canadian Journal of Psychiatry / La Revue canadienne de psychiatrie*. **49:** 12-23.  Hungerbuehler I, Valiengo L, Loch AA, Rössler W, Gattaz WF. 2016. Home-Based  Psychiatric Outpatient Care Through Videoconferencing for Depression: A  Randomized Controlled Follow-Up Trial. *Journal of Medical Internet Research.* **3:** e36.  Jacob MK, Larson JC, Craighead WE. 2012. Establishing a telepsychiatry consultation practice in rural Georgia for primary care  physicians: a feasibility report. *Clinical Paediatrics.* **51:** 1041-1047.  Jefee-Bahloul H. 2014. Telemental health in the middle East: overcoming the barriers. *Frontiers in Public Health*. **2:** 86  Jones, Roland M, Leonard, S., Birmingham, L. 2006. Setting up a telepsychiatry service. *Psychiatric Bulletin*. **30:** 464-467  Kaftarian E. Lessons Learned in Prison and Jail-Based Telepsychiatry. *Current Psychiatric Reports*. **21:** 15.  Kannarkat JT, Smith NN, McLeod-Bryant SA. Mobilization of Telepsychiatry in Response to COVID-19-Moving Toward 21st Century  Access to Care. *Administration and Policy in Mental Health.* **47**:489-491.  Katz CL, Washington FB, Sacco M, Schuetz-Mueller J. A Resident-Based Telepsychiatry Supervision Pilot Program in Liberia. *Psychiatric Services.* **70:** 243-246.  Kennedy C, Yellowlees P. 2000. A community-based approach to evaluation of health outcomes and costs for telepsychiatry in a rural population: preliminary results. *Journal of Telemedicine and Telecare*. **6:** S155-7.  Khalifa, N, Saleem, Y., Stankard, P. 2008. The use of telepsychiatry within forensic practice: A literature review on the use of videolink. *Journal of Forensic Psychiatry & Psychology.* **19:** 2-13.  Kopel H, Nunn K, Dossetor D. 2001. Evaluating satisfaction with a child and adolescent psychological telemedicine outreach  service. *Journal of Telemedicine and Telecare*. **7**: 35-40.  LaBelle B, Franklyn AM, Pkh Nguyen V, Anderson KE, Eibl JK, Marsh DC. 2018. Characterizing the Use of Telepsychiatry for Patients with Opioid Use Disorder and Cooccurring Mental Health Disorders in Ontario, Canada. *International Journal of Telemedicine and Applications.* **2018:** 7937610.  Lal, S., Abdel-Baki, A., Sujanani, S., Bourbeau, F., Sahed, I., Whitehead, J. 2020. Perspectives of young adults on receiving telepsychiatry services in an urban early intervention program for first-episode psychosis: A cross-sectional, descriptive survey study. *Frontiers in Psychiatry*. 11  Langarizadeh M, Tabatabaei MS, Tavakol K, Naghipour M, Rostami A, Moghbeli F. 2017. Telemental Health Care, an Effective Alternative to Conventional Mental Care: a Systematic  Review. *Acta Informatica Medica*. **25:** 240-246.  Lee A, S.N., O'Connell F, Dyer A, Boniface K, Betz J. Telepsychiatric assessment of a mariner expressing suicidal ideation. *International Maritime Health.2 015;* **66**: 49-51.  Leonard S. 2004. The successes and challenges of developing a prison telepsychiatry service. *Journal of Telemedicine and Telecare*. **10**: 69-71.  Lexcen, F.J., Hawk, G.L., Herrick, S., Blank, M.B. 2006. Use of Video Conferencing for Psychiatric and Forensic Evaluations. *Psychiatric Services.* **57:** 713-715.  Looi JC, Pring W. 2020. Private metropolitan telepsychiatry in Australia during  Covid-19: current practice and future developments. *Australas Psychiatry*. **2**: 1039856220930675.  Madhavan G. 2019. Telepsychiatry in intellectual disability psychiatry: literature  review. *BJPsych Bulletin.* **43**: 167-173  Malhotra S, Chakrabarti S, Shah R. 2013. Telepsychiatry:Promise, potential, and challenges. *Indian Journal of Psychiatry.* **55**: 3-11.  Malhotra, S., Shah, R. 2018. Telepsychiatry and digital mental health care in child and adolescent psychiatry: Implications for service delivery in low- and middle-income countries. Hodes, Matthew [Ed], Shur-Fen Gau, Susan [Ed], De Vries, Petrus J [Ed]. Understanding uniqueness and diversity in child and adolescent mental health. San Diego, CA, US: Elsevier Academic Press, US; pp. 263-287  Mannion, L, Fahy, T. J, Duffy, C, Broderick, M., Gethins, E. 1998. 'Telepsychiatry': Keeping a link with an island. *Psychiatric Bulletin.* **22:** 47-49.  Math SB, Moirangthem S, Kumar NC. 2015. Tele-Psychiatry: After Mars, Can we Reach the Unreached?. *Indian Journal of Psychological Medicine.* **37:** 120-1.  May CR, Ellis NT, Atkinson T, Gask L, Mair F, Smith C. 1999. Psychiatry by videophone:  a trial service in north west England. *Studies in Health Technology and Informatics*. **68**: 207-10.  McGrath J. 2020. ADHD and  Covid-19: Current roadblocks and future opportunities. *The Irish Journal of Psychological Medicine.* **21:** 1-22.  McLaren P. 2004. Telepsychiatry in Europe. *International Psychiatry.* **1**: 8-10.  Meltzer, B. 1997. Telemedicine in emergency psychiatry. *Psychiatric Services.* **48:** 1141-1142.  Menon, A. Srikumar, K., Prasad, K., Popuri, Chrismer, J.B., Raskin, A., Hebel, J.R. et al. 2001. Evaluation of a portable low cost videophone system in the assessment of depressive symptoms and cognitive function in elderly medically ill veterans. *Journal of Nervous and Mental Disease*. **189:** 399-401.  Mettner J. 2013. The doctor is in another town: telepsychiatry brings care to people in rural Minnesota. *Minnesota Medicine*. **96**: 22-5.  Mielonen, M., Ohinmaa, A., Moring, J., Isohanni, M. 2002. Videoconferencing in telepsychiatry. *Journal of Technology in Human Services.* **20:** 183-199.  Clarke CS. 2018. Telepsychiatry in Asperger's syndrome. *Irish Journal of Psychological Medicine.* **35:** 325-328.  Ikelheimer, D.M. 2008. Treatment of opioid dependence via home- based telepsychiatry. *Psychiatric Services.* **59:** 1218-1219.  Benyakorn S. 2016. Implementing Telepsychiatry in Thailand Benefits and Challenges. *Journal of the Medical Association of Thailand*. **99**: S260-S266.  Mucic D. 2008. International telepsychiatry: a study of patient acceptability. *Journal of Telemedicine and Telecare.* **14:** 241-243.  Ye J, Shim R, Lukaszewski T, Yun K, Kim SH, Ruth G. 2012. Telepsychiatry services for Korean immigrants. *Telemedicine and e-Health*. **18:** 797-802.  Werner A, Anderson LE. 1998. Rural telepsychiatry is economically unsupportable: the Concorde crashes in a cornfield. *Psychiatric Services.* **49:** 1287-1290.  Matsuura S, Hosaka T, Yukiyama T, Ogushi Y, Okada Y, Haruki Y, Nakamura M. 2000. Application of telepsychiatry: a preliminary study. *Psychiatry and Clinical Neurosciences.* **54:** 55-58.  May C, Gask L, Atkinson T, Ellis N, Mair F, Esmail A. 2001. Resisting and promoting new technologies in clinical practice: the case of telepsychiatry. *Social Science & Medicine.* **52:** 1889-1901.  Johnston D, Jones BN 3rd. Telepsychiatry consultations to a rural nursing facility: a 2-year experience. *Journal of Geriatric Psychiatry and Neurology.* **14:** 72-75.  Jones BN 3rd, Johnston D, Reboussin B, McCall WV. 2001. Reliability of telepsychiatry assessments: subjective versus observational ratings. *Journal of Geriatric Neurology and Psychiatry.* **14:** 66-71.  Miller TW, Clark J, Veltkamp LJ, Burton DC, Swope M. 2008. Teleconferencing model for forensic consultation, court testimony, and continuing education. *Behavioural Sciences and the Law.* **26:** 301-313.  Westphal A, Dingjan P, Attoe R. 2010. What can low and high technologies do for late-life mental disorders?. *Current Opinion in Psychiatry.* **23:** 510-515.  Myers KM, Palmer NB, Geyer JR. Research in child and adolescent telemental health. *Child and Adolescent Psychiatric Clinics in North America. 2011;* **20:** 155-171.  Savin D, Glueck DA, Chardavoyne J, Yager J, Novins DK. 2011. Bridging cultures: child psychiatry via videoconferencing. *Child and Adolescent Psychiatric Clinics in North America.* **20:** 125-134.  Mars M. 2012. Telepsychiatry in Africa -- a way forward?. *African Journal of Psychiatry.* **15:** 215, 217.  Myers K. 2013. Telepsychiatry: time to connect. *Journal of the American Academy of Child and Adolescent Psychiatry.* **52:** 217-219.  Jefee-Bahloul H. 2014. Use of telepsychiatry in areas of conflict: the Syrian refugee crisis as an example. *Journal of Telemedicine and Telecare.* **20:** 167-168.  Sharma A, Sasser T, Schoenfelder Gonzalez E, Vander Stoep A, Myers K. Implementation of Home-Based Telemental Health in a Large Child Psychiatry Department During the COVID-19 Crisis. *Journal of Child and Adolescent Psychopharmacology. 2020; 8.*  Frueh BC, Deitsch SE, Santos AB, Gold PB, Johnson MR, Meisler N, Magruder KM, Ballenger JC. 2000. Procedural and methodological issues in telepsychiatry research and program development. *Psychiatric Services.* **51:** 1522-1527.  Jones BN 3rd, Ruskin PE. 2001. Telemedicine and geriatric psychiatry: directions for future research and policy. *Journal of Geriatric Psychiatry and Neurology.* **14:** 59-62.  Monnier J, Knapp RG, Frueh BC. 2003. Recent advances in telepsychiatry: an updated review. *Psychiatric Services.* **54:** 1604-1609.  Miller TW, Burton DC, Hill K, Luftman G, Veltkemp LJ, Swope M. 2005. Telepsychiatry: critical dimensions for forensic services. *Journal of Academic Psychiatry and Law.* **33:** 539-546.  McGinty KL, Saeed SA, Simmons SC, Yildirim Y. 2006. Telepsychiatry and e-mental health services: potential for improving access to mental health care. *Psychiatric Quarterly.* **77:** 335-342.  Nieves JE, Stack KM. 2007. Hispanics and telepsychiatry. *Psychiatric Services.* **58**: 877-8.  García-Lizana F, Muñoz-Mayorga I. 2010. What about telepsychiatry? A systematic review.*The Primary Care Companion to the Journal of Clinical Psychiatry.* **12.**  Grady BJ, Lever N, Cunningham D, Stephan S. 2011. Telepsychiatry and school mental health. *Child and Adolescent Psychiatric Clinics in North America.* **20:** 81-94.  Ramos-Ríos R, Mateos R, Lojo D, Conn DK, Patterson T. 2012. Telepsychogeriatrics: a new horizon in the care of mental health problems in the elderly. *International Psychogeriatrics.* **24:** 1708-1724.  Shore JH. 2013. Telepsychiatry: videoconferencing in the delivery of psychiatric care. *American Journal of Psychiatry.* **170:** 256-262.  Salmoiraghi A, Hussain S. 2015. A Systematic Review of the Use of Telepsychiatry in Acute Settings. *Journal of Psychiatric Practice*. **21:** 389-393.  Nelson EL, Cain S, Sharp S. 2017. Considerations for Conducting Telemental Health with Children and Adolescents. *Child and Adolescent Psychiatric Clinics in North America.* **26:** 77-91.  Roberts N, Hu T, Axas N, Repetti L. 2017. Child and Adolescent Emergency and Urgent Mental Health Delivery Through Telepsychiatry: 12-Month Prospective Study. *Telemedicine and e-Health.* **23:** 842-846.  Saeed SA. 2018. Tower of Babel Problem in Telehealth: Addressing the Health Information Exchange Needs of the North Carolina Statewide Telepsychiatry Program (NC-STeP). *Psychiatry Quarterly.* **89:** 489-495.  Shulman M., John M., Kane J.M. 2017. Home-based outpatient telepsychiatry to improve adherence with treatment appointments: a pilot study. *Psychiatric services.* **68:** 743-746.  Simpson J, Doze S, Urness D, Hailey D, Jacobs P. 2001. Evaluation of a routine telepsychiatry service. *Journal of Telemedicine and Telecare.* **7:** 90-98.  Mahmoud H, Vogt E. 2019. Telepsychiatry: an Innovative Approach to Addressing the Opioid Crisis. *Journal of Behavioural Health Sciences and Research.* **46:** 680-685.  Zulfic Z, Liu D, Lloyd C, Rowan J, Schubert KO. 2020. Is telepsychiatry care a realistic option for community mental health services during the COVID-19 pandemic?. *Australian and New Zealand Journal of Psychiatry.* **22**;:4867420937788.  Yellowlees, Peter M, Hilty, Donald M & Mucic, Davor. 2016. Global/worldwide e-mental health: International and futuristic perspectives of telepsychiatry and the future. Mucic, Davor [Ed], Hilty, Donald M [Ed]. e-Mental health. Cham, Switzerland: Springer International Publishing, Switzerland; pp. 233-249  Yellowlees P, Nakagawa K, Pakyurek M, Hanson A, Elder J, Kales HC. 2020. Rapid  Conversion of an Outpatient Psychiatric Clinic to a 100% Virtual Telepsychiatry  Clinic in Response to COVID-19. *Psychiatric Services.* **71:** 749-752.  Work Group on Quality Issues (WGQI) & American Academy of Child and Adolescent Psychiatry (AACAP), US. 2008. Practice parameter for telepsychiatry with children and adolescents. *Journal of the American Academy of Child & Adolescent Psychiatry.* **47:** 1468-1483.  Wojtuszek, Magdalena, Kachnic, Justyna, Krysta, Krzysztof & Wutke, Joanna. 2015. Telepsychiatry in Polish patients' and doctors' opinion. *Psychiatria Danubina.* **27:** S379-S382.  Volpe T, Boydell KM, Pignatiello A. 2013. Attracting Child Psychiatrists to a Televideo Consultation Service: The TeleLink  Experience.*International Journal of Telemedicine and Applications.* **2013:** 146858.  Vernig, P.M. 2016. Telemental health: Digital disruption and the opportunity to expand care. *Journal of the American Psychiatric Nurses Association*. **22:** 73-75.  Vega, W.A., Pollitt, A., Mays, R.A. 2007. Reply to Hispanics and Telepsychiatry. *Psychiatric Services*. **58:** 878.  Vanderpool, D. 2015. An overview of practicing high quality telepsychiatry. Dewan, Naakesh A [Ed], Luo, John S [Ed], Lorenzi, Nancy M [Ed]. Mental health practice in a digital world: A clinician's guide. Cham, Switzerland: Springer International Publishing, Switzerland; pp. 159-181  Toombs E, Kowatch KR, Dalicandro L, McConkey S, Hopkins C, Mushquash CJ. 2020. A systematic  review of electronic mental health interventions for Indigenous youth: Results  and recommendations. *Journal of Telemedicine and Telecare.* **14:** 1357633X19899231.  Thomas RK, Suleman R, Mackay M, Hayer L, Singh M, Correll CU, Dursun S. 2020. Adapting to the impact of COVID-19 on mental health: an international perspective. *Journal of Psychiatry and Neuroscience* **45**: 229-233.  Tang S, Helmeste D. 2000. Digital psychiatry. *Psychiatry and Clinical Neurosciences.* **54:** 1-10.  Starling, J., Dossetor, D. 2005. Child and Adolescent Telepsychiatry. Wootton, Richard [Ed], Batch, Jennifer [Ed]. Telepediatrics: Telemedicine and child health. Lo: Royal Society of Medicine Press; pp. 77-87.  Spaulding, R., Cain, S., Sonnenschein, K. 2011. Urban telepsychiatry: Uncommon service for a common need. *Child and Adolescent Psychiatric Clinics of North America.* **20:** 29-39  Sousa A, Karia S. 2020. Telepsychiatry  during COVID-19: Some clinical, public health, and ethical dilemmas. *Indian Journal of Public Health.* **64:** S245-S246.  Moirangthem S, Rao S, Kumar CN, Narayana M, Raviprakash N, Math SB. 2017. Telepsychiatry as an Economically Better Model for Reaching the Unreached: A Retrospective Report from South India. *Indian Journal of Psychological Medicine.* **39:** 271-275.  Munro C.C., Hynan L.S, Grosch M, Parikh M, Weiner MF. Teleneuropsychology:  evidence for video teleconference-based neuropsychological assessment. *Journal of the International Neuropsychological Society. 2014.* **20:** 1028-33.  Myers K, Cain S. 2008. Practice parameter for telepsychiatry with children and  adolescents. *Journal of the American Academy of Child and Adolescent Psychiatry*. **47:** 1468-83.  Myers K, Nelson EL, Rabinowitz T, Hilty D, Baker D, Barnwell SS, Boyce G, Bufka LF, Cain S, Chui L, Comer JS, Cradock C, Goldstein F, Johnston B, Krupinski E, Lo K, Luxton DD, McSwain SD, McWilliams J, North S, Ostrowsky J, Pignatiello A, Roth D, Shore J, Turvey C, Varrell JR, Wright S, Bernard J. 2017. American  Telemedicine Association Practice Guidelines for Telemental Health with  Children and Adolescents. *Telemedicine and e-Health.* **23:** 779-804.  Naskar S, Victor R, Das H, Nath K. 2017. Telepsychiatry  in India - Where Do We Stand? A Comparative Review between Global and Indian  Telepsychiatry Programs. *Indian Journal of Psychological Medicine.* **39:** 223-242.  Norman S. 2006.The use of telemedicine in psychiatry. *Journal of Psychiatric and Mental Health Nursing.* **13:** 771-777.  O'Brien M, McNicholas F. The Use of Telepsychiatry During COVID-19 and Beyond. *Irish Journal of Psychological Medicine.* 2020. **21:** 1-17.  O'Keefe M, White K, Jennings JC. 2019. Asynchronous telepsychiatry: A systematic review. *Journal of Telemedicine and Telecare.* **29**: 1357633X19867189  Odor, A., Yellowlees, P., Hilty, D., Parish, M.B., Nafiz, N., Iosif, A. 2011. PsychVACS: A system for asynchronous telepsychiatry. *Telemedicine and e-Health*. **17:** 299-303  Paing WW, Weller RA, Welsh B, Foster T, Birnkrant JM, Weller EB. Telemedicine  in children and adolescents. *Current Psychiatric Reports. 2009.* **11:** 114-119.  Ramalho R, Adiukwu F, Gashi Bytyçi D, El Hayek S, Gonzalez-Diaz JM, Larnaout A, Grandinetti P, Kundadak GK, Nofal M, Pereira-Sanchez V, Pinto da Costa M, Ransing R, Schuh Teixeira AL, Shalbafan M, Soler-Vidal J, Syarif Z, Orsolini L. Telepsychiatry and healthcare access inequities during  the COVID-19 pandemic. 2020. *Asian Journal of Psychiatry.* **53:** 102234.  Report from the Alberta Heritage Foundation for Medical Research. 1998. Evaluation of a telepsychiatry pilot project. *International Journal of Technology Assessment in Health Care*. **14:** 583-4.  Rothchild, E. Telepsychiatry: Why do it? *Psychiatric Annals*. 1999. **29:** 394-401.  Sabin JE, Skimming K. A framework of ethics for telepsychiatry practice. *International Review of Psychiatry. 2015;* **27:** 4 Yellowlees P, Richard Chan S, Burke Parish M. 2015. The hybrid doctor-patient relationship in the age of technology - Telepsychiatry consultations and the use of virtual space. International Review Psychiatry. 27: 476-489-495.  Samuels, A. 1999. International telepsychiatry: A link between New Zealand and Australia. *Australian and New Zealand Journal of Psychiatry*. **33:** 284-286.  Savin, D., Garry, M.T., Zuccaro, P., Novins, D. 2006. Telepsychiatry for treating rural American Indian Youth. *Journal of the American Academy of Child & Adolescent Psychiatry*. **45:** 484-488.  Seritan AL, Heiry M, Iosif AM, Dodge M, Ostrem JL. 2019. Telepsychiatry  for patients with movement disorders: a feasibility and patient satisfaction  study. *Journal of Clinical Movement Disorders.* **6:** 1.  Shore JH, Yellowlees P, Caudill R, Johnston B, Turvey C, Mishkind M, Krupinski E, Myers K, Shore P, Kaftarian E, Hilty D. 2018. Best Practices in Videoconferencing-Based Telemental Health April 2018. *Telemedicine and e-Health.* **24:** 827-832.  Shores, M.M., Ryan-Dykes, P., Williams, R.M., Mamerto, B., Sadak, T., Pascualy, M. et al. 2004. Identifying undiagnosed dementia in residential care veterans: comparing telemedicine to in-person clinical examination. *International Journal of Geriatric Psychiatry.* **19:** 101-108  Smith HA. 1999. Defending telepsychiatry. *Psychiatric Services.* **50:** 266-267. |
| Professional Guidance | Aboujaoude, E. Telemental health: Why the revolution has not arrived. *World Psychiatry.* 2018; **17:** 277-278  American Academy of Child and Adolescent Psychiatry (AACAP) Committee on Telepsychiatry and AACAP Committee on Quality Issues. 2017. Clinical update: Telepsychiatry with children and adolescents. *Journal of the American Academy of Child & Adolescent Psychiatry.* **56**: 875-893  Boydell KM, Hodgins M, Pignatiello A, Teshima J, Edwards H, Willis D. 2014. Using technology to deliver mental health services to children and youth: a scoping review. *Journal of the Canadian Academy of Child and Adolescent Psychiatry*. **23**:87-99.  Chipps J, Ramlall S, Mars M. 2012. Practice guidelines for videoconference-based telepsychiatry in South Africa. *African Journal of Psychiatry.* **15:** :271-82.  American Academy of Child and Adolescent Psychiatry (AACAP) Committee on Telepsychiatry and AACAP Committee on Quality Issues. 2017. Clinical Update: Telepsychiatry With Children and Adolescents. *Journal of the American Academy of Child & Adolescent Psychiatry.* **56:** 875-893  Ferrer DC, Yellowlees PM. 2012. Telepsychiatry: licensing and professional boundary  concerns. *Virtual Mentor*. **14**: 477-82.  Freudenberg N, Yellowlees PM. 2014. Telepsychiatry as Part of a Comprehensive Care Plan. *Virtual Mentor*. **16**: 964-8.  Gardner JS, Plaven BE, Yellowlees P, Shore JH. 2020. Remote Telepsychiatry Workforce: a Solution to Psychiatry's Workforce Issues. *Current Psychiatric Reports.* **22:** 8.  Grosch, M.C., Gottlieb, M.C., Cullum, C.M. Initial practice recommendations for teleneuropsychology. 2011. *The Clinical Neuropsychologist*. **25:** 1119-1133.  Hariman K, Ventriglio A, Bhugra D. 2019. The Future of Digital Psychiatry. *Current Psychiatry Reports.* **13:** 88.  Hilty DM, Luo, John S, Morache, Chris, Marcelo, Divine A & Nesbitt, Thomas S. 2002. Telepsychiatry: An overview for psychiatrists. *CNS Drugs.* **16:** 527-548.  Hilty DM, Marks, S.L., Urness, D., Yellowlees, P.M., Nesbitt, T.S.2004. Clinical and Educational Telepsychiatry Applications: A Review. *The Canadian Journal of Psychiatry / La Revue canadienne de psychiatrie*. **49:** 12-23.  Hilty DM, Yellowlees PM, Parrish MB, Chan S. 2015. Telepsychiatry: Effective, Evidence-Based, and at a Tipping Point in Health Care Delivery?. *Psychiatric Clinics of North America.* **38**: 559-92.  Hulsbosch, A.M., Nugter, M.A., Tamis, P., Kroon, H. 2017. Videoconferencing in a mental health service in The Netherlands: A randomized controlled trial on patient satisfaction and clinical outcomes for outpatients with severe mental illness. *Journal of Telemedicine and Telecare*. **23:** 513-520.  Jefee B.H., Mani N. International telepsychiatry: a review of what has been  published. *Journal of Telemedicine and Telecare*. **19:** 293-4.  Jones, Roland M, Leonard, S., Birmingham, L. 2006. Setting up a telepsychiatry service. *Psychiatric Bulletin*. **30:** 464-467  Lal, S., Abdel-Baki, A., Sujanani, S., Bourbeau, F., Sahed, I., Whitehead, J. 2020. Perspectives of young adults on receiving telepsychiatry services in an urban early intervention program for first-episode psychosis: A cross-sectional, descriptive survey study. *Frontiers in Psychiatry*. 11  Madhavan G. 2019. Telepsychiatry in intellectual disability psychiatry: literature  review. *BJPsych Bulletin.* **43**: 167-173  Malhotra S, Chakrabarti S, Shah R. 2013. Telepsychiatry:Promise, potential, and challenges. *Indian Journal of Psychiatry.* **55**: 3-11.  Math SB, Moirangthem S, Kumar NC. 2015. Tele-Psychiatry: After Mars, Can we Reach the Unreached?. *Indian Journal of Psychological Medicine.* **37:** 120-1.  McGrath J. 2020. ADHD and  Covid-19: Current roadblocks and future opportunities. *The Irish Journal of Psychological Medicine.* **21:** 1-22.  Clarke CS. 2018. Telepsychiatry in Asperger's syndrome. *Irish Journal of Psychological Medicine.* **35:** 325-328.  Whitten P, Kuwahara E. 2004. A multi-phase telepsychiatry programme in Michigan: organizational factors affecting utilization and user perceptions. *Journal of Telemedicine and Telecare.* **10:** 254-261.  Jones BN 3rd, Johnston D, Reboussin B, McCall WV. 2001. Reliability of telepsychiatry assessments: subjective versus observational ratings. *Journal of Geriatric Neurology and Psychiatry.* **14:** 66-71.  Saleem Y, Taylor MH, Khalifa N. 2008. Forensic telepsychiatry in the United Kingdom. *Behavioural Sciences and the Law*. **26:** 333-344.  Myers K. 2013. Telepsychiatry: time to connect. *Journal of the American Academy of Child and Adolescent Psychiatry.* **52:** 217-219.  Sharma A, Sasser T, Schoenfelder Gonzalez E, Vander Stoep A, Myers K.2020. Implementation of Home-Based Telemental Health in a Large Child Psychiatry Department During the COVID-19 Crisis. *Journal of Child and Adolescent Psychopharmacology.*  Frueh BC, Deitsch SE, Santos AB, Gold PB, Johnson MR, Meisler N, Magruder KM, Ballenger JC. 2000. Procedural and methodological issues in telepsychiatry research and program development. *Psychiatric Services.* **51:** 1522-1527.  Jones BN 3rd, Ruskin PE. 2001. Telemedicine and geriatric psychiatry: directions for future research and policy. *Journal of Geriatric Psychiatry and Neurology.* **14:** 59-62.  Monnier J, Knapp RG, Frueh BC. 2003. Recent advances in telepsychiatry: an updated review. *Psychiatric Services.* **54:** 1604-1609.  Hyler SE, Gangure DP. 2004. Legal and ethical challenges in telepsychiatry. *Journal of Psychiatric Practice.* **10:** 272-276.  Miller TW, Burton DC, Hill K, Luftman G, Veltkemp LJ, Swope M. 2005. Telepsychiatry: critical dimensions for forensic services. *Journal of Academic Psychiatry and Law.* **33:** 539-546.  Shore JH, Hilty DM, Yellowlees P. 2007. Emergency management guidelines for telepsychiatry. *General Hospital Psychiatry.* **29:** 199-206.  Pignatiello A, Teshima J, Boydell KM, Minden D, Volpe T, Braunberger PG. 2011. Child and youth telepsychiatry in rural and remote primary care. *Child and Adolescent Psychiatric Clinics in North America.* **20:** 13-28.  Shore JH. 2013. Telepsychiatry: videoconferencing in the delivery of psychiatric care. *American Journal of Psychiatry.* **170:** 256-262.  Salmoiraghi A, Hussain S. 2015. A Systematic Review of the Use of Telepsychiatry in Acute Settings. *Journal of Psychiatric Practice*. **21:** 389-393.  Nelson EL, Cain S, Sharp S. 2017. Considerations for Conducting Telemental Health with Children and Adolescents. *Child and Adolescent Psychiatric Clinics in North America.* **26:** 77-91.  Roth DE, Ramtekkar U, Zeković-Roth S. 2019. Telepsychiatry: A New Treatment Venue for Pediatric Depression. *Child and Adolescent Psychiatric Clinics in North America.* **28:** 377-395.  Smith K, Ostinelli E, Macdonald O, Cipriani A. 2020. COVID-19 and telepsychiatry: an evidence-based guidance for clinicians. *Journal of Medical Internet Research - International Scientific Journal for Medical Research.* **10.**  Fishkind, Avrim B & Cuyler, Robert N. 2013. The role of telepsychiatry. Zun, Leslie S [Ed], Chepenik, Lara G [Ed], Mallory, Mary Nan S [Ed]. Behavioral emergencies for the emergency physician. New York, NY, US: Cambridge University Press, US; pp. 303-307.  Yellowlees, Peter M, Hilty, Donald M & Mucic, Davor. 2016. Global/worldwide e-mental health: International and futuristic perspectives of telepsychiatry and the future. Mucic, Davor [Ed], Hilty, Donald M [Ed]. e-Mental health. Cham, Switzerland: Springer International Publishing, Switzerland; pp. 233-249  Vernig, P.M. 2016. Telemental health: Digital disruption and the opportunity to expand care. *Journal of the American Psychiatric Nurses Association*. **22:** 73-75.  Vanderpool, D. 2015. An overview of practicing high quality telepsychiatry. Dewan, Naakesh A [Ed], Luo, John S [Ed], Lorenzi, Nancy M [Ed]. Mental health practice in a digital world: A clinician's guide. Cham, Switzerland: Springer International Publishing, Switzerland; pp. 159-181  Valdagno M, Goracci A, di Volo S, Fagiolini A. 2014. Telepsychiatry: new perspectives and open issues. *CNS Spectrums.* **19:** 479-481.  Torous, J., Keshavan, M. & Gutheil, T. 2014. Promise and perils of digital psychiatry. *Asian Journal of Psychiatry.* **10:** 120-122.  Sunjaya AP, Chris A, Novianti D. 2020. Efficacy,  patient-doctor relationship, costs and benefits of utilizing telepsychiatry for  the management of post-traumatic stress disorder (PTSD): a systematic  review. *Trends in Psychiatry and Psychotherapy.* **42:** 102-110.  Starling, J., Dossetor, D. 2005. Child and Adolescent Telepsychiatry. Wootton, Richard [Ed], Batch, Jennifer [Ed]. Telepediatrics: Telemedicine and child health. Lo: Royal Society of Medicine Press; pp. 77-87.  Spaulding, R., Cain, S., Sonnenschein, K. 2011. Urban telepsychiatry: Uncommon service for a common need. *Child and Adolescent Psychiatric Clinics of North America.* **20:** 29-39  Sousa A, Karia S. 2020. Telepsychiatry  during COVID-19: Some clinical, public health, and ethical dilemmas. *Indian Journal of Public Health.* **64:** S245-S246.  Munro C.C., Hynan L.S, Grosch M, Parikh M, Weiner MF. 2014. Teleneuropsychology:  evidence for video teleconference-based neuropsychological assessment. *Journal of the International Neuropsychological Society.* **20:** 1028-33.  Myers K, Nelson EL, Rabinowitz T, Hilty D, Baker D, Barnwell SS, Boyce G, Bufka LF, Cain S, Chui L, Comer JS, Cradock C, Goldstein F, Johnston B, Krupinski E, Lo K, Luxton DD, McSwain SD, McWilliams J, North S, Ostrowsky J, Pignatiello A, Roth D, Shore J, Turvey C, Varrell JR, Wright S, Bernard J. 2017. American  Telemedicine Association Practice Guidelines for Telemental Health with  Children and Adolescents. *Telemedicine and e-Health.* **23:** 779-804.  Naskar S, Victor R, Das H, Nath K. Telepsychiatry in India - Where Do We Stand? A Comparative Review between Global and Indian  Telepsychiatry Programs. *Indian Journal of Psychological Medicine. 2017;* **39:** 223-242.  O'Brien M, McNicholas F. 2020. The Use of Telepsychiatry During COVID-19 and Beyond. *Irish Journal of Psychological Medicine.* **21:** 1-17.  Sales, C.P., McSweeney, L., Saleem, Y., Khalifa, N. 2018. The use of telepsychiatry within forensic practice: A literature review on the use of videolink-A ten-year follow-up. *Journal of Forensic Psychiatry & Psychology. 2018;*  **29:** 387-402.  Shore JH, Yellowlees P, Caudill R, Johnston B, Turvey C, Mishkind M, Krupinski E, Myers K, Shore P, Kaftarian E, Hilty D. 2018. Best Practices  in Videoconferencing-Based Telemental Health April 2018. *Telemedicine and e-Health.* **24:** 827-832. |

Appendix 2
Table 3: List of Studies

| **Authors** | **Date of publication** | **Type** | **Themes** |
| --- | --- | --- | --- |
| Adaji A, Fortney J. | 2017 | Narrative review | Diagnostic reliability Outcomes |
| Amirsadri A, Burns J, Pizzuti A, Arfken CL. | 2017 | Case report | Diagnostic reliability Patient & Clinical satisfaction |
| Balon R, Beresin EV, Coverdale JH, Louie AK, Roberts LW. | 2015 | Editorial | Diagnostic reliability |
| Bashshur RL, Shannon GW, Bashshur N, Yellowlees PM. | 2016 | Systematic review | Diagnostic reliability  Outcomes Patient & Clinical Satisfaction |
| Bishop J.E., O’Reilly R.L., Maddox K., Hutchinson L.J. | 2002 | RCT | Diagnostic reliability Outcomes Patient & Clinical satisfaction |
| Boydell KM, Hodgins M, Pignatiello A, Teshima J, Edwards H, Willis D | 2014 | Literature review | Diagnostic reliability  Outcomes  Patient & Clinical satisfaction  Technology Professional Guidance |
| Campbell R, O'Gorman J, Cernovsky ZZ. | 2015 | Qualitative | Diagnostic reliability  Patient & Clinical satisfaction  Technology |
| Chakrabarti S. | 2015 | Literature Review | Diagnostic reliability  Outcomes  Patient & Clinical satisfaction  Technology |
| Chipps J, Brysiewicz P, Mars M. | 2012 | Systematic review | Diagnostic reliability  Outcomes  Patient & Clinical satisfaction |
| Chipps J, Ramlall S, Madigoe T, King H, Mars M. | 2012 | Service evaluation | Diagnostic reliability  Outcomes  Patient & Clinical satisfaction  Technology |
| De Las Cuevas C, A.J, De La Fuente J., Serrano P. | 2003 | Qualitative | Diagnostic reliability  Patient & Clinical satisfaction |
| Deslich S, Stec B, Tomblin S, Coustasse A. | 2013 | Literature review | Diagnostic reliability  Outcomes  Patient & Clinical satisfaction  Technology |
| Diamond, J.M., Bloch, R.M. | 2010 | Literature review | Diagnostic reliability  Outcomes  Patient & Clinical satisfaction |
| Gammon D, Bergvik S, Bergmo T, Pedersen S. | 1996 | Qualitative | Diagnostic reliability  Patient & Clinical satisfaction  Technology |
| Elford DR, White H, St John K, Maddigan B, Ghandi M, Bowering R. | 2001 | Qualitative | Diagnostic reliability  Outcomes  Patient & Clinical satisfaction  Technology |
| Elford R., White H., Bowering R., Ghandi A., Maddiggan B., St John K., House M., Harnett J., West R., Battcock A. | 2000 | RCT | Diagnostic reliability  Outcomes  Patient & Clinical satisfaction  Technology |
| Glueck DA. | 2011 | Literature review | Diagnostic reliability  Outcomes  Patient & Clinical satisfaction  Technology |
| Green AS, Ruchman SG, Katz CL, Singer EK. | 2020 | Service evaluation | Diagnostic reliability |
| Gunter, T.D, Srinivasaraghavan, J. Terry, N.P. | 2003 | Editorial | Diagnostic reliability  Patient & Clinical satisfaction |
| Grosch, M.C., Gottlieb, M.C., Cullum, C.M. | 2011 | Commentary | Diagnostic reliability  Professional Guidance |
| Hailey D, Jacobs P, Simpson J, Doze S. | 1999 | Commentary | Diagnostic reliability  Patient & Clinical satisfaction  Technology |
| Hariman K, Ventriglio A, Bhugra D. | 2019 | Narrative review | Diagnostic reliability  Outcomes  Patient & Clinical satisfaction  Professional Guidance |
| Hilt, R.J. | 2017 | Narrative review | Diagnostic reliability  Outcomes  Technology |
| Hilty DM Johnston, B McCarron, R.M. | 2016 | Book chapter | Diagnostic reliability  Outcomes |
| Hilty DM, Bourgeois, J.A., Nesbitt, T.S., Hales, R.E. | 2004 | Literature Review | Diagnostic reliability  Outcomes |
| Hilty DM, Ferrer DC, Parish MB, Johnston B, Callahan EJ, Yellowlees PM | 2013 | Literature review | Diagnostic reliability  Outcomes  Patient & Clinical satisfaction  Technology |
| Hilty DM, Luo, John S, Morache, Chris, Marcelo, Divine A & Nesbitt, Thomas S. | 2002 | Literature Review | Diagnostic reliability  Outcomes  Patient & Clinical satisfaction  Technology  Professional guidance |
| Hilty DM, Marks, S.L., Urness, D., Yellowlees, P.M., Nesbitt, T.S. | 2004 | Literature review | Diagnostic reliability  Outcomes  Technology  Professional Guidance |
| Hubley S, Lynch SB, Schneck C, Thomas M, Shore J | 2016 | Systematic review | Diagnostic reliability  Outcomes  Patient & Clinical satisfaction |
| Jacob MK, Larson JC, Craighead WE | 2012 | Service evaluation | Diagnostic reliability  Outcomes  Patient & Clinical Satisfaction  Technology |
| Jones, B.N | 2002 | Editorial | Diagnostic reliability |
| Kaftarian E. | 2019 | Narrative review | Diagnostic reliability  Outcomes  Patient & Clinical satisfaction  Technology |
| Khalifa, N, Saleem, Y., Stankard, P. | 2008 | Literature review | Diagnostic reliability  Outcomes  Patient & Clinical satisfaction  Technology |
| Lau, M.E., Way, B.B., Fremont, W.P. | 2011 | Service evaluation | Diagnostic reliability  Outcomes  Patient & Clinical satisfaction |
| Lee A, S.N., O'Connell F, Dyer A, Boniface K, Betz J. | 2015 | Case report | Diagnostic reliability  Technology |
| Leonard S. | 2004 | Service evaluation | Diagnostic reliability  Technology |
| Lexcen, F.J., Hawk, G.L., Herrick, S., Blank, M.B | 2006 | Prospective | Diagnostic reliability  Technology |
| Litwack, S.D., Jackson, C.E., Chen, M., Sloan, D.M., Hatgis, C., Litz, B.T. et al. | 2014 | Prospective | Diagnostic reliability  Patient & Clinical satisfaction |
| Loh, P. K, Maher, S., Goldswain, P., Flicker, L., Ramesh, P., Saligari, J. | 2005 | Letter to the Editor | Diagnostic reliability |
| Loh, P.K., Donaldson, M., Flicker, L., Maher, Sean S., Goldswain, P. | 2007 | Prospective | Diagnostic reliability |
| Malhotra S, Chakrabarti S, Shah R | 2013 | Literature review | Diagnostic reliability  Patient & Clinical satisfaction  Outcomes  Technology  Professional Guidance |
| Martin-Khan, M., Wootton, R., Whited, J., Gray, L.C | 2011 | Systematic review | Diagnostic reliability |
| Mazhari S, Ghaffari Nejad A, Mofakhami O, Raaii F, Bahaadinbeigy K | 2019 | Prospective | Diagnostic reliability  Outcomes  Patient & clinician satisfaction |
| Menon, A. Srikumar, K., Prasad, K., Popuri, Chrismer, J.B., Raskin, A., Hebel, J.R. et al. | 2001 | Service evaluation | Diagnostic reliability  Patient & clinician satisfaction  Technology |
| Heravian A, Chang BP. | 2018 | Letter to the Editor | Diagnostic reliability  Patient & clinical satisfaction |
| Matsuura S, Hosaka T, Yukiyama T, Ogushi Y, Okada Y, Haruki Y, Nakamura M. | 2000 | Prospective | Diagnostic reliability  Patient & clinician satisfaction  Technology |
| Brodey BB, Claypoole KH, Motto J, Arias RG, Goss R | 2000 | Qualitative | Diagnostic reliability  Outcomes  Patient & clinician satisfaction |
| Jones BN 3rd, Johnston D, Reboussin B, McCall WV. | 2001 | Prospective | Diagnostic reliability  Technology  Professional Guidance |
| Rohland BM. | 2001 | Prospective | Diagnostic reliability  Outcomes  Patient & clinician satisfaction |
| Shore JH, Savin D, Orton H, Beals J, Manson SM. | 2007 | Prospective | Diagnostic reliability |
| Singh SP, Arya D, Peters T. | 2007 | Prospective | Diagnostic reliability  Outcomes |
| Myers KM, Palmer NB, Geyer JR. | 2011 | Literature review | Diagnostic reliability  Outcomes  Technology |
| Savin D, Glueck DA, Chardavoyne J, Yager J, Novins DK. | 2011 | Literature review | Diagnostic reliability  Technology |
| Myers K. | 2013 | Literature review | Diagnostic reliability  Outcomes  Patient & clinician satisfaction  Technology  Professional guidance |
| Nassan M, Frye MA, Adi A, Alarcón RD | 2015 | Correspondence | Diagnostic reliability  Patient & clinician satisfaction |
| Das S, Manjunatha N, Kumar CN, Math SB, Thirthalli J | 2020 | Prospective | Diagnostic reliability  Patient & clinician satisfaction |
| Sharma A, Sasser T, Schoenfelder Gonzalez E, Vander Stoep A, Myers K. | 2020 | Service evaluation | Diagnostic reliability  Outcomes  Technology  Professional guidance |
| Frueh BC, Deitsch SE, Santos AB, Gold PB, Johnson MR, Meisler N, Magruder KM, Ballenger JC. | 2000 | Literature review | Diagnostic reliability  Outcomes  Patient & clinician satisfaction  Technology  Professional Guidance |
| Jones BN 3rd, Ruskin PE. | 2001 | Commentary | Diagnostic reliability  Outcomes  Technology  Professional Guidance |
| Monnier J, Knapp RG, Frueh BC. | 2003 | Literature review | Diagnostic reliability  Outcomes  Patient & clinician satisfaction  Technology  Professional guidance |
| Modai I, Jabarin M, Kurs R, Barak P, Hanan I, Kitain L | 2007 | Prospective | Diagnostic reliability  Outcomes  Patient & clinician satisfaction |
| Ramos-Ríos R, Mateos R, Lojo D, Conn DK, Patterson T | 2012 | Literature review | Diagnostic reliability  Patient & clinician satisfaction  Outcomes  Technology |
| Yellowlees P, Richard Chan S, Burke Parish M. | 2015 | Narrative review | Diagnostic reliability  Outcomes  Patient & clinician satisfaction |
| Fortney JC, Pyne JM, Turner EE, Farris KM, Normoyle TM, Avery MD, Hilty DM, Unützer J | 2015 | Narrative review | Diagnostic reliability  Outcomes  Patient & clinician satisfaction |
| Roth DE, Ramtekkar U, Zeković-Roth S. | 2019 | Narrative review | Diagnostic reliability  Patient & clinician satisfaction  Professional guidance |
| Farabee D., Calhoun S., Veliz R. | 2016 | RCT | Diagnostic reliability  Outcomes |
| O’Reilly R., Bishop J., Maddox K., Hutchinson L., Fisman M., Takhar J. | 2007 | RCT | Diagnostic reliability  Outcomes  Patient & clinician satisfaction |
| Fishkind, Avrim B & Cuyler, Robert N | 2013 | Book chapter | Diagnostic reliability  Outcomes  Patient & clinician satisfaction  Professional guidance |
| Alessi N | 2002 | Letter to the Editor | Diagnostic reliability |
| Jones BN 3rd. | 2001 | Narrative review | Diagnostic reliability  Outcomes  Patient & clinician satisfaction |
| Zaylor C. | 1999 | Retrospective study | Diagnostic reliability  Outcomes |
| Zaylor C, Nelson EL, Cook DJ. | 2001 | Service evaluation | Diagnostic reliability  Patient & clinician satisfaction |
| Yellowlees, Peter M, Hilty, Donald M, Marks, Shayna L, Neufeld, Jonathan & Bourgeois, James A. | 2008 | Retrospective study | Diagnostic reliability  Outcomes |
| Work Group on Quality Issues (WGQI) & American Academy of Child and Adolescent Psychiatry (AACAP), US. | 2008 | Guideline | Diagnostic reliability  Outcomes  Patient & clinician satisfaction  Technology |
| Volicer L. | 2015 | Editorial | Diagnostic reliability  Patient & clinician satisfaction |
| van Wynsberghe A, Gastmans C. | 2009 | Literature review | Diagnostic reliability  Outcomes  Patient & clinician satisfaction |
| Sunjaya AP, Chris A, Novianti D. | 2020 | Systematic review | Diagnostic reliability  Outcomes  Professional guidance |
| Starling, J., Dossetor, D. | 2005 | Book chapter | Diagnostic reliability  Outcomes  Patient & clinician satisfaction  Technology  Professional guidance |
| Munro C.C., Hynan L.S, Grosch M, Parikh M, Weiner MF. | 2014 | Prospective | Diagnostic reliability  Technology  Professional guidance |
| Nelson EL, Zaylor C, Cook D. | 2004 | Prospective | Diagnostic reliability |
| Saeed SA, Anand V. | 2015 | Commentary | Diagnostic reliability  Outcomes |
| Setterberg, S.R., Busseri, M.A., Fleissner, R.M., Kenney, E.M., Flom, J.A., Fischer, K.J | 2003 | Service evaluation | Diagnostic reliability  Outcomes |
| Shores, M.M., Ryan-Dykes, P., Williams, R.M., Mamerto, B., Sadak, T., Pascualy, M. et al | 2004 | Prospective | Diagnostic reliability  Patient & clinician satisfaction  Technology |
| Aboujaoude, E., Gega. | 2020 | Commentary | Outcomes  Patient & clinician satisfaction |
| Abrams J, Sossong S, Schwamm LH, Barsanti L, Carter M, Kling N, Kotarski M, Leddy J, Meller B, Simoni M, Sullivan M, Wozniak J. | 2017 | Literature review | Outcomes  Technology |
| American Academy of Child and Adolescent Psychiatry (AACAP) Committee on Telepsychiatry and AACAP Committee on Quality Issues. | 2017 | Systematic review | Outcomes  Patient & clinician satisfaction  Technology  Professional Guidance |
| Barrera-Valencia C, Benito-Devia AV, Vélez-Álvarez C, Figueroa-Barrera M, Franco-Idárraga SM. | 2017 | Economic study | Outcomes |
| Batastini, Ashley B, McDonald, Brendan R & Morgan, Robert D. | 2013 | Book chapter | Outcomes  Patient & clinician satisfaction  Technology |
| Behere PB, Mansharamani HD, Kumar K | 2017 | Commentary | Outcomes  Patient & clinician satisfaction |
| Bahloul, H.J., Mani, N. | 2013 | Correspondence | Outcomes  Patient & clinician satisfaction |
| Bolle R.R., Trondsen M.V., Stensland G.Ø., Tjora A. | 2018 | Qualitative | Outcomes  Patient & clinician satisfaction |
| Borders C.B. | 2017 | Commentary | Outcomes  Patient & clinician satisfaction |
| Buist A, Coman G, Silvas A, Burrows G. | 2000 | Qualitative | Outcomes  Patient & clinician satisfaction  Technology |
| Butterfield, A. | 2018 | Literature review | Outcomes  Patient & clinician satisfaction |
| Chan S, Parish M, Yellowlees P. | 2015 | Narrative review | Outcomes  Patient & clinician satisfaction  Technology |
| Chan SR, Torous J, Hinton L, Yellowlees P. | 2014 | Narrative review | Outcomes |
| Cheng KM, Siu BW, Au Yeung CC, Chiang TP, So MH, Yeung MC. | 2018 | Case-control study | Outcomes  Patient & clinician satisfaction |
| Caudill, R.L., Sager, Z. | 2015 | Narrative review | Outcomes  Patient & clinician satisfaction  Technology |
| Chong, J., Moreno, F. | 2012 | RCT | Outcomes  Patient & clinician satisfaction  Technology |
| Cowan KE, McKean AJ, Gentry MT, Hilty DM. | 2019 | Literature review | Outcomes  Patient & clinician satisfaction  Technology |
| Crowe T, Jani S, Jani S, Jani N, Jani R. | 2016 | RCT | Outcomes  Patient & clinician satisfaction |
| Deslich SA, Thistlethwaite T, Coustasse A. | 2013 | Literature review | Outcomes  Patient & clinician satisfaction  Technology |
| Detweiler MB, Arif S, Candelario J, Altman J, Murphy PF, Halling MH, Detweiler JG, Vasudeva S. | 2011 | Retrospective study | Outcomes  Patient & clinical satisfaction  Technology |
| Donley, E., McClaren, A., Jones, R., Katz, P., Goh, J. | 2017 | Service evaluation | Outcomes  Patient & clinician satisfaction  Technology |
| Dossetor, D. R, Nunn, K. P, Fairley, M., Eggleton, D. | 1999 | Service evaluation | Outcomes  Patient & clinician satisfaction |
| Egede LE, Frueh CB, Richardson LK, Acierno R, Mauldin PD, Knapp RG, Lejuez C. | 2009 | Proposal | Outcomes  Patient & clinician satisfaction |
| Ellington, E., Repique, R., John R. | 2013 | Commentary | Outcomes |
| Flaum MA. | 2017 | Editorial | Outcomes |
| Freudenberg N, Yellowlees PM | 2014 | Commentary | Outcomes  Professional guidance |
| Chipps J, Ramlall S, Mars M. | 2012 | Commentary | Outcomes  Patient & clinician satisfaction  Technology  Professional guidance |
| Gardner JS, Plaven BE, Yellowlees P, Shore JH. | 2020 | Commentary | Outcomes  Patient & clinician satisfaction  Technology  Professional guidance |
| Doze S, Simpson J, Hailey D, Jacobs P. | 1999 | Service evaluation | Outcomes  Patient & clinician satisfaction |
| Gentile, J.P, Cowan, A.E., Harper, B., Mast, R., Merrill, B. | 2018 | Commentary | Outcome |
| Gloff, N.E, LeNoue, S.R, Novins, D.K., Myers, K. | 2015 | Literature review | Outcomes  Patient & clinician satisfaction  Technology |
| Gopalan P, Shenai N, Dunn S, Bilderback A. | 2020 | Letter to the Editor | Outcomes |
| Gowda, Guru S, Kulkarni, Karishma, Bagewadi, Virupaksha, R. P. S., Shyam, Manjunatha, B. R, Shashidhara, Harihara N, et al | 2018 | Retrospective study | Outcomes  Patient & clinician satisfaction  Technology |
| Grady B. | 2012 | Commentary | Outcomes  Technology |
| Haghnia Y., Samad-Soltani T., Yousefi M., Sadr H., Rezaei-Hachesu P. | 2019 | RCT | Outcomes  Patient & clinician satisfaction  Technology |
| Graham, M.A. | 1996 | Commentary | Outcomes  Technology |
| Hilty DM, Cobb, H.C., Neufeld, J.D., Bourgeois, J.A., Yellowlees, P.M. | 2008 | Economic study | Outcomes |
| Hilty DM, Crawford A, Teshima J, Chan S, Sunderji N, Yellowlees PM, Kramer G, O'neill P, Fore C, Luo J, Li ST. | 2015 | Guidelines | Outcomes  Patient & clinician satisfaction |
| Hilty DM, Sunderji, N., Suo, S., Chan, S., McCarron, R.M. | 2018 | Narrative review | Outcomes |
| Hilty DM, Yellowlees PM, Parrish MB, Chan S. | 2015 | Narrative review | Outcomes  Patient & clinician satisfaction  Professional guidance |
| Hungerbuehler I, Valiengo L, Loch AA, Rössler W, Gattaz WF. | 2016 | RCT | Outcomes  Patient & clinician satisfaction  Technology |
| Jefee B.H., Mani N. | 2013 | Correspondence | Outcomes  Patient & clinician satisfaction  Professional guidance |
| Jefee-Bahloul H. | 2014 | Commentary | Outcomes  Patient & clinician satisfaction  Technology |
| Hensel J, Graham R, Isaak C, Ahmed N, Sareen J, Bolton J. | 2020 | Qualitative | Outcomes  Patient & clinician satisfaction |
| Hilty DM, Mucic, D. | 2016 | Book chapter | Outcomes |
| Keilman, P. | 2005 | Qualitative | Outcomes  Patient & clinician satisfaction |
| Kennedy C, Yellowlees P. | 2000 | Service evaluation | Outcomes  Patient & clinician satisfaction  Technology |
| Koblauch, H., Reinhardt, S.M., Lissau, W.J. & Jensen, P. | 2018 | Systematic review | Outcomes |
| Kornbluh RA. | 2014 | Commentary | Outcomes  Patient & clinician satisfaction |
| Krzystanek M, Krysta K, Skałacka K. | 2017 | Prospective study | Outcomes |
| Krzystanek, M., Krzeszowski, D., Jagoda, K., Krysta, K. | 2015 | Prospective study | Outcomes |
| LaBelle B, Franklyn AM, Pkh Nguyen V, Anderson KE, Eibl JK, Marsh DC. | 2018 | Retrospective study | Outcomes  Patient & clinician satisfaction  Technology |
| Lal, S., Abdel-Baki, A., Sujanani, S., Bourbeau, F., Sahed, I., Whitehead, J. | 2020 | Qualitative | Outcomes  Patient & clinician satisfaction  Technology  Professional guidance |
| Madhavan G. | 2019 | Literature Review | Outcomes  Patient & clinician satisfaction  Technology  Professional guidance |
| Mahmoud H, Vogt EL, Dahdouh R, Raymond ML. | 2020 | Case Report | Outcomes |
| Malhotra, S., Shah, R. | 2018 | Book chapter | Outcomes  Patient & clinician satisfaction  Technology |
| Math SB, Moirangthem S, Kumar NC. | 2015 | Commentary | Outcomes  Technology  Professional guidance |
| McGrath J. | 2020 | Commentary | Outcomes  Technology  Professional guidance |
| McLaren P. | 2004 | Commentary | Outcomes  Patient & clinician satisfaction  Technology |
| Mettner J. | 2013 | Commentary | Outcomes  Patient & clinician satisfaction  Technology |
| Mielonen, M., Ohinmaa, A., Moring, J., Isohanni, M. | 2002 | Commentary | Outcomes  Patient & clinician satisfaction  Technology |
| Clarke CS. | 2018 | Case report | Outcomes  Patient & clinician satisfaction  Technology  Professional guidance |
| Ikelheimer, D.M. | 2008 | Letter to the Editor | Outcomes  Patient & clinician satisfaction  Technology |
| Benyakorn S. | 2016 | Narrative review | Outcomes  Patient & clinician satisfaction  Technology |
| Hilty DM., Yellowlees, P.M. | 2015 | Commentary | Outcomes  Patient & clinician satisfaction |
| Gabel S. | 2009 | Letter to the Editor | Outcomes  Patient & clinician satisfaction  Technology |
| Whitten P, Kuwahara E. | 2004 | Qualitative | Outcomes  Patient & clinician satisfaction  Professional guidance |
| Narasimhan M, Druss BG, Hockenberry JM, Royer J, Weiss P, Glick G, Marcus SC, Magill J. | 2015 | Service evaluation | Outcomes  Patient & clinical satisfaction |
| Fox KC, Connor P, McCullers E, Waters T. | 2008 | Retrospective study | Outcomes  Patient & clinical satisfaction |
| Szeftel R, Federico C, Hakak R, Szeftel Z, Jacobson M. | 2012 | Retrospective study | Outcomes |
| Ulzen T, Williamson L, Foster PP, Parris-Barnes K. | 2013 | Service evaluation | Outcomes  Patient & clinical satisfaction |
| McGinty KL, Saeed SA, Simmons SC, Yildirim Y. | 2006 | Commentary | Outcomes  Patient & clinical satisfaction  Technology |
| Urness D, Wass M, Gordon A, Tian E, Bulger T. | 2006 | Qualitative | Outcomes  Patient & clinical satisfaction |
| Yellowlees P, Burke MM, Marks SL, Hilty DM, Shore JH. | 2008 | Commentary | Outcomes  Patient & clinical satisfaction |
| García-Lizana F, Muñoz-Mayorga I. | 2010 | Systematic review | Outcomes  Patient & clinical satisfaction  Technology |
| Myers KM, Vander Stoep A, McCarty CA, Klein JB, Palmer NB, Geyer JR, Melzer SM. | 2010 | Service evaluation | Outcomes  Patient & clinical satisfaction |
| Szeftel R, Mandelbaum S, Sulman-Smith H, Naqvi S, Lawrence L, Szeftel Z, Coleman S, Gross L. | 2011 | Commentary | Outcomes  Patient & clinical satisfaction |
| Grady BJ, Lever N, Cunningham D, Stephan S. | 2011 | Literature review | Outcomes  Patient & clinical satisfaction  Technology |
| Shim R, Ye J, Yun K. | 2012 | Service evaluation | Outcomes |
| Shore JH. | 2013 | Commentary | Outcomes  Patient & clinical satisfaction  Technology  Professional guidance |
| Salmoiraghi A, Hussain S. | 2015 | Systematic review | Outcomes  Patient & clinical satisfaction  Technology  Professional guidance |
| Shore J. | 2015 | Narrative review | Outcomes  Patient & clinical satisfaction |
| Lauckner C, Whitten P. | 2016 | Literature review | Outcomes  Patient & clinical satisfaction |
| Nelson EL, Cain S, Sharp S. | 2017 | Commentary | Outcomes  Patient & clinical satisfaction  Technology  Professional guidance |
| Roberts N, Hu T, Axas N, Repetti L. | 2017 | Prospective study | Outcomes  Patient & clinical satisfaction  Technology |
| Swanson CL, Trestman RL. | 2018 | Commentary | Outcomes  Patient & clinical satisfaction |
| Serhal E, Crawford A, Cheng J, Kurdyak P. | 2017 | Epidemiological | Outcomes |
| Hassan A, Sharif K. | 2019 | Systematic review | Outcomes  Patient & clinical satisfaction |
| Smith K, Ostinelli E, Macdonald O, Cipriani A. | 2020 | Systematic review | Outcomes  Patient & clinical satisfaction  Professional guidance |
| Myers K., Vander Stoep A., McCarty C.A., Katon W. | 2015 | RCT | Outcomes  Patient & clinical satisfaction |
| Shulman M., John M., Kane J.M. | 2017 | RCT | Outcomes  Patient & clinical satisfaction  Technology |
| Saurman E, Lyle D, Perkins D, Roberts R. | 2014 | Service evaluation | Outcomes |
| Mahmoud H, Vogt E. | 2019 | Narrative review | Outcomes  Patient & clinical satisfaction  Technology |
| Yilmaz SK, Horn BP, Fore C, Bonham CA. | 2019 | Economic study | Outcomes |
| Yellowlees, Peter M, Hilty, Donald M & Mucic, Davor. | 2016 | Book chapter | Outcomes  Patient & clinical satisfaction  Professional guidance |
| Wojtuszek, Magdalena, Kachnic, Justyna, Krysta, Krzysztof & Wutke, Joanna | 2015 | Qualitative | Outcomes  Technology |
| Wallace, Duncan & Hodges, Samantha. | 2018 | Service evaluation | Outcomes  Patient & clinical satisfaction |
| Ventriglio, Antonio, T.J., Castaldelli-Maia, J. | 2017 | Commentary | Outcomes |
| Vanderpool, D. | 2015 | Book chapter | Outcomes  Technology  Professional guidance |
| Vander S.A.,Myers K. | 2013 | ‘Lessons learnt’ from RCT | Outcomes |
| Valdagno M, Goracci A, di Volo S, Fagiolini A. | 2014 | Literature review | Outcomes  Patient & clinical satisfaction  Professional guidance |
| Trondsen MV, Bolle SR, Stensland GØ, Tjora A. | 2014 | Qualitative study | Outcomes  Patient & clinical satisfaction |
| Thompson D.A., Leimig R., Gower G., Winsett R.P. | 2009 | Service evaluation | Outcomes  Patient & clinical satisfaction |
| Thomas RK, Suleman R, Mackay M, Hayer L, Singh M, Correll CU, Dursun S. | 2020 | Editorial | Outcomes  Technology |
| Tang S, Helmeste D. | 2000 | Commentary | Patient & clinical satisfaction  Technology |
| Thomas JF, Novins DK, Hosokawa PW, Olson CA, Hunter D, Brent AS, Frunzi G, Libby AM. | 2018 | Service evaluation | Outcomes  Patient & clinical satisfaction |
| Thiele, J.S., Doarn, C.R., Shore, J.H. | 2015 | Literature review | Outcomes |
| Sulzbacher, S.,, Vallin, T., Waetzig, E.Z. | 2006 | Commentary | Outcomes  Patient & clinical satisfaction |
| Stankard, P., Younus, S. | 2007 | Letter to the Editor | Outcomes |
| Spaulding, R., Cain, S., Sonnenschein, K. | 2011 | Service evaluation | Outcomes  Patient & clinical satisfaction  Technology  Professional guidance |
| Moirangthem S, Rao S, Kumar CN, Narayana M, Raviprakash N, Math SB. | 2017 | Retrospective study | Outcomes  Technology |
| Pelton D, Wangelin B, Tuerk P. | 2015 | Case Report | Outcomes |
| Reliford A, Adebanjo B. | 2019 | Cross-sectional | Outcomes  Patient & clinical satisfaction |
| Rockhill, C.M., Tse, Y.J., Fesinmeyer, M.D., Garcia, J., & Myers, K. | 2016 | RCT | Outcomes |
| Ruskin PE, Silver-Aylaian M, Kling MA, Reed SA, Bradham DD, Hebel JR, Barrett D, Knowles F 3rd, Hauser P. | 2004 | RCT | Outcomes |
| McLaren P, Ahlbom J, Riley A, Mohammedali A, Denis M. | 2002 | Pilot study | Outcomes  Patient & clinical satisfaction |
| Sales, C.P., McSweeney, L., Saleem, Y., Khalifa, N. | 2018 | Literature review | Outcomes  Patient & clinical satisfaction  Professional guidance |
| No authors listed. | 2011 | Commentary | Patient & clinical satisfaction |
| Aadil M, Cosme RM, Forcen FE, Khan AR. | 2017 | Editorial | Patient & clinical satisfaction |
| Abba-Aji, A. | 2006 | Commentary | Patient & clinical satisfaction |
| Abdi YA, Elmi JY. | 2011 | Commentary | Patient & clinical satisfaction |
| Agarwal PP, Manjunatha N, Gowda GS, Kumar MNG, Shanthaveeranna N, Kumar CN, Math SB. | 2019 | Retrospective study | Patient & clinical satisfaction  Technology |
| Alexander J, Lattanzio A. | 2009 | Correspondence | Patient & clinical satisfaction |
| Ben-Zeev D. | 2020 | Commentary | Patient & clinical satisfaction |
| Blackmon, L.A., Kaak, H.O., Ranseen, J | 1997 | Qualitative study | Patient & clinical satisfaction |
| Boydell KM, Volpe T, Pignatiello A. | 2010 | Qualitative study | Patient & clinical satisfaction |
| Boydell, K.M., Volpe, T., Kertes, A., Greenberg, N. | 2007 | Retrospective study | Patient & clinical satisfaction  Technology |
| Rachal, J., Sparks, W., Zazzaro C., Blackwell T. | 2015 | Commentary | Patient & clinical satisfaction |
| Cerda, G.M., Hilty, D.M., Hales, R.E., Nesbitt, T.S. | 1999 | Letter to the Editor | Patient & clinical satisfaction |
| Corruble E. | 2020 | Commentary | Patient & clinical satisfaction |
| Cruz, M.K., Elizabeth A., Lopez, A.M., Weinstein, R.S. | 2005 | Retrospective study | Patient & clinician satisfaction |
| Dham, P., Gupta, N., Alexander, J., Black, W., Rajji, T., Skinner, E. | 2018 | Retrospective study | Patient & clinical satisfaction  Technology |
| Ermer D.J. | 1999 | Commentary | Patient & clinical satisfaction |
| Gibson, K., O'Donnell, S., Coulson, H., Kakepetum-Schultz, T. | 2011 | Qualitative study | Patient & clinical satisfaction |
| Gelber H. | 2001 | Qualitative study | Patient & clinical satisfaction  Technology |
| Gratzer D, Torous J, Lam RW, Patten SB, Kutcher S, Chan S, Vigo D, Pajer K, Yatham LN. | 2020 | Editorial | Patient & clinical satisfaction |
| Grubaugh, Anouk L, Cain, Gregory D, Elhai, Jon D, Patrick, Sarah L & Frueh, B. Christopher. | 2008 | Qualitative study | Patient & clinical satisfaction  Technology |
| Harley, J., McLaren, P., Blackwood, G., Tierney, K., Everett, M. | 2002 | Qualitative | Patient & clinical satisfaction |
| Harley, J. | 2006 | Economic study | Patient & clinical satisfaction |
| Hasselberg MJ. | 2020 | Narrative review | Patient & clinical satisfaction |
| Hilty DM, Gentry MT, McKean AJ, Cowan KE, Lim RF, Lu FG. | 2020 | Scoping review | Patient & clinical satisfaction |
| Hockey AD, Yellowlees PM, Murphy S. | 2004 | Service evaluation | Patient & clinical satisfaction |
| Hulsbosch, A.M., Nugter, M.A., Tamis, P., Kroon, H. | 2017 | RCT | Patient & clinical satisfaction  Professional guidance |
| Hungerbuehler I., Leite R.F.M., van de Bilt M.T., Gattaz W.F. | 2015 | RCT | Patient & clinical satisfaction |
| Jones, Roland M, Leonard, S., Birmingham, L. | 2006 | Commentary | Patient & clinical satisfaction  Technology  Professional guidance |
| Kalin ML, Garlow SJ, Thertus K, Peterson MJ. | 2020 | Letter to the Editor | Patient & clinical satisfaction |
| Karlinsky, Harry. | 2004 | Editorial | Patient & clinical satisfaction |
| Katz CL, Washington FB, Sacco M, Schuetz-Mueller J. | 2019 | Service evaluation | Patient & clinical satisfaction  Technology |
| Kavanagh S, Hawker F. | 2001 | Service evaluation | Patient & clinical satisfaction |
| Kimmel, R.J., Toor, R. | 2019 | Service evaluation | Patient & clinical satisfaction |
| Kopel H, Nunn K, Dossetor D. | 2001 | Qualitative study | Patient & clinical study  Technology |
| Lin, C., Bai, Y., Chen, J. | 2003 | Letter to the Editor | Patient & clinical satisfaction |
| Lingley-Pottie, P., McGrath, P.J. | 2008 | Commentary | Patient & clinical satisfaction |
| Mannion, L, Fahy, T. J, Duffy, C, Broderick, M., Gethins, E. | 1998 | Service evaluation | Patient & clinical satisfaction  Technology |
| May CR, Ellis NT, Atkinson T, Gask L, Mair F, Smith C. | 1999 | Service evaluation | Patient & clinical satisfaction  Technology |
| McCann RA, Erickson JM, Palm-Cruz KJ. | 2020 | Case report | Patient & clinical satisfaction |
| Meltzer, B. | 1997 | Commentary | Patient & clinical satisfaction  Technology |
| Greenwood, J., Chamberlain, C., Parker, G. | 2004 | Service evaluation | Patient & clinical satisfaction |
| Mucic D. | 2008 | Qualitative | Patient & clinical satisfaction  Technology |
| Myers KM, Valentine JM, Melzer SM. | 2008 | Service evaluation | Patient & clinical satisfaction |
| Nelson EL, Bui TN, Velasquez SE. | 2011 | Literature review | Patient & clinical satisfaction |
| Ye J, Shim R, Lukaszewski T, Yun K, Kim SH, Ruth G. | 2012 | Qualitative study | Patient & clinical satisfaction  Technology |
| May C, Gask L, Ellis N, Atkinson T, Mair F, Smith C, Pidd S, Esmail A | 2000 | Qualitative study | Patient & clinical satisfaction |
| Rohland BM, Saleh SS, Rohrer JE, Romitti PA. | 2000 | Qualitative study | Patient & clinical satisfaction |
| May C, Gask L, Atkinson T, Ellis N, Mair F, Esmail A. | 2001 | Ethnographic | Patient & clinical satisfaction  Technology |
| Johnston D, Jones BN 3rd. | 2001 | Service evaluation | Patient & clinical satisfaction  Technology |
| Pollard SE, LePage JP. | 2001 | Letter to the Editor | Patient & clinical satisfaction |
| Krupinski EA, Barker G, Lopez AM, Weinstein RS. | 2004 | Retrospective study | Patient & clinical satisfaction |
| Myers K, Valentine J, Morganthaler R, Melzer S. | 2006 | Retrospective study | Patient & clinical satisfaction |
| Saleem Y, Taylor MH, Khalifa N. | 2008 | Overview | Patient & clinical satisfaction  Professional guidance |
| Wallace D, Rayner S. | 2013 | Service evaluation | Patient & clinical satisfaction |
| Jefee-Bahloul H. | 2014 | Service evaluation | Patient & clinical satisfaction  Technology |
| Myers KM, Valentine JM, Melzer SM. | 2007 | Observational | Patient & clinical satisfaction |
| Pignatiello A, Teshima J, Boydell KM, Minden D, Volpe T, Braunberger PG. | 2011 | Service evaluation | Patient & clinical satisfaction  Professional guidance |
| Wood J, Stathis S, Smith A, Krause J. | 2012 | Service evaluation | Patient & clinical satisfaction |
| Ellington E. | 2013 | Qualitative | Patient & clinical satisfaction |
| Simpson J, Doze S, Urness D, Hailey D, Jacobs P | 2001 | Service evaluation | Patient & clinical satisfaction  Technology |
| Zaylor C, Whitten P, Kingsley C. | 2000 | Service evaluation | Patient & clinical satisfaction |
| Yellowlees P, Nakagawa K, Pakyurek M, Hanson A, Elder J, Kales HC. | 2020 | Service evaluation | Patient & clinical satisfaction  Technology |
| Wilshire, Thea W. | 2012 | Service evaluation | Patient & clinical satisfaction |
| Whaibeh, Emile, Mahmoud, Hossam & Vogt, Emily L. | 2019 | Commentary | Patient & clinical satisfaction |
| Vought, Rhonda G, Grigsby, R. Kevin, Adams, Laura N & Shevitz, Stewart A. | 2000 | Service evaluation | Patient & clinical satisfaction |
| Volpe T, Boydell KM, Pignatiello A. | 2013 | Service evaluation | Patient & clinical satisfaction  Technology |
| Vernig, P.M. | 2016 | Commentary | Patient & clinical satisfaction  Technology  Professional guidance |
| Turner, J.W. | 2001 | Qualitative | Patient & clinical satisfaction |
| Toperczer T. | 2011 | Commentary | Patient & clinical satisfaction |
| Toombs E, Kowatch KR, Dalicandro L, McConkey S, Hopkins C, Mushquash CJ. | 2020 | Systematic review | Patient & clinical satisfaction  Technology |
| Taylor M, Kikkawa N, Hoehn E, Haydon H, Neuhaus M, Smith AC, Caffery LJ | 2019 | Qualitative | Patient & clinical satisfaction |
| Swanson B. | 1999 | Commentary | Patient & clinical satisfaction |
| Stevens, A., Doidge, N., Goldbloom, D., Voore, P., Farewell, J. | 1999 | Qualitative | Patient & clinical satisfaction |
| Starling, J., Foley, S. | 2006 | Service evaluation | Patient & clinical satisfaction |
| Sousa A, Karia S. | 2020 | Letter to the Editor | Patient & clinical satisfaction  Technology  Professional guidance |
| Mucic, D. | 2009 | Book chapter | Patient & clinical satisfaction |
| Pakyurek, M, Yellowlees, P., Hilty, D. | 2010 | Commentary | Patient & clinical satisfaction |
| Pesämaa L, Ebeling H, Kuusimäki ML, Winblad I, Isohanni M, Moilanen I. | 2004 | Systematic review | Patient & clinical satisfaction |
| Reinhardt I, Gouzoulis-Mayfrank E, Zielasek J. | 2019 | Systematic review | Patient & clinical satisfaction |
| Report from the Alberta Heritage Foundation for Medical Research. | 1998 | Service evaluation | Patient & clinical satisfaction  Technology |
| Rowe N, Gibson S, Morley S, Krupinski EA. | 2008 | Longitudinal | Patient & clinical satisfaction |
| Samuels, A. | 1999 | Letter to the Editor | Patient & clinical satisfaction  Technology |
| Savin, D., Garry, M.T., Zuccaro, P., Novins, D. | 2006 | Case Report | Patient & clinical satisfaction  Technology |
| Schubert NJ, Backman PJ, Bhatla R, Corace KM. | 2019 | Cross-sectional | Patient & clinical satisfaction |
| Seritan AL, Heiry M, Iosif AM, Dodge M, Ostrem JL. | 2019 | Feasibility study | Patient & clinical satisfaction  Technology |
| Simpson J, Doze S, Urness D, Hailey D, Jacobs P. | 2001 | Cross-sectional | Patient & clinical satisfaction |
| Aboujaoude, E. | 2018 | Commentary | Technology  Professional guidance |
| Augusterfer EF, Mollica RF, Lavelle J. | 2018 | Literature Review | Technology |
| Barnett, Michael L & Huskamp, Haiden A. | 2020 | Commentary | Technology |
| Brown FW. | 1998 | Commentary | Technology |
| Chipps J, Ramlall S, Mars M. | 2012 | Commentary | Technology |
| Fatehi, F., Armfield, N.R., Dimitrijevic, M., Gray, L.C. | 2014 | Scoping review | Technology |
| Fegert JM, Vitiello B, Plener PL, Clemens V. | 2020 | Narrative review | Technology |
| Fortney JC, Heagerty PJ, Bauer AM, Cerimele JM, Kaysen D, Pfeiffer PN, Zielinski MJ, Pyne JM, Bowen D, Russo J, Ferro L, Moore D, Nolan JP, Fee FC, Heral T, Freyholtz-London J, McDonald B, Mullins J, Hafer E, Solberg L, Unützer J. | 2020 | Description of creating a study | Technology |
| Gelber H, Alexander M. | 1999 | Service evaluation | Technology |
| Hailey, David, Ohinmaa, Arto & Roine, Risto. | 2009 | Systematic review | Technology |
| Hampton, T. | 2006 | Commentary | Technology |
| Haslam R, McLaren P | 2000 | Service evaluation | Technology |
| Kannarkat JT, Smith NN, McLeod-Bryant SA. | 2020 | Commentary | Technology |
| Langarizadeh M, Tabatabaei MS, Tavakol K, Naghipour M, Rostami A, Moghbeli F | 2017 | Systematic review | Technology |
| Looi JC, Pring W. | 2020 | Service evaluation | Technology |
| Werner A, Anderson LE. | 1998 | Commentary | Technology |
| Miller TW, Clark J, Veltkamp LJ, Burton DC, Swope M. | 2008 | Service evaluation | Technology |
| Westphal A, Dingjan P, Attoe R. | 2010 | Literature review | Technology |
| Mars M. | 2012 | Commentary | Technology |
| Miller TW, Burton DC, Hill K, Luftman G, Veltkemp LJ, Swope M. | 2005 | Service evaluation | Technology  Professional guidance |
| Nieves JE, Stack KM. | 2007 | Letter to the Editor | Technology |
| Saeed SA. | 2018 | Service evaluation | Technology |
| Zulfic Z, Liu D, Lloyd C, Rowan J, Schubert KO. | 2020 | Letter to the Editor | Technology |
| Vega, W.A., Pollitt, A., Mays, R.A. | 2017 | Correspondence | Technology |
| Myers K, Cain S. | 2008 | Guidelines | Technology |
| Myers K, Nelson EL, Rabinowitz T, Hilty D, Baker D, Barnwell SS, Boyce G, Bufka LF, Cain S, Chui L, Comer JS, Cradock C, Goldstein F, Johnston B, Krupinski E, Lo Ke, Luxton DD, McSwain SD, McWilliams J, North S, Ostrowsky J, Pignatiello A, Roth D, Shore J, Turvey C, Varrell JR, Wright S, Bernard J. | 2017 | Guidelines | Technology  Professional guidance |
| Naskar S, Victor R, Das H, Nath K. | 2017 | Narrative review | Technology  Professional guidance |
| Norman S. | 2006 | Systematic review | Technology |
| O'Brien M, McNicholas F. | 2020 | Commentary | Technology  Professional guidance |
| Paing WW, Weller RA, Welsh B, Foster T, Birnkrant JM, Weller EB. | 2009 | Commentary | Technology |
| Ramalho R, Adiukwu F, Gashi Bytyçi D, El Hayek S, Gonzalez-Diaz JM, Larnaout A, Grandinetti P, Kundadak GK, Nofal M, Pereira-Sanchez V, Pinto da Costa M, Ransing R, Schuh Teixeira AL, Shalbafan M, Soler-Vidal J, Syarif Z, Orsolini L. | 2020 | Letter to the Editor | Technology |
| Rothchild, E. | 1999 | Commentary | Technology |
| Sabin JE, Skimming K. | 2015 | Commentary | Technology |
| Shore JH, Yellowlees P, Caudill R, Johnston B, Turvey C, Mishkind M, Krupinski E, Myers K, Shore P, Kaftarian E, Hilty D. | 2018 | Commentary | Technology  Professional guidance |
| Smith HA. | 1999 | Letter to the Editor | Technology |
| Ferrer DC, Yellowlees PM. | 2012 | Commentary | Professional guidance |
| Hyler SE, Gangure DP. | 2004 | Commentary | Professional guidance |
| Shore JH, Hilty DM, Yellowlees P. | 2007 | Commentary | Professional guidance |
| Torous, J., Keshavan, M. & Gutheil, T. | 2014 | Commentary | Professional guidance |

### Appendix 3: Thematic Analysis Methodology

The researchers undertook an inductive thematic analysis outlined by Nowell et al. in their 2017 paper [107], illustrated by a six-step process outlined below.

1. Familiarization with data
Both authors read through the data twice and actively searched for patterns related to clinical effectiveness (deductive analysis). These patterns were documented with direct quotations from the data.

2. Generation of initial codes
The authors compared patterns to identify similarities and differences in recurring patterns as well as identifying any potential researcher bias. These recurring patterns generated the initial codes of the study.

3. Searching for themes
The authors identified and agreed on recurring themes in the data, structuring the data into themes relevant to the research question: the clinical effectiveness of telepsychiatry.

4. Reviewing the themes
Themes were reviewed to test for referential adequacy by returning to the raw data and ensuring the themes were valid and grounded in the data. This resulted in some themes being removed and condensed.

5. Defining and naming themes
The authors discussed the themes that were identified, formulating the narrative about the clinical effectiveness of telepsychiatry based on direct quotations from the data as well as overall patterns of these themes within the literature.

6. Producing the report
The authors expanded on each theme, including the information and context provided by the data as well as their clinical implications on the clinical effectiveness of telepsychiatry.

*
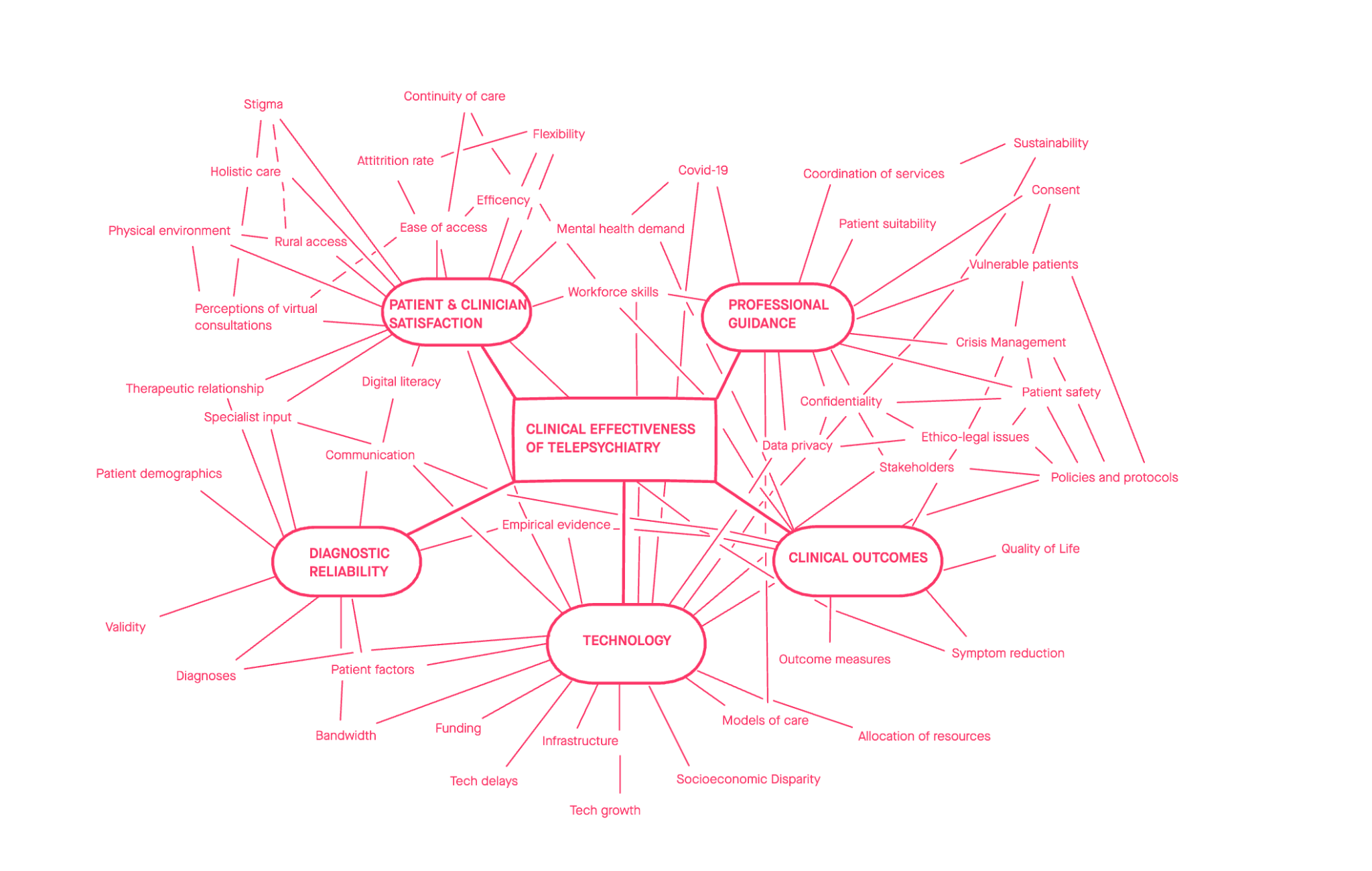
*

*Figure 3: Mindmap illustrating development of themes*
